# Calcium-Release Channels in *Paramecium*. Genomic Expansion, Differential Positioning and Partial Transcriptional Elimination

**DOI:** 10.1371/journal.pone.0027111

**Published:** 2011-11-10

**Authors:** Eva-Maria Ladenburger, Helmut Plattner

**Affiliations:** Department of Biology, University of Konstanz, Konstanz, Germany; University of Georgia, United States of America

## Abstract

The release of Ca^2+^ from internal stores is a major source of signal Ca^2+^ in almost all cell types. The internal Ca^2+^ pools are activated via two main families of intracellular Ca^2+^-release channels, the ryanodine and the inositol 1,4,5-trisphosphate (InsP_3_) receptors. Among multicellular organisms these channel types are ubiquitous, whereas in most unicellular eukaryotes the identification of orthologs is impaired probably due to evolutionary sequence divergence. However, the ciliated protozoan *Paramecium* allowed us to prognosticate six groups, with a total of 34 genes, encoding proteins with characteristics typical of InsP_3_ and ryanodine receptors by BLAST search of the *Paramecium* database. We here report that these Ca^2+^-release channels may display all or only some of the characteristics of canonical InsP_3_ and ryanodine receptors. In all cases, prediction methods indicate the presence of six trans-membrane regions in the C-terminal domains, thus corresponding to canonical InsP_3_ receptors, while a sequence homologous to the InsP_3_-binding domain is present only in some types. Only two types have been analyzed in detail previously. We now show, by using antibodies and eventually by green fluorescent protein labeling, that the members of all six groups localize to distinct organelles known to participate in vesicle trafficking and, thus, may provide Ca^2+^ for local membrane-membrane interactions. Whole genome duplication can explain radiation within the six groups. Comparative and evolutionary evaluation suggests derivation from a common ancestor of canonical InsP_3_ and ryanodine receptors. With one group we could ascertain, to our knowledge for the first time, aberrant splicing in one thoroughly analyzed *Paramecium* gene. This yields truncated forms and, thus, may indicate a way to pseudogene formation. No comparable analysis is available for any other, free-living or parasitic/pathogenic protozoan.

## Introduction

Calcium serves as a second messenger in all eukaryotes, from man [1]–[4] to protozoa, including ciliates, such as *Paramecium* [5]. Ca^2+^ may govern widely different processes, such as exocytosis, endocytosis and phagocytosis, cell movement, cytokinesis, morphogenesis, gene transcription etc. The signaling effect is antagonized not only by rapid dissipation, binding to Ca^2+^-binding and effector proteins, sequestration and extrusion [2], [3], but also by supralinear dependency on local Ca^2+^ concentration, [Ca^2+^]_i_ [6].

Cells dispose not only of Ca^2+^ influx channels, but also of different intracellular Ca^2+^-release channels (CRCs) in different regions [1]. Among them are two main families, the inositol 1,4,5-trisphosphate (InsP_3_) and the ryanodine receptors. Functional channels are tetrameric encompassing per subunit a size of ∼300 kDa and ∼500 kDa, respectively. Their highly conserved C-terminal channel domains possess six transmembrane domains for InsP_3_ receptors [7]. The situation with ryanodine receptors is less clear, as four to 12 transmembrane segments are discussed [8]–[10], [10]–[13]. We took particular efforts to prognosticate with high probability the number of transmembrane domains in our CRCs by using different prediction algorithms. Besides the conserved channel domains, InsP_3_ and ryanodine receptors share further common domains designated as ryanodine/InsP_3_ receptor homology domain (RIH) and MIR (protein mannosyltransferase, InsP_3_ and ryanodine receptor) domain which is also found in protein mannosyltransferases [14]. Both channel types need Ca^2+^ as co-activator but are regulated by different endogenous agonists and show differences in pharmacology [15], [16]. The InsP_3_ receptor is responsive to the second messenger InsP_3_ which binds to an N-terminal InsP_3_-binding domain [17]. In mammalian cells, ryanodine receptors are activated by the alkaloid ryanodine, which is inhibitory at >10 µM concentration [18], by cyclic adenosine diphosphate-ribose [13], 4-chloro-m-cresol [19] and by caffeine [20]. Their activation requires sub-millimolar 4-CmC or caffeine in tens of millimolar concentrations. The latter is an effective inhibitor of InsP_3_ dependent Ca^2+^-release [21].

**Figure 1:**
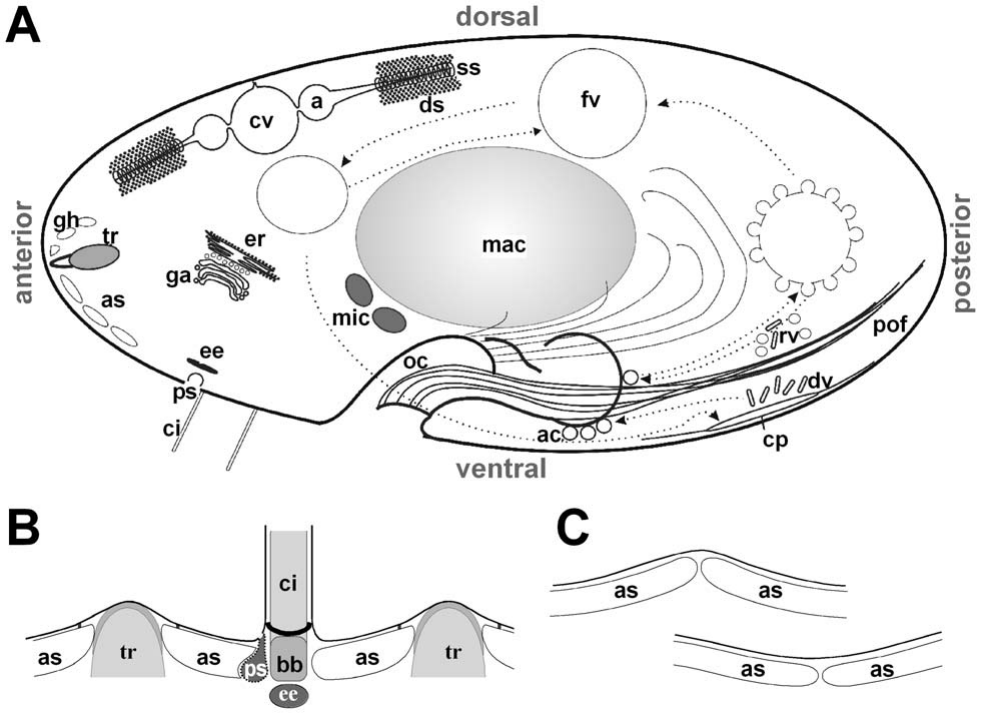
Cellular components of *Paramecium* - conserved and specialized organelles. (A) Scheme of a *Paramecium* cell (based on Allen and Fok, 2000 [26], Plattner and Kissmehl, 2003 [33]). The cell is lined with cortical Ca^2+^-stores, the alveolar sacs (as) attached to the cell membrane, from where cilia (ci) and dense-core secretory vesicles (trichocysts, tr) emerge. “gh” indicates membrane “ghosts” from released trichocysts. Also outlined are components of the phagocytic cycle, starting with the terminal invagination of the oral cavity (oc), the cytopharynx, from where food vacuoles (fv) are released. Acidosomes (as), discoidal vesicles (dv) and other recycling vesicles (rv) deliver membrane material for phagosome formation. Defecation occurs at the cytoproct (cp). The endosomal system is represented by parasomal sacs (ps; corresponding to coated pits) and early endosomes (ee; designated as terminal cisternae). Additionally, the Golgi apparatus (ga), the endoplasmic reticulum (er) and the two distinct types of nuclei, the germline micronuclei (mic) and the somatic macronucleus (mac) are outlined. Also included are elements of the contractile vacuole complex with the contractile vacuole (cv), the ampullae (a), the decorated spongiome (ds) and the smooth spongiome (ss). Cell length is ∼100 to 120 µm. (B) In detail the cortical organization contains alternating arrangements of trichocysts (tr), cilia (ci) and basal bodies (bb) as well as alveolar sacs (as). Those underlie the plasma membrane, except at sites occupied by trichocysts and cilia. Parasomal sacs (ps) are positioned at the anterior sites of cilia, whereas early endosomes (ee) are located below basal bodies. (C) Adjacent alveolar sacs are in contact with each other at longitudinal and perpendicular elevations of the cell surface (upper graph) and at depressions (lower graph) in the vicinity of cilia.

Very surprisingly, despite some efforts particularly with parasitic forms, no molecular identification of InsP_3_ and ryanodine receptors has been achieved with any protozoa or plants [22], [23]. An exception are two CRCs from *Paramecium*, one of the InsP_3_ receptor [24], and the other one of the ryanodine receptor type [25]. We now were able to complement the list of CRCs, or of CRC-like proteins, in *Paramecium*. The large size of these cells (∼100 to 120 µm) combined with an elaborate regular design facilitates localization studies. They also possess rather pronounced vesicular trafficking pathways [26]. As shown in Figure 1, this includes preformed clathrin-coated endocytosis sites (parasomal sacs) near ciliary bases, early endosomes (terminal cisternae) below ciliary basal bodies, sites for stimulated dense core vesicle (trichocyst) exocytosis, phagosomes of different maturation stages, sites for defecation of spent phagosomes (cytoproct), recycling vesicles originating from the cytoproct and from mature phagosomes etc. Flat sacs tightly apposed to the cell membrane (alveolar sacs) serve as cortical Ca^2+^ stores [27] which are mobilized upon exocytosis stimulation [28], [29]. The entire surface of a *Paramecium* cell is shaped like an egg-case, with longitudinal and perpendicular ridges whose units (kinetids) contain the cortical structures just described. The cytostome, i.e. the outer part of the oral cavity, contains not only cilia for ingesting food bacteria into the nascent phagosome, but also alveolar sacs, endocytosis sites and early endosomes in a rather dense packing [30]. Vesicles of different types deliver membrane materials for forming the vacuole which then travels through the cell (cyclosis), with input from lysosomes and output of recycling vesicles [26]. Finally, the contractile vacuole complex represents a complicated membrane system serving not only osmoregulation in this freshwater organism [31] but also extrusion of an excess of Ca^2+^ [32].

There are tens of different types of membrane interactions in a *Paramecium* cell [33]. In higher eukaryotes, requirement of Ca^2+^ for membrane interactions, particularly for membrane fusion, is established not only for exocytosis, endocytosis and phagocytosis, but also for a variety of other membrane interactions [34]. Some of the intracellular trafficking vesicles deeper inside the cell contain themselves Ca^2+^ [35], [36] which they may release, unless Ca^2+^ originates from the classical store, the endoplasmic reticulum (ER). Local regulation by topologically diversified CRCs may be an adjustment to local requirements.

We now followed these interesting aspects in *Paramecium* cells. Previously we could describe only two types of CRCs. One is a true InsP_3_ receptor, localized to the contractile vacuole complex where it may serve fine-tuning of [Ca^2+^]_i_ [24]. The other one is of a mixed type: It contains no InsP_3_-binding domain but six trans-membrane domains and, by its activation by caffeine and 4-CmC, resembles more a ryanodine receptor. It releases Ca^2+^ upon stimulation of trichocyst exocytosis [25]. Now we could expand the repertoire of CRCs in *Paramecium* by four more groups, yielding a total of six groups (CRC-I to CRC-VI) consisting of different subfamilies (e.g. CRC-I-1) with a total of 34 members (e.g. CRC-I-1 with ohnologs CRC-I-1a and CRC-I-1b). We find the different CRCs or CRC-like channels expressed at different intracellular sites known to undergo vesicle trafficking and membrane interactions. Another aspect is the first demonstration in *Paramecium* of aberrant splicing in one of the CRC genes which yields an abortive product, possibly revealing a way toward transformation into a pseudogene.

For identification, based on scaffolds from the *Paramecium* database (DB) [37], we made BLAST searches, analysis of characteristic domain structure, including RIH, InsP_3_-binding domain, transmembrane domains and pore domains. Very strikingly, some of the characteristics known from their mammalian counterparts are missing in these sequences homologous to CRCs which, therefore, may also be considered CRC-like channels. Although much more work will be required for their thorough characterization, the important point is the unequivocal identification of continuous sequences of genes for homologs of InsP_3_ and ryanodine receptor-type proteins whose expression and intracellular localization we have also analyzed. For localization and assignment to different vesicle populations we applied monospecific antibodies and microtubule staining which highlights the regular arrangement of surface structures to which the molecules of interest can be related. These methodical aspects in combination are the salient features of the present work. It contrasts with the lack of detailed information on any other protozoa, despite extensive and competent search.

## Results

**Figure 2:**
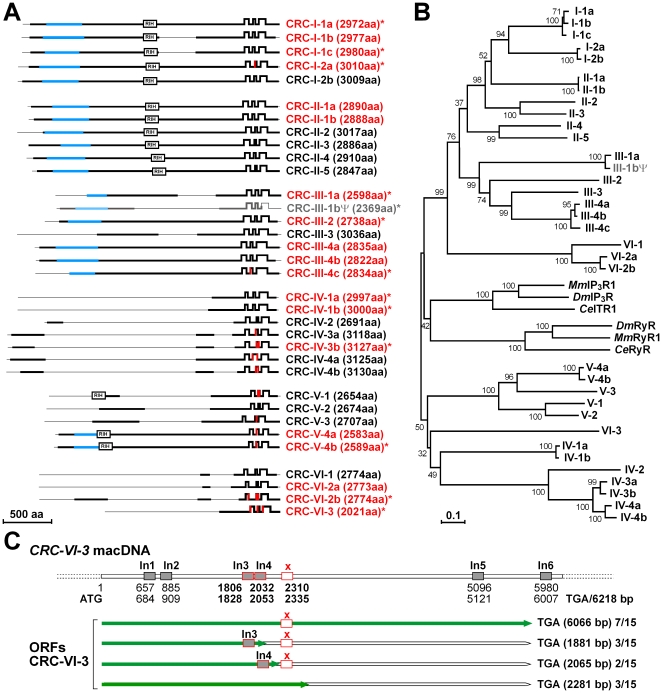
Survey of the 34 *Paramecium* CRC proteins. (A) Domain arrangement of putative Ca^2+^-release channels in *Paramecium*. Transmembrane helices were predicted using TOPCONS [38], TMHMM (v2) [39] and Kyte Doolitte hydrophobicity scales [40] and are indicated as serrated motifs (positions of flanking residues are outlined in Table S1). These C-terminal parts display a six membrane spanning topology resembling channel domains of mammalian InsP_3_ receptors. Membrane-spanning segments with varying positioning are in red. Conserved regions are highlighted in black, those corresponding to the InsP_3_-binding domains of mammalian InsP_3_ receptors in blue. Also shown are the RIH according to Ponting [14]. CRCs printed in red were analyzed on RNA level, those marked with an asterisk deviate, after re-annotation, from the sequences published in the *Paramecium*DB (http://paramecium.cgm.cnrs-gif.fr). CRC-III-1bΨ (printed in grey) was identified as a pseudogene (see Figure S1E). Accession numbers for all CRC genes are summarized in Table 1. (B) Phylogeny of the C-terminal channel domains of *Paramecium* CRCs. Predictions from multiple sequence alignments are shown in a neighbor-joining tree with 1000 bootstrap replicates generated with the MEGA version 3.0 program [98]. Bootstrap support values for the nodes are shown and an evolutionary distance scale is given by the scale bar below. Besides the transmembrane domain-coding sequences of *Paramecium* CRCs (CRC-I-1a [I2475-P2910]; CRC-I-1b [I2480-P2915]; CRC-I-1c [I2483-P2918]; CRC-I-2a [I2527-P2959]; CRC-I-2b [I2526-P2958]; CRC-II-1a [I2379-P2815]; CRC-II-1b [E2377-P2813]; CRC-II-2 [V2530-P2965]; CRC-II-3 [V2385-P2826]; CRC-II-4 [I2415-P2854]; CRC-II-5 [I2370-P2799]; CRC-III-1a [V2106-P2553]; CRC-III-1bΨ [V2106-V2369]; CRC-III-2 [I2209-P2688]; CRC-III-3 [I2519-P2984]; CRC-III-4a [I2289-P2773]; CRC-III-4b [I2276-P2760]; CRC-III-4c [I2288-P2772]; CRC-IV-1a [I2527-P2965]; CRC-IV-1b [I2531-P2968]; CRC-IV-2 [I2219-P2675]; CRC-IV-3a [I2643-P3098]; CRC-IV-3b [I2652-P3107]; CRC-IV-4a [I2649-P3102]; CRC-IV-4b [I2654-P3107]; CRC-V-1 [I2233-P2621]; CRC-V-2 [I2252-P2640]; CRC-V-3 [I2184-I2632]; CRC-V-4a [I2146-P2529]; CRC-V-4b [I2152-P2551]; CRC-VI-1 [I2272-P2727]; CRC-VI-2a [I2284-P2733]; CRC-VI-2b [I2285-P2734]; CRC-VI-3 [I2583-P2010]) we included the channel domains from mouse (*Mus musculus*) *Mm*InsP_3_R type 1 (I2195-L2675; Acc No: NP_034715.2); *Drosophila melanogaster Dm*InsP_3_R (I2286-P2762; Acc No: BAA14399.1) and *Caenorhabditis elegans Ce*ITR1 (I2321-P2793; Acc No NP_001023173) as well as corresponding regions from *Mm*RyR type 1 (E4481-P5021; Acc No: NP_033135); *Dm*RyR (E4553-P5092; Acc No: NP_033135) and *Ce*RyR (P4539-P5055; Acc No: BAA08309). (C) Schematic representation of the *CRC-VI-3* gene. Start (1) and stop codons (6218) of *CRC-VI-3*, as well as the positions of six introns (In1 to In6) were determined by RT-PCR. cDNA analyses revealed four different open reading frames (ORFs; green lines). Full-length transcripts result in an ORF of 6063 bp encoding a 2021 amino acid polypeptide (seven out of 15 clones). We found three additional shorter forms resulting from aberrant splicing of intron 3 and 4 (three and two out of 15 clones, respectively) and a 26 bp fragment (x), respectively. Fragment x was found to be mistakenly spliced in several clones (3 out of 15 clones).

In order to specify the 34 *Paramecium* CRCs in more detail, we selected one subfamily within one group of channels for detailed analyses. As shown in Figure 2A, 20 genes were analyzed on cDNA level and sequencing reveals that all of them are transcribed. The open reading frames (ORFs) of 13 CRCs show deviations from the predicted ORFs in the *Paramecium*DB with regard to the positioning of introns and translational start and/or stop sites. Re-annotations of the respective CRCs are outlined in Figure S1; accession numbers are summarized in Table 1.

**Table 1:** CRCs in *Paramecium tetraurelia*.

Protein	Accession number(GenBank)	Scaffold	Length (aa)	Identities (%)(full-length analyses)			Identities (%)(C-terminal domain)		
				A	B	C	a	b	c
CRC-I-1a	FR877768*	9	2972	100	100	17	100	28	24
CRC-I-1b	CAK73596	26	2977	94	94	17	96	28	23
CRC-I-1c	FR877769*	98	2980	81	81	16	94	28	24
CRC-I-2a	FR877770*	20	3010	33	33	16	53	28	24
CRC-I-2b	CAK76340	32	3009	33	33	16	52	28	23
CRC-II-1a	CAI39149	48	2890	100	33	15	48	26	22
CRC-II-1b	CAI39148	34	2888	93	34	15	48	26	23
CRC-II-2	CAK75723	30	3017	27	27	15	47	25	22
CRC-II-3	CAK65009	144	2886	27	27	17	50	29	24
CRC-II-4	CAK55585	1	2910	25	24	16	42	29	23
CRC-II-5	CAK90437	79	2847	24	24	17	38	29	25
CRC-III-1a	FR877771*	86	2598	100	21	14	32	20	21
CRC-III-1bΨ	CAK67044	158	2369	92	21	14	32	21	21
CRC-III-2	FR877772*	35	2738	24	22	16	34	24	21
CRC-III-3	CAK75361	3B	3036	27	22	15	40	25	21
CRC-III-4a	CAK81773	49	2835	26	22	16	37	23	23
CRC-III-4b	CAK68868	17	2822	26	22	16	37	23	22
CRC-III-4c	FR877773*	37	2834	27	21	16	37	23	22
CRC-IV-1a	PTETG13800001001**	138	2997	100	14	13	27	30	26
CRC-IV-1b	BN001236	80	3000	90	15	13	27	30	26
CRC-IV-2	CAK93344	9	2691	14/100	13	15	21	24	20
CRC-IV-3a	CAK86172	62	3118	14/38	12	14	24	24	21
CRC-IV-3b	FR877774*	24	3127	13/38	13	14	24	24	21
CRC-IV-4a	CAK85149	6	3125	14/38	13	15	22	22	19
CRC-IV-4b	CAK75306	3	3130	13/38	13	14	22	21	19
CRC-V-1	CAK70871	2	2645	100	15	15	27	25	22
CRC-V-2	CAK74967	3	2674	62	16	15	29	25	23
CRC-V-3	CAK78094	38	2707	31	15	15	22	25	20
CRC-V-4a	CAK94510	96	2583	30	15	17	25	25	22
CRC-V-4b	FR877775*	106	2589	31	15	17	24	25	22
CRC-VI-1	CAK80449	44	2774	100	16	13	29	25	17
CRC-VI-2a	CAK69911	18	2773	44	17	13	30	24	17
CRC-VI-2b	FR877776*	44	2774	44	17	12	31	25	17
CRC-VI-3	FR877777*	134	2021	15	14	12	24	24	18

*Re-annotated CRC sequences were submitted to the EMBL database as TPA (third party annotation) entry. Sequences are available upon publication.

**At present only accession numbers from the ParameciumDB (http://paramecium.cgm.cnrs-gif.fr/) are available for CRC-IV-1a sequences. Accession numbers from GeneBank are in progress.

CRC-III-1b, printed in grey, is a pseudogene. Scaffold denotes genomic regions according to macronuclear DNA sections as designated in the *Paramecium*DB.

(A) Sequence comparisons within one group. Alignments were performed by ClustalW.

Identities of full-length proteins referring to CRC-I-1a (B) or to IP_3_R type 1 from *Mus musculus* (C).

Identities of C-terminal channel domains (sections are outlined in legend of figure 2B) referring to corresponding sequences of CRC-I-1a (a), of mouse IP_3_R type 1 (b) or of mouse RyR type 1 (c).

### Overall characterization of *Paramecium* CRCs

**Figure 3:**
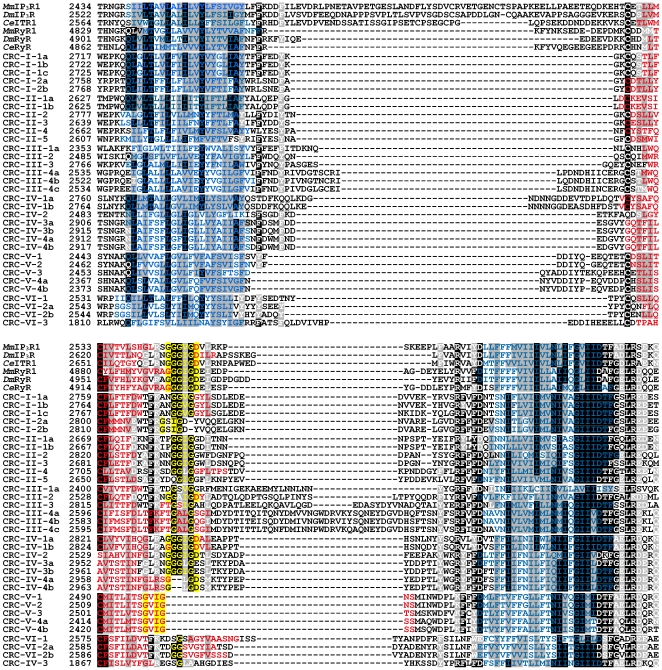
Sequence alignment of putative pore regions with adjacent transmembrane segments. Sequences covering TMD5 and TMD6 of *Paramecium* CRCs were aligned with corresponding regions of metazoan InsP_3_ and ryanodine receptors from mouse (*Mm*), *D. melanogaster* (*Dm*) and *C. elegans* (*Ce*) using the ClustalW algorithm. Sequences are shown in single-letter code and are numbered on the left side. Identical residues are shaded in black, similar residues in gray. Amino acids corresponding to putative selectivity filter regions [45] are highlighted in yellow within the pore region (red). Transmembrane regions (blue) were predicted using the web server TOPCONS [38].

The 34 re-annotated CRCs were further analyzed by focusing on the C-terminal domains of the proteins implying putative membrane spanning helices involved in Ca^2+^ channeling. Prediction studies were performed on the sequences of full-length CRCs and on their C-terminal parts (see Table S1) employing various transmembrane prediction methods: TOPCONS [38], TMHMM (v2) [39] and Kyte Doolitte hydrophobicity scales [40]. The graph in Figure 2A summarizes CRC membrane predictions obtained with TOPCONS (http://topcons.net/; [38]), an algorithm which integrates five different topology predictions (OCTOPUS [41], PRO-TMHMM and PRODIV-TMHMM [42], SCAMPI-single and SCAMPI-multi [43] into one consensus prediction, by computing a reliable score based on the agreement of the five methods across the sequence combined with a Hidden Markov Model (HMM). The results suggest that all *Paramecium* CRCs possess an overall six-membrane spanning topology, thereby reflecting the topology of the channel-domain of metazoan InsP_3_ receptors. According to their architecture, the pore region resides between the fifth and sixth membrane-spanning helices [7], [44] and corresponding regions could be found in the *Paramecium* CRCs via Kyte-Doolittle hydophobicity scales ([40]; see alignment in Figure 3). The predictions for transmembrane domains (TMDs) five and six, as well as TMD1 and TMD2 are highly reliable for all CRCs, whereas the positioning of TMD3 and TMD4 in CRC-I-2a, CRC-III-4c, CRC-IV-3, CRC-V-1, CRC-V-3, CRC-V-4, CRC-VI-2b and CRC-VI-3 channels varies between different algorithms and depends on the input queries, i.e. if full-length or C-terminal sections were analyzed. In all cases TMD3 and TMD4 are predicted to occur in close proximity within a continuous hydrophobic stretch covering both membrane helices.

The selectivity filter motif is conserved in metazoan InsP_3_ and ryanodine receptors, covering a six amino acid consensus sequence GGG^V^/_I_GD [45], and its role in ion permeability is supported by functional analyses of diverse InsP_3_ receptor mutants [45], [46]. Sequence analysis of the corresponding regions in the *Paramecium* CRCs reveals that three group IV members (CRC-IV-1a/b and CRC-IV-2) possess the complete six amino acid consensus sequence (Figure 3). In other CRCs conserved residues are restricted to a central core motif GG^V^/_I_G (CRC-I-1a/b/c, CRC-II-1a/b, CRC-II-2, CRC-II-3 and CRC-IV-3b) or to variants of it (CRC-I-2a/b, CRC-II-4, CRC-III channels, CRC-IV-3a, CRC-IV-4a/b, CRC-V channels), while CRC-VI channels show practically no similarity within this region. Therefore, the question remains open, whether the permeation properties of most of our CRCs comply with those known from metazoan InsP_3_ and ryanodine receptors, at least from the point of view of sequence analysis. However, regarding their selectivity for Ca^2+^, one should note that neither metazoan InsP_3_ nor ryanodine receptors are selective for Ca^2+^ [16], [47], [48] as both types are relatively non-selective large-conductance cation channels [47].

Evolutionary and functional constraints vary considerable for membrane proteins in different cellular compartments [49]. Transmembrane regions, however, diverge much more slowly than extramembrane regions [50]. We therefore used phylogenetic analyses to assess the allotment of the C-terminal transmembrane domains of *Paramecium* CRCs to corresponding sequences in metazoan InsP_3_ and ryanodine receptors, which share high sequence similarity (36% sequence identity) within this domain [44], thereby allowing a joint analysis of both receptor types. As shown in the neighbor joining tree (Figure 2B), the evolutionary relationships reveal that the channel domains of metazoan InsP_3_ and ryanodine receptors cluster together and that they are separate from the *Paramecium* sequences. Alternative evaluation by maximum parsimony (Figure S2) shows even more stringent relationships between the *Paramecium* CRCs which group separate from metazoan sequences. This, together with the occurrence of very similar domains in both, InsP_3_ and ryanodine receptors on the one hand and in individual CRCs on the other hand ([25], this paper), argues for a common ancestor.

The phylogenetic tree of Figure 2B also shows that the arrangement of the *Paramecium* CRCs in six different groups is consistent, as the results not only agree with previously published data [25] but also with data obtained from full-length analyses of the re-annotated sequences (Figure S3). An exception is one member of group VI channels, CRC-VI-3, whose N-terminal part is truncated. In full-length analyses CRC-VI-3 clusters with other CRC-VI channels, as these show slightest conservation among all CRCs and conserved parts are mainly restricted to their C-terminal channel domain. Phylogenetic analyses with the transmembrane domains reveal that CRC-VI-1 and the CRC-VI-2 subfamily cluster with the clade of group I, II and III channels, whereas CRC-VI-3 groups with CRC-IV and CRC-V channels.

Re-annotation of CRC-VI-3 unravels another unusual feature, as cDNA sequencing reveals four different transcripts, a phenomenon which has not been described to our knowledge for any other *Paramecium* gene so far [51]. As outlined in Figure 2C, continuous full-length transcripts result in an ORF of 6063 bp encoding a 2021 amino acid polypeptide. A comparison of the genomic sequence with their cDNA equivalent revealed six introns of 22 bp, 23, 25, 26, and 2×28 bp, all with canonical 5′ and 3′ exon-intron junctions (5′-GT. . .AG-3′) typical of *Paramecium* [51]–[53]. Strikingly, an intragenic region ‘x’ of 26 bp, also flanked by 5′-GT and AG-3′ nucleotides, was found to be spliced in several clones leading to a premature stop-codon. Two additional transcripts, also leading to premature stop-codons, result when introns 3 or 4 are not spliced.

### CRC-I channels

Group I channels has 5 members, from which subfamily 1 was selected, consisting of 3 closely related ohnologs, CRC-I-1a, CRC-I-1b and CRC-I-1c, with identities on the amino acid level of 94% (CRC-I-1a to CRC-I-1b) and 81% (CRC-I-1a/b to CRC-I-1c) (Table 1). The domain structure of CRC-I-1 channels is similar to the recently described CRC-II-1a channel [24] and is closely related to metazoan InsP_3_ receptors. Conserved regions cover the complete channel sequences (Figure 2A) including regions corresponding to the InsP_3_-binding domain of metazoan InsP_3_ receptors and, according to CRC-II-1a topology, a “central” RIH domain could be identified. “Central” means that only one RIH domain occurs, whereas metazoan InsP_3_ receptors possess two located in N-terminal and central parts of the proteins [14].

**Figure 4:**
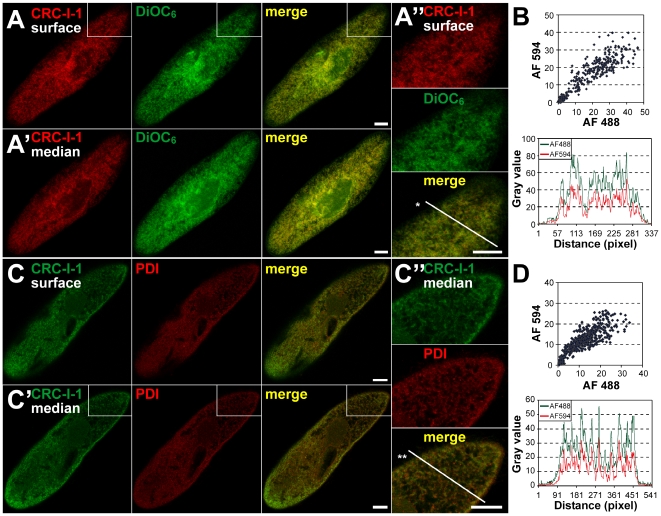
Localization of the CRC-I-1 subfamily in the ER. Surface (A) and median (A′) confocal slices of a cell stained with antibodies against CRC-I-1 (red channel) and the ER-marker DiOC_6_ (green channel). The tubular-reticular staining mostly overlaps in both channels (merge) indicating co-localization. (B) Co-localization also occurs when CRC-I-1 antibodies (green channel) where combined with antibodies against the ER-resident protein disulfide-isomerase PDI (red channel) in surface (C) and median (C′) views. Coincidence of the red and green channels in line scans as well as distributional profiles (*; **, B, D) through the enlargements (A″, C″) show considerable co-localization. Bars = 10 µm.

To localize CRC-I-1 channels, we raised polyclonal antibodies against a 108-residue polypeptide (G2220 to G2327 of CRC-I-1b; Figure S4A). This region shares 94.5% and 77.1% identity to the corresponding regions in CRC-I-1a and CRC-I-1c, suggesting that most likely the complete CRC-I-1 subfamily is recognized by the antibodies. The affinity-purified antibodies were tested in Western blots (Figure S4B) and further used in immuno-fluorescence analyses, where they reveal a strong staining of network-like structures throughout the cell (Figure S5A and S5B). Such staining pattern is characteristic of the endoplasmic reticulum (ER) and therefore we used different ER-markers in co-localization studies. CRC-I-1 staining mostly overlaps with DiOC_6_ (dihexyloxacarbocyanine iodide) labeling (Figure 4A), which is a marker for ER structures in *Paramecium* [54]. To objectify co-localization, we performed line-scan analyses and distributional profiles which yield a clear correlation between the two confocal channels (Figure 4B). Similar results were obtained when cells were double-stained with CRC-I-1 antibodies and a polyclonal mouse antibody against the ER-resident protein disulfide isomerase, PDI (Figure 4C and 4D). Therefore CRC-I-1 channels localize to the ER, as expected from mammalian InsP_3_ receptors [1], [55]. This has been confirmed by immunogold EM localization (Figure S6).

### CRC-III channels

The seven members of group III CRCs are less conserved than group I and II channels, because they lack the central RIH domain (Figure 2A). The alignment in Figure 3 reveals that the intraluminal loop between the pore region and TMD6 is altered in some CRC-III proteins, i.e. CRC-III-2, CRC-III-3 and CRC-III-4 channels. Compared to other CRCs, they have an additional insertion of variable length between the pore and the final transmembrane domain.

**Figure 5:**
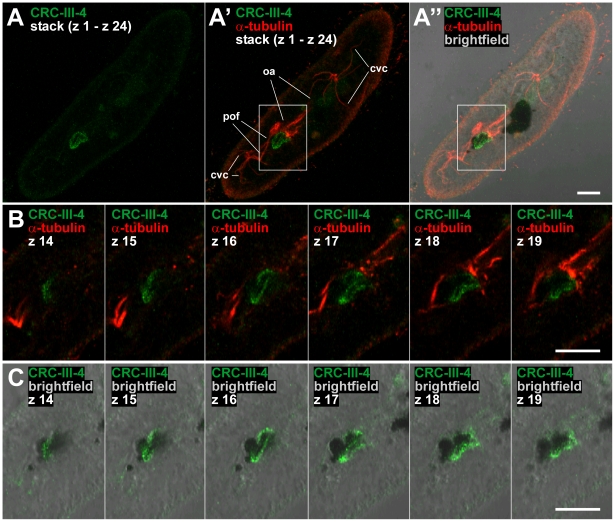
Antibodies against CRC-III-4 channels stain vesicles associated with α-tubulin and food vacuoles. (A) Confocal z-stack projection of a cell double-stained with antibodies against CRC-III-4 (green) and α-tubulin (red). (A′) CRC-III-4 labeling appears as speckled area close to the oral apparatus (oa) and the postoral fibers (pof). (A″) There is also a strong correlation with food vacuoles (black inclusions), which were visualized by feeding cells with Indian ink before fixation. (B) Confocal z-series of an enlarged detail from (A′) show dynamic arrangements of CRC-III-4 labeled vesicles, which surround a food vacuole (C) emerging from the oral apparatus. cvc = contractile vacuole complex. Bars = 10 µm.

**Figure 6:**
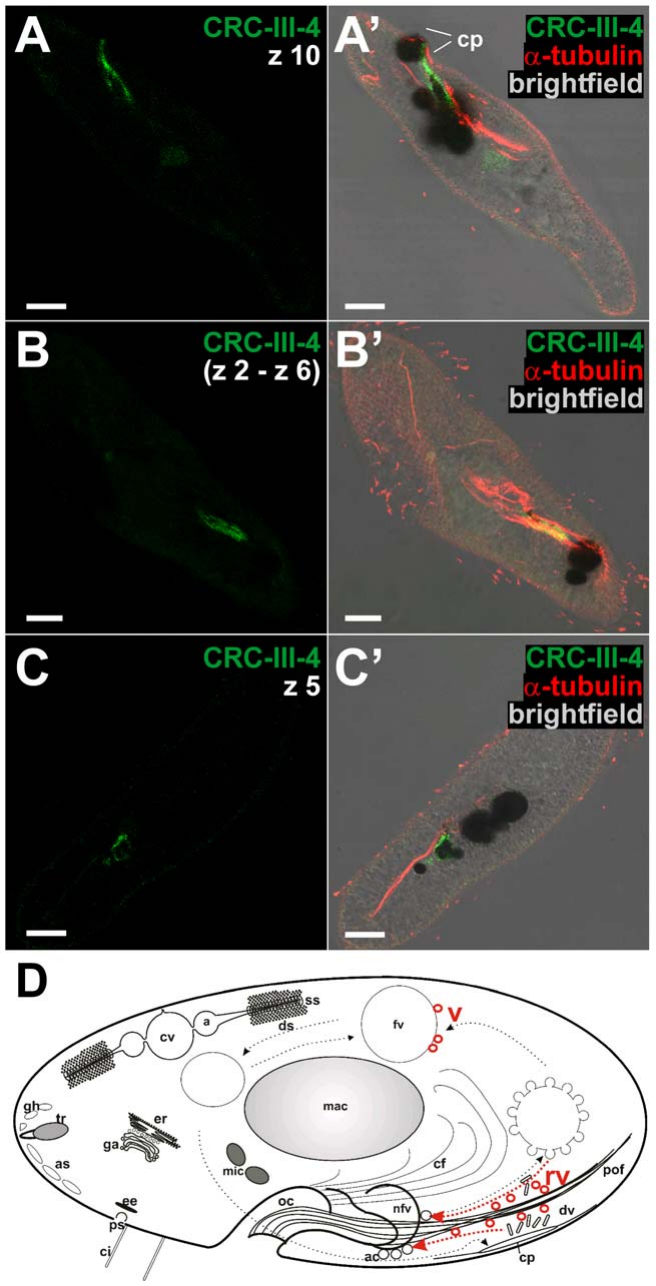
α-CRC-III-4 labeled vesicles traffic between the oral apparatus and the cytoproct. The mobility of CRC-III-4 labeled vesicles is shown by three examples of Indian ink-fed cells double-stained with antibodies against CRC-III-4 (green) and α-tubulin (red). (A) CRC-III-4 labeled vesicles are aligned along the postoral fibers and close to a food vacuole near the cytoproct (cp). Additionally, several labeled vesicles are visible on a food vacuole undergoing defecation at the cytoproct. (B) Intermediate arrangement of CRC-III-4 stained vesicles concentrated on microtubular bundles of the postoral fibers. (C) CRC-III-4 labeling of vesicles (v) in close proximity to, or on the membrane (not resolvable) of a food vacuole. (D) Scheme of the distribution of CRC-III-4 (red). For abbreviations see Figure 1. nfv = nascent food vacuole. Bars = 10 µm.

**Figure 7:**
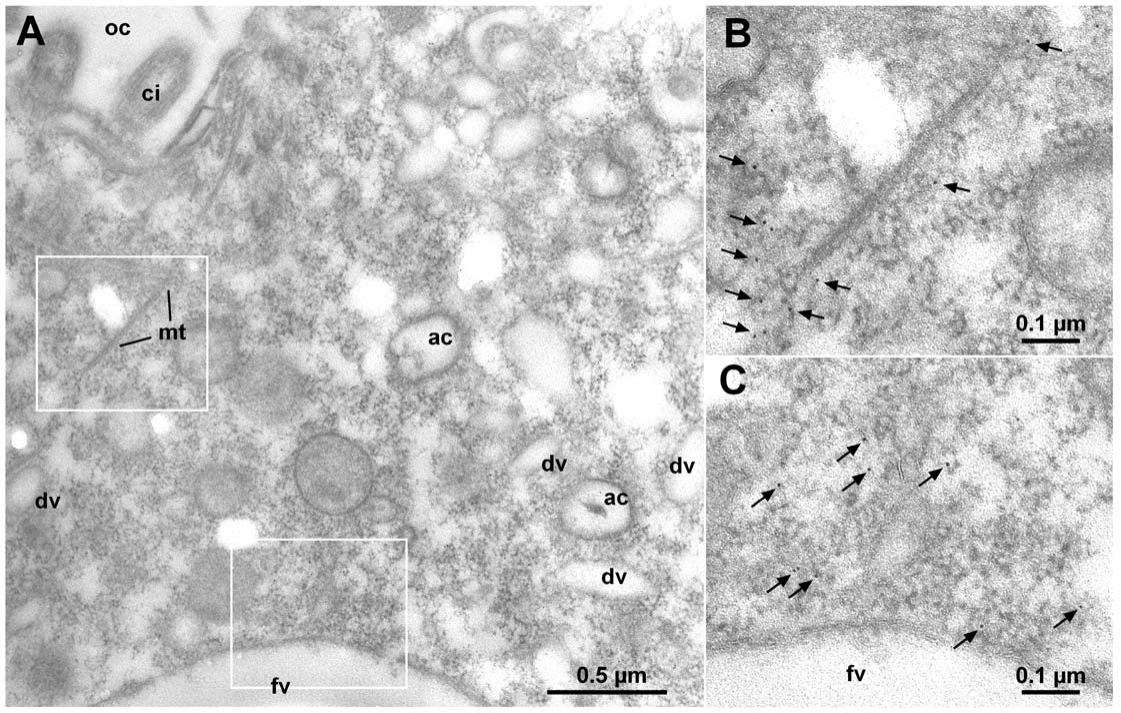
Immuno-gold EM localization of CRC-III-4. (A) EM micrograph showing a region between the oral cavity (oc, ci = cilium) and a newly formed food vacuole (fv). Immunogold labeling with antibodies against CRC-III-4 occurs at small vesicles in close proximity to microtubules (mt) (see enlarged detail in (B)) and the food vacuole (see enlarged detail in (C)). Other vesicles of the phagocytic cycle such as discoidal vesicles (dv) or acidosomes (ac) are not labeled.

We selected the CRC-III-4 subfamily and raised antibodies against a 136 amino acid polypeptide (L995 to Q1130 of CRC-III-4b; Figure S4A), a region which is highly conserved among CRC-III-4 channels (91% identity to CRC-III-4a and 82% to CRC-III-4c). The purified antibodies were used for Western blots (Figure S4C) and immuno-localization studies. Labeling with CRC-III-4-specific antibodies differed to some extent from cell to cell. It frequently appeared as speckled areas associated with microtubular ribbons emerging from the oral apparatus (Figures 5 and 6, see also Figures S5D, S5E, S5F, S5G, S5H). This suggests that the CRC-III-4 containing organelles are involved in the phagocytic cycle (see scheme, Figure 1). CRC-III-4 also localizes to phagosomes, which can be clearly identified in cells that have ingested Indian ink, in the vicinity of the cytopharynx where phagosomes form (Figure 5), to spent vacuoles disposing residual material at the cytoproct (Figure 6A) or to membranes of phagosomes undergoing cyclosis (Figure 6C). Booth, the variable positioning of CRC-III-4 labeling in different cells as well as its association with microtubular fibers (Figure 6B) suggests that the organelles containing CRC-III-4 represent phagosomes and recycling vesicles (Figure 6D). Immuno-EM localization (Figure 7) shows that CRC-III-4 labeling is associated with small, round vesicles along microtubules [56], whereas other vesicles contributing to the phagocytic cycle, such as discoidal vesicles or acidosomes were not labeled.

### CRC-V channels

These channels show a high degree of conservation regarding their C-terminal channel domain and they possess additionally conserved parts in N-terminal regions (Figure 2A). In three members, CRC-V-1, CRC-V-4a and CRC-V-4b, an RIH domain could be identified, which corresponds to the N-terminal RIH domain of metazoan InsP_3_ and ryanodine receptors (in contrast to the central RIH domain found in group I and II channels). As already observed with CRC-III channels, the intralumenal loop between the pore region and the TMD6 is elusive in CRC-V channels. In contrast to CRC-III channels, the distance between these regions is slightly shortened, leading to a shift of the putative selectivity filter region within the pore (Figure 3).

**Figure 8:**
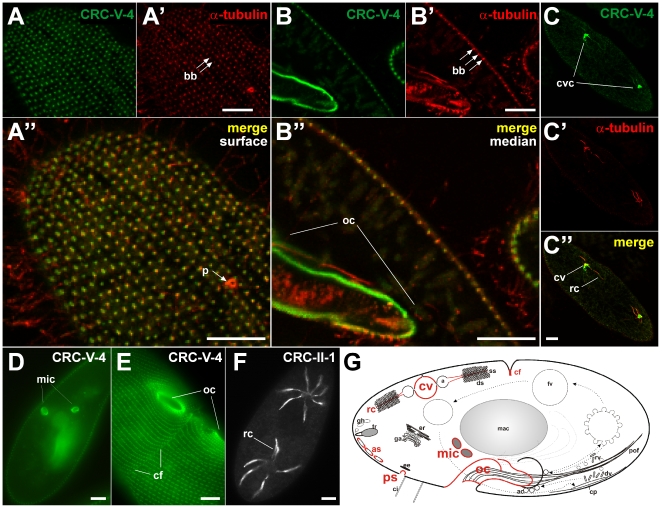
CRC-V-4 localize to multiple cellular targets. Double labeling of cells using antibodies against CRC-V-4 (green channel) and α-tubulin (red channel). Sections of surface (A) and median (B) confocal slices show CRC-V-4 labeling as punctate pattern along the cell cortex. The CRC-V-4 labeled organelles are positioned close to basal bodies (bb), but slightly shifted (A″), suggesting staining of parasomal sacs (ps). The median view (B) reveals a strong CRC-V-4 labeling along the oral cavity (oc). Additionally, CRC-V-4 labeling occurs at the contractile vacuole complex (cvc, C), the micronuclei (mic, D) and at the cleavage furrow (cf, E). The CRC-V-4 labeling is enriched on the contractile vacuole (cv), with only weak staining of the radial canals (rc), thereby coinciding with the microtubular scaffold of the contractile vacuole complex labeled with α-tubulin antibodies (C′, C″). The localization of CRC-V-4 is compared with CRC-II-1 channnels occurring in the smooth spongiome of the contractile vacuole complex (F, [24]). (G) Summary of CRC-V-4 distribution (see Figure 1 for abbreviations; cf = cleavage furrow, p = pore of the contractile vacuole complex). Bars = 10 µm.

We selected CRC-V-4 channels and raised subfamily-specific antibodies against a 129 amino acid polypeptide (S876 and Y1004 of CRC-V-4a; Figures S3A, S3D, and S4I, S4J, S4K, S4L, S4M). In immuno-fluorescence analyses, CRC-V-4-specific antibodies label a multitude of organelles (Figure 8), including cortical structures (Figure 8AB), the contractile vacuole complex (Figure 8C), the nuclear envelope of the micronuclei (Figure 8D) and, in dividing cells, the cleavage furrow (Figure 8E).

**Figure 9:**
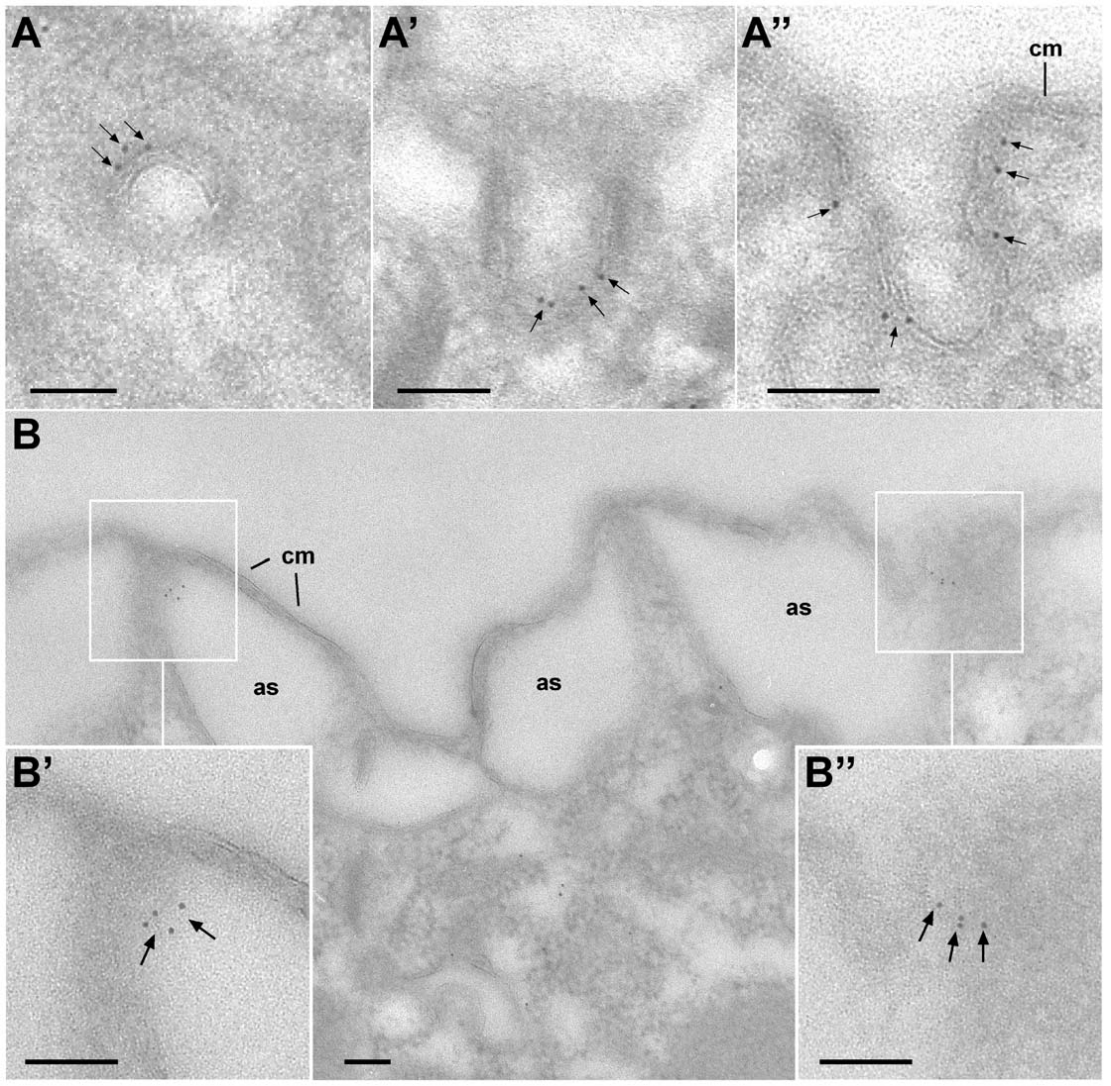
Cortical localization of CRC-V-4 channels at the ultrastructural level. Immuno-EM localization of CRC-V-1 reveals gold label (arrows) on parasomal sacs (coated pits) shown in cross (A), oblique (A′), and parallel section (A″). (B) A cortical region displaying alveolar sacs (as) attached to the cell membrane (cm) shows gold label (arrows) associated with contact sites between adjacent alveolar sacs (B, B′ and B″). Bars = 0.1 µm.

Staining with CRC-V-4-specific antibodies yields a regular, punctate pattern at the cell cortex (Figure 8A) in close proximity to basal bodies, although with a slight shift. This suggests that CRC-V-4 is present at the origin of the endocytotic route, i.e. at the multiple, regularly-spaced endocytotic sites, called parasomal sacs ([57], see Figure 1A and 1B). CRC-V-4 fluorescence is particularly abundant in uppermost regions of the oral apparatus (Figure 8B), the cytostome, where these organelles are even more concentrated [57]. In immuno-gold EM analyses, the CRC-V-4 labeled structures could be clearly identified as parasomal sacs (Figure 9A). Additionally, some CRC-V-4 labeling occurs at the contact sites of adjacent alveolar sacs, representing cortical Ca^2+^ stores (Figure 9B, see Figure 1C). Note that another CRC type, CRC-IV-1, was previously shown to be associated with the outer side of alveolar sacs [25]. This zonal arrangement of different CRCs within one organelle suggests amplification and/or functional diversification for both channels types.

A similar observation was made with the contractile vacuole complex. An involvement of this organelle in Ca^2+^-homeostasis is assumed [32]. Previous studies revealed an association of CRC-II-1 channels with the smooth spongiome (Figure 8F), a brush-like network of tubular membrane extensions of the contractile vacuole complex [24]. Now we see CRC-V-4 also in the contractile vacuole complex, but the fluorescence signal is most abundant on the contractile vacuole itself, with only slight staining beyond, e.g. along the radial canals (Figure 8C). Therefore, the CVC harbors two types of CRCs, also with a zonal arrangement within one organelle.

### CRC-VI channels

**Figure 10:**
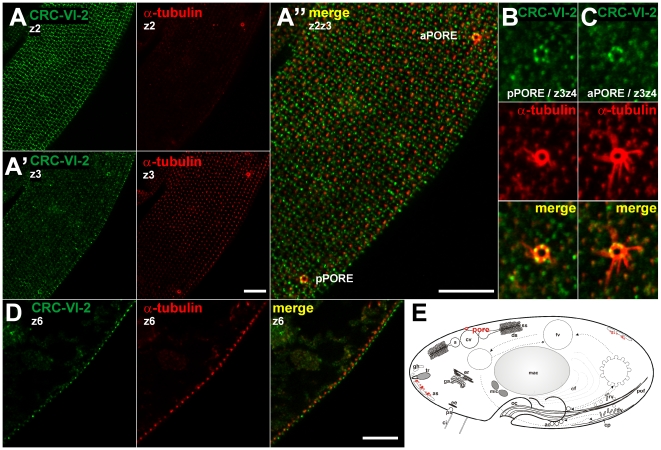
CRC-VI-2 channels occur near the intersections of longitudinal and perpendicular ridges along the surface fields (kinetids). Superficial confocal slices of cells double-stained with antibodies against CRC-VI-2 (green) and α-tubulin (red). The network-like labeling appears close to the cell surface at a level above basal bodies, which were stained with α-tubulin antibodies (compare z2 and z3 slices shown in A and A′, respectively). As shown in the enlargement (A″), CRC-VI-2 labeled structures are located between adjacent basal bodies and enriched along the intersections of longitudinal and perpendicular ridges. The anterior pore (aPORE) and the posterior pore (pPORE) of the contractile vacuole complex (enlargements in [B] and [C]) display a dotted labeling. A median view (D) shows that CRC-VI-2 label is located at a level slightly above basal bodies (see Figure 11). (E) Schematic localization of CRC-VI-3 (red, for abbreviations see Figure 1A) includes data from EM studies (Figure 11) which identifies cortical vesicles as the equivalent of labeling near the cell surface. Bars = 10 µm.

**Figure 11:**
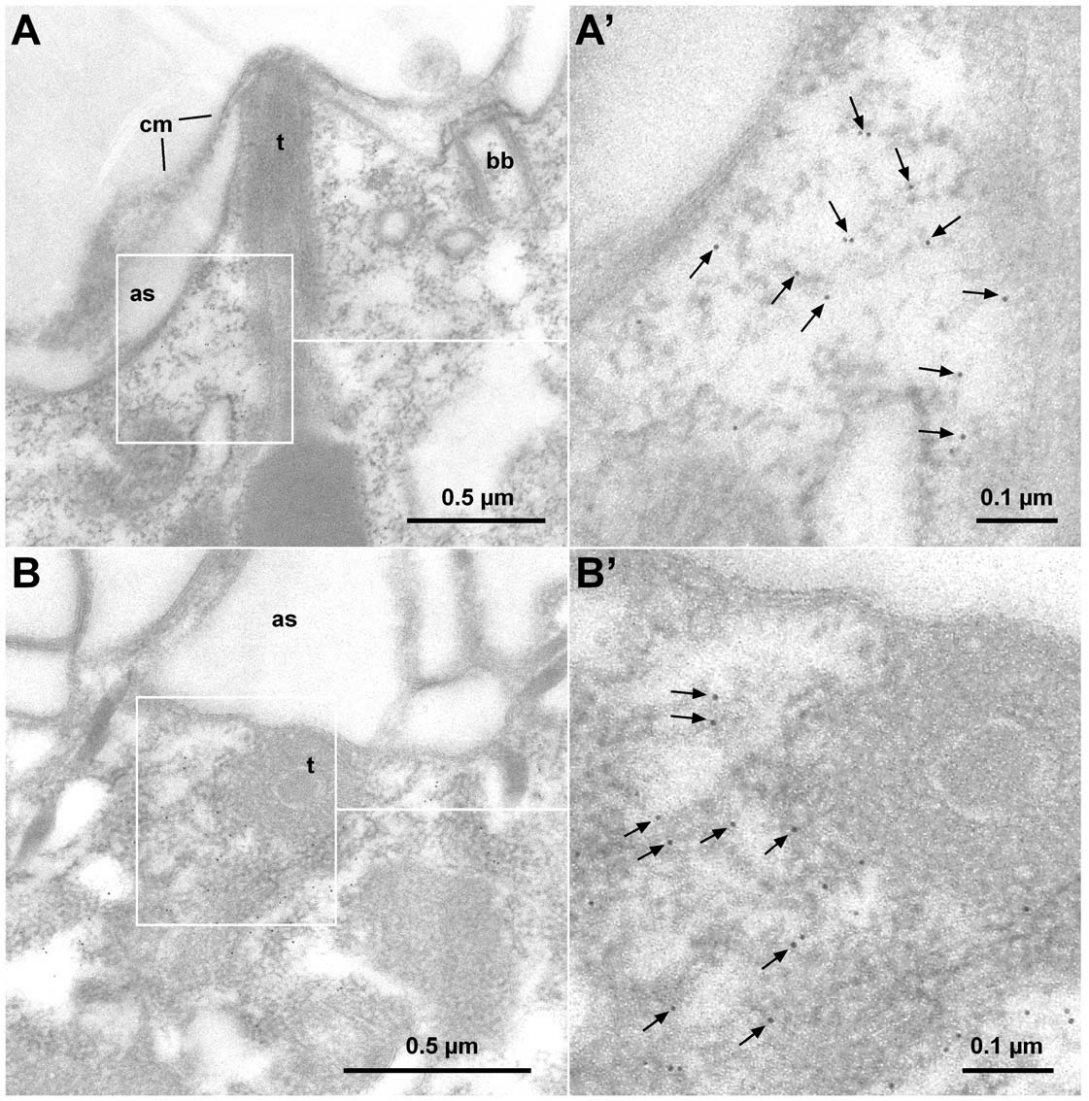
Immunogold EM localization of CRC-VI-2. In immuno-gold EM analyses, CRC-VI-2 antibodies label a population of small vesicles, which are below alveolar sacs (as) and in close proximity to trichocysts (t). This is shown in a longitudinal (A) and a cross-section (B). Enlarged details (A′, B′) are from boxed areas.

These are the most aberrant channels as conserved parts are restricted to their C-terminal channel domains, thereby resembling CRC-IV channels. In order to localize subfamily CRC-VI-2, we raised antibodies against a CRC-VI-2a-specific polypeptide (I1133 to N1229; Figure S4A) and used them in affinity-purified form for Western blots and immuno-fluorescence analyses (Figures S4E and S5N, S5O, S5P). A cortical framework was labeled, which is particularly intense along the longitudinal and perpendicular ridges of the cell surface (Figure 10). This pattern can be attributed to the close apposition of the organelles containing CRC-VI-2 to the plasma membrane, as they are definitely arranged along cell surface ridges, i.e. above the level occupied by basal bodies (Figure 10A and 10D). This becomes clearer from immunogold-EM analyses, where gold-label was enriched on vesicles of unknown specificity, located in close proximity of trichocyst tips and the alveolar sacs (Figure 11). CRC-VI-2 channels are also localized to the pores of the contractile vacuole complex (Figure 10B and 10C), where reversible fusion and fission events of the vacuole membrane with the cell membrane take place. CRC-VI-2 may provide Ca^2+^ to support such a function.

**Figure 12:**
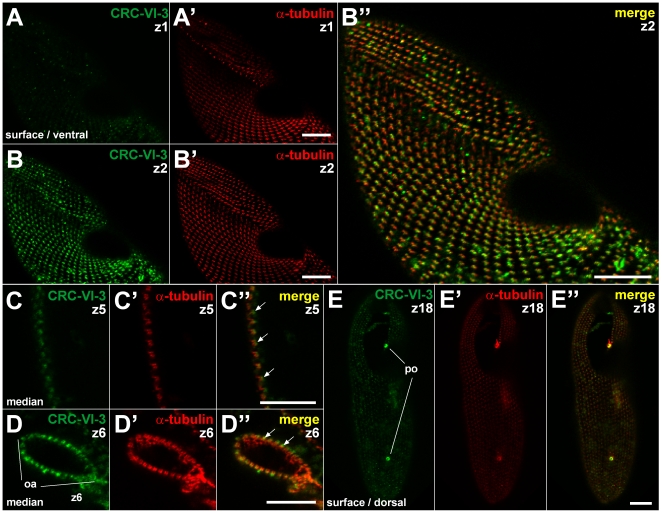
CRC-VI-3 localize to early endosomes. Double labeling of cells using antibodies against CRC-VI-3 (green) and α-tubulin (red). CRC-VI-3 specific antibodies recognize a punctate pattern at the cell cortex in close proximity to, but slightly below the basal bodies which were stained with α-tubulin antibodies (compare z1 slices shown in A and A′ with z2 slices in B and B′; for merge see B″). The positioning of the CRC-VI-3 labeled organelles below basal bodies (arrows) is also shown in median sections (C, C′, C″) as well as in cross-sections of the oral apparatus (oa; D, D′, D″). All this demonstrates localization of CRC-VI-3 to early endosomes. Moreover, in a dorsal view of the cell CRC-VI-3 is present at the pores (po) of both contractile vacuole complexes (E, E′, E″). Bars = 10 µm.

**Figure 13:**
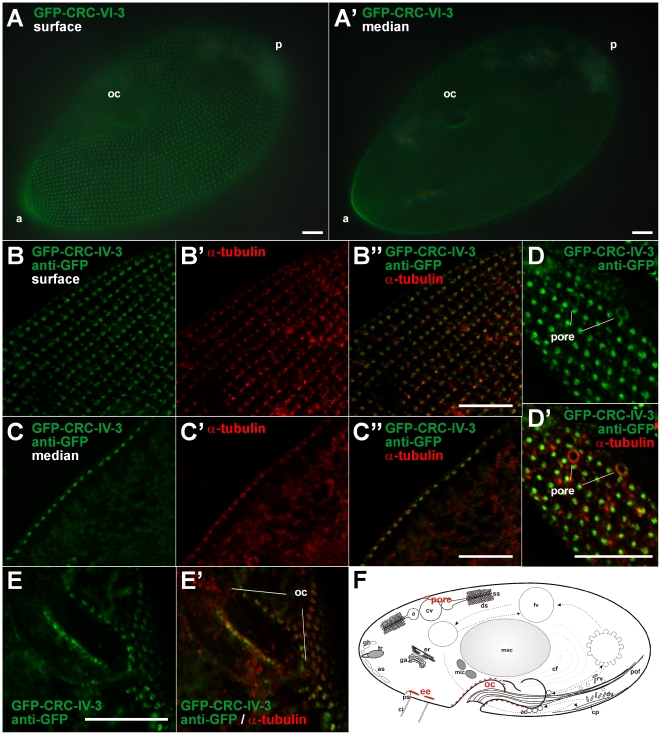
GFP-localization of CRC-VI-3 to early endosomes. Surface (A) and median (A′) view of a live cell expressing a GFP-CRC-VI-3 fusion protein arranged in a dotted cortical fluorescence pattern. (B to E) Sections of confocal image slices of fixed GFP-CRC-VI-3-expressing cells double-stained with antibodies against GFP (green) and α-tubulin (red). The superficial slices (B – B″) show GFP-staining of dot-like structures next to basal bodies. The median slices (C – C″) reveal that these dots are slightly below basal bodies, suggesting that the labeled organelles represent terminal cisternae (early endosomes) and/or associated vesicles (not resolvable). In a superficial slice, dorsal parts of the cell show GFP-labeling at the pores of the contractile vacuoles (D, D′). (E, E′) Cross-sections of the oral cavity (oc) with punctate GFP-labeling close to basal bodies in the cytostome. (F) Scheme of a *Paramecium* cell superimposed with CRC-VI-3 distribution (red). For abbreviations see Figure 1A. Bars = 10 µm.

The CRC-VI-3 protein is exceptional because N-terminal parts of this channel type are truncated (Figure 2A and 2C). We therefore localized this channel independently by immuno-staining (Figure 12) and by expression as a GFP-fusion protein (Figure 13). We raised antibodies against a 102 amino acid polypetide (T579 and L680 of CRC-VI-3; Figure S4A and S4F). This region overlaps with the genomic region covering introns 3 and 4, which were shown to be aberrantly spliced (Figure 2C). Labeling with these antibodies yields a regular punctate pattern at the cell cortex and the oral apparatus (Figure 12A–12D) as well as labeling of the pores of the contractile vacuole complex (Figure 12E). Double-staining of CRC-VI-3 and α-tubulin shows that CRC-VI-3 is present in an organelle below basal bodies. Cells expressing a GFP-CRC-VI-3 fusion protein yield a similar fluorescence pattern (Figure 13A). In transformed cells, which were double stained with anti-tubulin antibodies and anti-GFP antibodies to enhance GFP-fluorescence, CRC-VI-3 is again associated with an organelle slightly below basal bodies (Figure 13B and 13C). Labeling of the pores of the contractile vacuole complex (Figure 13D) by the two methods is also compatible.

The positioning of the CRC-VI-3 containing compartments below ciliary basal bodies identifies them as ‘terminal cisternae’, which correspond to early endosomes ([57], see Figure 1B), and associated vesicles, as supported by immuno-gold EM analysis (data not shown). This compartment arises from parasomal sacs (and possible additional input), where CRC-V-4 channels occur (Figures 8 and 9) and both compartments are connected via trafficking of “pre-endosomal” vesicles [57]. Again this localization points to an involvement of CRC-VI-3 in membrane to membrane interaction.

## Discussion

### Criteria for identification

The 34 *Paramecium* CRCs were identified by a homology based database search with conserved domains of metazoan InsP_3_ and ryanodine receptors. Retrieved sequences were authenticated by performing a re-BLAST against the databases available at NCBI [25]. Conserved parts of the 34 CRCs are most pronounced in regions of the C-terminal channel domain (Table 1). Different prediction methods resulted in a six membrane spanning topology, with the pore region within TMD5 and TMD6, thereby resembling channel domains of known InsP_3_ receptors [16]. Their relationship to this channel type is also endorsed by the size of the *Paramecium* CRCs, as none of them reaches the size of metazoan ryanodine receptors, i.e. ∼500 kDa. However, only 16 CRCs possess regions which align across the InsP_3_-binding region located at the N-terminal side, suggesting that at least these types might be activated by InsP_3_. In fact, we have identified one of them (CRC-II-1) by InsP_3_-binding and InsP_3_ uncaging effects as a genuine InsP_3_ receptor [24]. In other CRCs the nature of putative agonists is questionable particularly since from sequence analyses these CRC types could not be assigned to the ryanodine receptor type. Nevertheless, for one subfamily, CRC-IV-1, we could recently demonstrate that these types are ryanodine receptor-related channels, as they possess similar pharmacological properties. Considering the fact, that CRC-IV-1 channels respond to the ryanodine receptor agonists 4-CmC and caffeine, in despite of insensitivity to ryanodine, we have deduced a distant relationship, thereby classifying them as a novel mixed-type of CRCs [25].

### Differential localization

**Figure 14:**
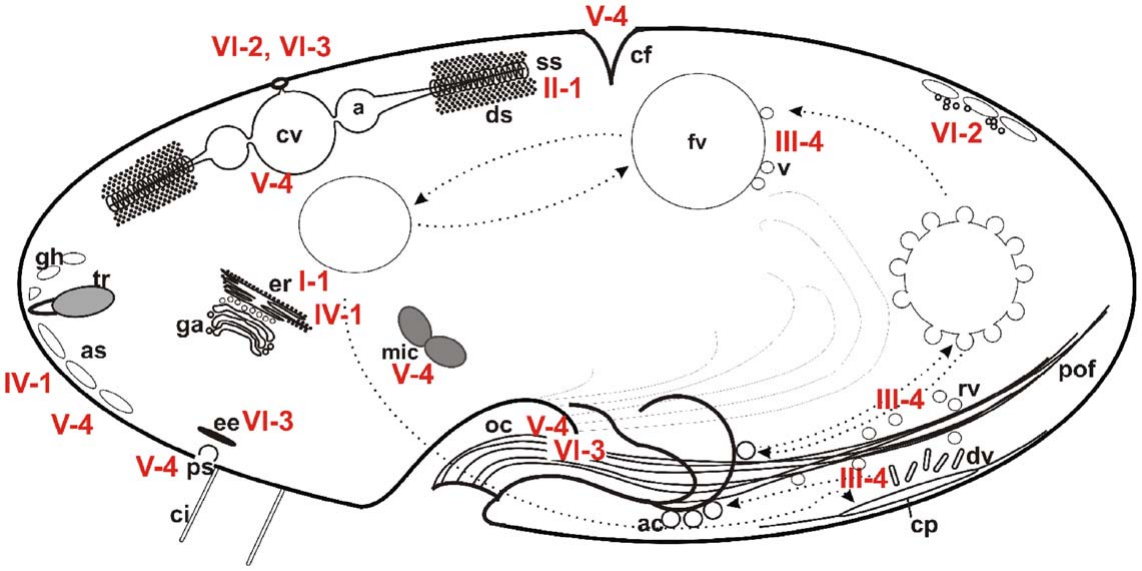
Summary of CRC localization studies revealing a widely different and distinct distribution of CRC channels. Cartoon of a *Paramecium* cell superimposed with the distribution of the CRCs (red), based mostly on the present study, but also considering data from previous work [24], [25]. Two channel types, CRC-I-1 and CRC-IV-1, are located in the endoplasmic reticulum (er). CRC-IV-1 channels also occur in the outer membranes of alveolar sacs (as), where we additionally found CRC-V-4 channels enriched at the contact sites between neighboring alveolar sacs. Furthermore, CRC-V-4 channels are found on parasomal sacs (ps), the oral cavity (oc), the micronuclei (mic) and, in dividing cells, at the cleavage furrow (cf). Furthermore, CRC-V-4 channels localize to the contractile vacuoles (cv) and the radial canals, both elements of the contractile vacuole complex (cvc), where additionally CRC-II-1 channels occur in membranes of the smooth spongiome (ss). CRC-III-4 channels are found on recycling vesicles along the postoral fibers (pof), located between the oral cavity (oc), around some food vacuoles (fv) and at the cytoproct (cp). CRC-VI-2 channels are localized to cortical vesicles enriched near alveolar sacs and trichocyst (tr) tips. CRC-VI-3 is associated with early endosomes (ee). Both these channel types were also found at the pore of the contractile vacuole complex.

The focus of this work is on the identification and localization of CRCs. Those analyzed in this study reveal highly specific targeting to a variety of cellular compartments. As Figure 14 summarizes, including previous work on CRC-II-1 [24] and CRC-IV-1 [25], this holds true of the ER (CRC-I-1, CRC-IV-1, CRC-VI-2), the contractile vacuole complex (CRC-II-1, CRC-V-4), the alveolar sacs (CRC-IV-1, CRC-V-4), vesicles of the phagocytotic (CRC-III-4) and the endocytotic (CRC-V-4, CRC-VI-3) cycle as well as the micronuclear envelope and the cleavage furrow (CRC-V-4). Their differential localization implies functional involvement in membrane trafficking (CRC-I-1, CRC-III-1, CRC-IV-1, CRC-V-4, CRC-VI-2 and CRC-VI-3), Ca^2+^ homeostasis (CRC-II-1) and cellular morphogenesis (CRC-I-1) as observed in pilot experiments by gene silencing (data not shown). The high diversification even within organelles might be prerequisite to spatial and temporal versatility of Ca^2+^ signaling within specialized microdomains.

### Why so many CRCs and why at so many different sites?

This question has evolutionary as well as functional implications. In evolutionary terms, diversification within the groups is due to whole genome duplications with formation of ohnologs, as generally assumed for *Paramecium* [58]. This is supported by the respective similarities shown in Table 1. Functionally the distinct and mostly mutually exclusive localization of the different channel groups I to VI may reflect different channel properties which may be relevant for local Ca^2+^-release and signaling. For a channel function one has to assume Ca^2+^ storage in the respective organelles. Given the paucity of information in ciliated protozoa, we have to consider knowledge from higher eukaryotes, i.e. mainly mammals, where Ca^2+^ storing organelles are much better known and where Ca^2+^ is known to regulate specific vesicle trafficking steps. In *Paramecium*, luminal enrichment of Ca^2+^ has been ascertained only for the contractile vacuole complex [32] and for alveolar sacs [27], [28]. In these organelles we have previously localized CRCs type II and IV, respectively [24], [25]. All other CRC or CRC-like channel types described here are new. From the presence of a SERCA-type pump in the ER [59] one can also consider the ER as a Ca^2+^ store - in agreement with the current finding of channels type CRC-I-1 and CRC-IV-1 in this compartment.

Channels type III-4 are associated with elements of the phagocytic pathway, including vesicles recycling from mature phagosomes (food vacuoles) and from the cytoproct to the nascent food vacuole, as described by Allen and Fok, 2000 [26]. Antibodies also label parts of some progressed stages of the food vacuole, but it could not be established whether this is due to associated vesicles of which several types fuse and pinch off during cyclosis [26], [60]. All this is compatible with knowledge from mammalian cells where formation of nascent phagosomes requires a local Ca^2+^ supply [61], [62].

The localization of channels type CRC-V-4 and CRC-VI is much more intriguing since antibodies bind to widely different, though distinct targets. Most provocative is the occurrence of CRC-V-4, endowed with a putative InsP_3_-binding domain, in parasomal sacs. Work with B lymphocytes could ascertain the presence of only ∼2 InsP_3_ receptors in the cell membrane [63]. Similarly a small number of ryanodine receptors can reach the cell membrane [64]. While the functional significance is not quite clear in any system, it demonstrates occurrence of one channel type, represented by the two closely related ohnologs CRC-V-4a and CRC-V-4b (94% identity), in widely different membranes [63]. The immuno-gold labeling density we see with CRC-V-4 in the EM by far outnumbers any expectation, alone on one parasomal sac (Fig. 9A, A′, A″). As to be expected, the gold label is clearly on the cytosolic side and such channels could – quite unexpectedly – mediate Ca^2+^ influx. It may be a candidate accounting for leakage conductances which so far hardly have been analyzed in *Paramecium* [65]. Functionally this resembles non-selective, high conductance, membrane potential-independent cation channels in hippocampal neurons which are activated by stepwise increase of extracellular [Ca^2+^] [66]. Strikingly, under such conditions, fluorochrome analysis in *Paramecium* revealed rapid increase of intracellular [Ca^2+^] [67]. To ascertain such a function for CRC-V-4, this channel now is amenable to electrophysiology.

Another intriguing aspect is the occurrence of CRC-V-4 channels in the contractile vacuole complex (vacuole and emanating radial canals) and on the flanks of alveolar sacs, because these organelles are known to contain also CRC-II-1 and CRC-IV-1 type channels, respectively [24], [25]. Here, CRC-V-4 channels types may serve functions different from those previously assigned to these organelles, i.e. fine-tuning of [Ca^2+^]_i_ homeostasis and Ca^2+^ supply for exocytosis, respectively. Alternatively, a double set of CRCs might enhance the potential for Ca^2+^ mobilization or also serve functional diversification [68]. Labeling with anti-CRC-V-4 antibodies in uppermost regions of the oral cavity (cytostome) is compatible with the occurrence of coated pits and of alveolar sacs in this region. The cell surface pattern, which contains such components in regular arrangement, is subject to epigenetic control and, thus, may require local constitutive Ca^2+^ signals, as suggested by Prajer et al. 1997 [69].

Strikingly CRC type V-4, which is different from CRCs in the ER, is associated only with the generative micronucleus. As we could not observe it in the transcriptionally active macronucleus, it cannot be engaged in transcriptional activity, but may serve any of the manifold functions discussed for nuclear CRCs in mammalian cells [70]. Occurrence of CRC-V-4 channels in the cleavage furrow appears feasible considering participation of several types of vesicles [71] and requirement of a Ca^2+^ signal [72], [73] for cytokinesis, as known from higher eukaryotes.

Also intriguing is the distribution of type VI CRCs. Taking into account their considerable diversity, two different subfamilies have been analyzed. We find CRCs of subtype VI-2 in cortical vesicles located below alveolar sacs and associated with the pore of the contractile vacuole complex. Not all of the small cortical vesicles seen in the EM have been categorized so far in terms of identity and trafficking. EM data obtained with *Paramecium* by silencing of the SNARE specific chaperone, NSF [74], [75], suggests that small vesicles may have access to the cell membrane not only via the orthodox route (via parasomal sacs which are considered for endocytosis and intermittent constitutive exocytosis [76]), but also along other sites of the cell surface. A detailed analysis is now required. Antibodies against both, CRC-VI-2 and occasionally CRC-VI-3 label the pore of the contractile vacuole complex. Thus, this site of cyclic exocytosis seems to be regulated by local Ca^2+^ signals, although the underlying small Ca^2+^ storage compartment remains to be identified. As to CRC-VI-3, its localization of to early endosomes and to the oral cavity is in agreement with the endocytic activity in superficial and oral regions of the cell.

May therefore a common trafficking route underly the distribution pattern of CRCs in *Paramecium* and higher eukaryotes cells? Ca^2+^ is assumed to decrease rapidly after formation of an early endosome and to increase only toward late endosomes and lysosomes [36]. Other analyses also report Ca^2+^ in recycling endosomes [77]. Formation of phagosomes generally involves Ca^2+^, as does their fusion with lysosomes [78]. Furthermore, homotypic vacuole fusion requires release of Ca^2+^ from the organelles [79]. All this supports our finding of CRCs in vesicles known to undergo endocytosis and recycling. In higher organisms, not all of the channels involved are identified and also in *Paramecium* a thorough characterization is a goal for future work.

### Are there cues to differential targeting?

From the topology of CRC-type molecules one may reasonably assume delivery to target membranes via membrane trafficking. This has been established, e.g. for ryanodine receptors in sarcoplasmic reticulum [80] and InsP_3_ receptors [81], [82]. Therefore, one may look for targeting cues in *Paramecium* on the basis of the differential localization of SNARE proteins, such as syntaxins, Syx [83], and synaptobrevins, Syb [75], [84].

In the cell membrane, no CRCs occur outside parasomal sacs (CRC-V-4). The terminal cisternae (early endosomes), however, contain CRC-VI-3. A similar discrepancy occurs with Syx1 which is spread over the entire cell surface and absent from terminal cisternae; only Syb6 occurs in parasomal sacs and in terminal cisternae (in addition to Syb11). Thus, the distribution of CRCs is in part different from, and in part identical to that of SNAREs. However, when parasomal sacs and early endosomes, both occurring in the cell cortex as well as in the cytostome, are compared, both these domains contain the same SNAREs (with some additional ones in the oral cavity) and both also contain CRC-V-4 and CRC-VI-3. In sum, SNAREs could theoretically mediate some of the membrane specific delivery of CRCs in *Paramecium*.

We also had a closer look at any coincidence with organelle specific Rab proteins, on the basis of an analysis in *T. thermophila* [85]. However, no such correlation could be found. In sum, the distribution of organelle/membrane specific CRCs may be guided, to some extent, by specific SNAREs. In the end, membrane specific targeting of CRCs may depend on cooperative factors and, thus, require much more detailed analysis in the future. With this respect the situation may be similar to the mutually interdependent targeted delivery of SNAREs, H^+^-ATPase subunits and actin [60]. Beyond this, different CRCs can coexist in one organelle also in mammalian cells, e.g. InsP_3_ and ryanodine receptors [86] or different InsP_3_ receptor subtypes [68]. Along these lines, the coexistence of CRCs type IV-1 and V-4 in alveolar sacs and of type I-1 and IV-1 in the ER reminds localization of InsP_3_ and ryanodine receptors in the sarcoplasmic reticulum of smooth muscle [87] and in the ER of sensory neurons [88].

### Differential elimination – on the way to pseudogene?

The *Paramecium* genome is intron-rich (average 2.3 per gene, [58]). A typical feature of these introns is their small size, with an average length of 25 nucleotides, and canonical GTA 5′- and TAG 3′-borders. They are spliced via intron definition [51] as it is known from small introns of other unicellular eukaryotes [89]. Remarkably, alternative splicing apparently does not occur, as there is no evidence for exon-skipping [51]. Analysis of EST sequences reveals the scarce use of closely spaced 3′ alternative splice sites in very few transcripts (frequency below 0.9%, [51]). The authors also show that mis-spliced or unspliced RNAs containing premature stop-codons are removed via the nonsense-mediated mRNA decay pathway [90] to prevent translation of such RNA molecules.

For two of the *Paramecium* CRC genes, CRC-III-1bΨ and CRC-VI-3, cDNA analysis reveals transcripts resulting in premature stop codons. In case of CRC-III-1bΨ no continuous ‘full-length’ transcripts could be detected, indicating that this gene has become a pseudogene. This was shown for several *Paramecium* genes so far (*Ptact1-10*, [91]; *Ptsyx6-2* and *Ptsyx13*, [83]; *Ptcen_icl3c*, [92]; *PtVAMP741*, [75]; *PTMB.411c*, [93]). The number of pseudogenes present in the *Paramecium*DB was estimated as ∼3.75% of all genes [58]. The peculiarity of the CRC-III-1bΨ pseudogene is that the nonsense transcripts result from an insertion mutation (indel) in the macronuclear DNA and not from mis-splicing, as flanking introns are spliced in the same way as introns of the corresponding ohnolog, CRC-III-1a (compare Figures S1D and S1E).

The situation described for CRC-VI-3 is also new, as we could detect i) transcripts resulting in pre-mature stop-codons as well as continuous ‘full-length’ transcripts and ii) the presence of an intragenic region with features of a typical *Paramecium* intron which was found to be aberrantly spliced (Figure 1C). In sum, this subset of CRC-VI-3 transcripts producing in-frame stop codons might represent a way to pseudogene formation.

### Conclusions

The 34 CRCs we find in the *Paramecium* cell can be attributed to several whole genome duplications, the last one resulting in closely related ohnologs [58]. We could not analyze all of them differentially since most antibodies were generated against subfamily-specific sequences (22 subfamilies in 6 groups). However, alone the presence of this large number of subfamilies/groups, each with differential localization, indicates wide diversification of CRCs in this cell. We also see secondary reduction by pseudogenization, whereby aberrant splicing may play a role. Most of the *Paramecium* CRCs share properties of both, canonical ryanodine and InsP_3_ receptors, e.g. considering the number of transmembrane domains and presence/absence of a putative InsP_3_-binding domain. (Two of the CRCs have been identified as ryanodine-related and InsP_3_ receptors, respectively, also according to functional criteria). The occurrence of such mixed character may represent an evolutionary stage closely related to a common ancestor molecule. CRCs of this kind should now be amenable to identification on a molecular basis also in other protozoa, where functional analyses with putative activators clearly suggest their occurrence. While we have focused in this paper on InsP_3_ and ryanodine type CRCs, one has to consider that a *Paramecium* cell is endowed with a multitude of other channels [65].

## Materials and Methods

### Paramecium cultures

Experiments were carried out with *P. tetraurelia* wild-type stocks 7S and d4-2, both derived from stock 51S [94]. Cells were cultivated in a decoction of dried lettuce, supplemented with 0.4 µg/ml β-sitosterol at 25°C, which was monoxenically inoculated with *Enterobacter aerogenes* as a food organism. Alternatively, cells were grown axenically in a sterile medium [95].

### Computational analyses

BLAST searches were performed according to Altschul [96] by using either the NCBI database (http://blast.ncbi.nlm.nih.gov) or the *Paramecium* genome database (http://paramecium.cgm.cnrs-gif.fr). Membrane topologies were predicted using the TOPCONS consensus prediction server ([38]; http://topcons.net/), the TMHMM server v. 2.0 ([39]; http://www.cbs.dtu.dk/services/TMHMM/) and Kyte Doolitte hydrophobicity scales [40] integrated in the DNASTAR® Lasergene® 7.2 software (DNASTAR, Madison, WI). Protein alignments were performed with CLUSTAL W [97] and further processed using the BoxShade surfer (http://www.ch.embnet.org/software/BOX_form.html). Phylogenetic analyses were carried out using MEGA version 3.0 [98] generated with neighbor-joining or maximum parsimony tree-building algorithms [99] with 1000 bootstrap replicates run. For analysis of transmembrane domains, proteins were aligned by CLUSTAL W (see above) and sequences were manually trimmed by removing N- and C-terminal variable regions from the multiple alignment before the aligned sites were used as input for MEGA 3.1.

### Determination of ORFs of CRC sequences

The open reading frames (ORFs) of CRC channels were identified by amplifying mRNA sequences by reverse transcriptase PCR (RT-PCR). Total RNA was extracted using the peqGOLD Trifast DNA/RNA/protein Purification System (Peqlab, Erlangen, Germany) or with the RNeasy Micro Kit (Qiagen, Hilden, Germany) according to the manufacturers' protocol. For amplifying intragenic RNA sections, cDNA synthesis was carried out with the Omniscript RT Kit (Qiagen). To determine 3′-ends of transcripts, RNA samples were treated with an additional DNase I digestion step and the reverse transcription reaction was performed using the PowerScript reverse transcriptase (Bioline, Luckenwalde, Germany) in the presence of 0.5 µM of a 3′-anchored dT-primer (Dei-1; for primer sequences see Table S2). PCR-amplification of cDNAs was performed with the Advantage 2 PCR Enzyme System (Clontech, Palo Alto, CA), the iProof High-Fidelity DNA Polymerase (Bio-Rad, Munich, Germany) (for primer sequences see Table S2) or the Phusion® High-Fidelity DNA Polymerase (Finzymes, Vantaa, Finland). Reactions were carried out in 35 cycles of 95°C for 30 s, 53°C for 20 s and 68°C for 120 s (Advantage 2 PCR Enzyme System) or in 35 cycles of 98°C for 10 s, 58°C for 15 s and 72°C for 60 s (iProof High-Fidelity DNA Polymerase and Phusion® High-Fidelity DNA Polymerase). PCR-amplified macronuclear DNA and cDNA fragments were cloned in the pCRII-TOPO or the Zero Blunt TOPO cloning system (Invitrogen, Carlsbad, CA) and analyzed by sequencing (Eurofins MWG Operon, Martinsried, Germany).

The cDNAs of CRC-VI-3 were prepared from two different RNA isolates derived either from axenically or monoxenically grown cells. PCR reactions were performed with two different polymerases (see above). Regions covering introns 3, 4 and the intragenic region ‘x’ (Figure 2B) were amplified using different PCR primers (Table S2).

### Recombinant expression of CRC-specific peptides

To obtain subfamily-specific antibodies, we expressed CRC-I-1b-, CRC-III-4b-, CRC-V-4a-, CRC-VI-2a- and CRC-VI-3-specific peptides by selecting antigenic regions with less than 35% identity between subfamilies within one group and less than 20% identity to members of other groups. To ensure that each antibody recognizes all members of one subfamily, the identities were more than 85% within the subfamilies. After mutating the deviating *Paramecium* glutamine codons (TAA and TAG) into the universal glutamine codons (CAA and CAG) by PCR methods [100], the coding regions of either G2220 to G2327 of CRC-I-1b, L995 to Q1130 of CRC-III-4b, S876 to Y1004 of CRC-V-4a, I1133 to N1229 of CRC-VI-2a or T579 to L680 of CRC-VI-3 (see also Figure S4) were cloned in the *Nco*I/*Xho*I restriction sites of the expression vector pRV11a [101], which contains a C-terminal His_6_ tag for purification of the recombinant peptides. His_6_-tagged fusion proteins were overexpressed by using *E.coli* strain Rosetta 2(DE3)pLysS (Merck4Biosciences, Novagen, Darmstadt, Germany).

### Purification of recombinant CRC-specific peptides and generation of polyclonal antibodies

Purification of the His_6_-tagged polypeptides was performed by a two-step procedure, whereby first inclusion bodies were prepared, followed by metal affinity chromatography. To obtain inclusion bodies, the induced bacteria were harvested and resuspended in ddH_2_O supplemented with 20 µg/ml lysozyme. Bacteria were lysed by one freeze/thaw cycle, followed by adding Triton X-100 to a final concentration of 0.5%. After sonication (three times, 30 s, 80 W) the inclusion bodies were pelleted by centrifugation (24,000× g for 20 min at 4°C) and then dissolved in 100 mM sodium phosphate buffer pH 8.0, 10 mM Tris-HCl (CRC-I-1, CRC-V-4, CRC-VI-3) or in 50 mM sodium phosphate buffer pH 7.0, 500 mM NaCl (CRC-III-4; CRC-VI-2) supplemented with 6 M guanidine hydrochloride.

Polypeptides derived from CRC-I-1, CRC-V-4 and CRC-VI-3 were further purified by affinity chromatography on Ni^2+^-nitriloactetate agarose under denaturing conditions according to the manufacturer's protocol (Merck4Biosciences, Novagen). The recombinant peptides were eluted with a pH step gradient, pH 8.0 to pH 4.5 in 8 M urea dissolved in 100 mM sodium phosphate buffer and 10 mM Tris-HCl. CRC-III-4 and CRC-VI-2 peptides were also purified under denaturating conditions but for affinity chromatography a TALON (Co^2+^) metal affinity resin (BD Biosiences, Clontech, Heidelberg, Germany) was used. Binding, washing and elution of CRC-III-4 and CRCR-VI-2 peptides was performed as recommended by the manufacturer.

The fractions collected were analyzed on SDS polyacrylamide gels, brought to neutral pH and used for immunization of rabbits. As CRC-III-4 and CRC-VI-2 peptides were eluted in buffer containing 6 M guanidine hydrochloride, pooled fractions were dialyzed against 100 mM sodium phosphate buffer pH 7.5 supplemented with 8 M urea. Before immunization, a pool of rabbits was tested for suitability, i.e. for background staining in immuno-fluorescence assays and Western blots. After several boosts, positive sera were taken and specific antibodies were purified by two subsequent chromatography steps: First, anti-His-tag antibodies were removed by adsorption against an immobilized His-tagged protein, followed by a second affinity step using the corresponding CRC-specific polypeptide.

### Construction of CRC-VI-3 GFP expression plasmid

CRC-VI-3 sequences were amplified by PCR (for oligonucleotides see Table S2) using the iProof High-Fidelity DNA Polymerase (Bio-Rad) and macronuclear DNA as template. The PCR reaction was carried out in 35 cycles of 10 s denaturation at 98°C, 15 s annealing at 58°C and 200 s elongation at 72°C. The 6236 bp PCR-product was subcloned in the Zero Blunt TOPO cloning vector (Invitrogen) and positive clones were sequenced. Correct inserts were cloned into the enhanced green fluorescent protein (eGFP) expression plasmid pPXV-GFP [83] between *Mlu*I and *Xho*I sites in-frame with the gene encoding eGFP, which was positioned at the N-terminus of CRC-VI-3. Positive clones were verified by sequencing and further processed for microinjection.

### Microinjection experiments

Overexpression of the GFP-CRC-VI-3 fusion protein was achieved by microinjection of the CRC-VI-3 GFP plasmid DNA into the macronucleus. To stabilize the DNA in the macronucleus after microinjection, 50 µg pPXV-CRC-VI-3 plasmid DNA was linearized with 20 units of *Sfi*I to remove an internal vector sequence between two inverted telomeric repeats from *Tetrahymena thermophila*. After digestion at 50°C overnight, the DNA was precipitated by adding 1/10 (v/v) 3 M sodium acetate buffer pH 5.2 and 2.5 (v/v) ethanol, pelleted by centrifugation and washed with 70% ethanol. The air dried pellet was resuspended in 10 µl of Millipore-filtered water followed by an additional centrifugation step of 30 min at 4°C. The supernatant was transferred in a new tube to remove any insoluble material that could block the microinjection capillaries.

Microinjections were performed with postautogamous cells which were grown for three generations in salad medium pre-inoculated with *Enterobacter aerogenes*. To avoid interferences during microinjection, cells were pre-treated with aminoethyldextran at 0.005% in 10 mM Tris-HCl pH 7.0, with 1 mM CaCl_2_ added, to trigger trichocyst discharge. After two washing steps in Dryl's buffer (2 mM sodium citrate, 1 mM NaH_2_PO_4_, 1 mM Na_2_HPO_4_, 1.5 mM CaCl_2_, pH 6.9) containing 0.2% bovine serum albumin (BSA), individual cells were transferred under a dissecting microscope into small droplets on a glass slide and covered with paraffin oil to prevent evaporation. Microinjections into the macronucleus were made with glass microcapillaries under an inverted microscope (Axiovert 100TV, Zeiss, Oberkochen, Germany) using a DeFonbrune micromanipulator and a manually controlled air-pressure microinjector.

### Immuno-fluorescence labeling

Immuno-stainings were performed after fixing cells, suspended in PIPES-buffer pH 7.0 (5 mM Pipes = piperazine-1,4-bis[2-ethanesulfonic acid] pH 7.0, 1 mM KCl and 1 mM CaCl_2_), for 25 min at room temperature, either in precooled 4% (w/v) freshly prepared formaldehyde in phosphate buffered saline (PBS) supplemented with 1% Triton X-100 (Sigma-Aldrich, St. Louis, MO) or in 4% formaldehyde in PBS, followed by adding digitonin (Sigma-Aldrich) to a final concentration of 0.5%. Fixed cells were washed three times in PBS, incubated twice in PBS with 50 mM glycine for 10 min and finally in PBS with 1% BSA added. Then primary antibodies; diluted in PBS with 1% BSA, were applied for 60 min at room temperature, followed by several washing steps with PBS. Thereafter, samples were incubated with AlexaFluor 488- or 594-conjugated F(ab′)_2_ fragments of goat anti-rabbit and goat anti-mouse IgG (Molecular Probes, Invitrogen) diluted 1∶200 in PBS with 1% BSA added. Finally, cells were washed four times with PBS and mounted in Mowiol for fluorescence microscopy.

To improve labeling of cytoskeletal and cortical structures, the method described by Beisson et al., 2010 [102] was applied with some modifications. In brief, cells were permeabilized for 2 min with 1% Triton X-100 in PHEM buffer (60 mM Pipes, 25 mM Hepes (N-[2-hydroxyethyl]-piperazine-N′-2-ethanesulfonic acid), 10 mM EGTA (ethylene glycol tetraacetic acid), 2 mM MgCl_2_, pH 6.9) followed by 10 min of fixation in 2.5% formaldehyde in PHEM buffer. After two washing steps in Tris-buffered saline, TBS, (10 mM Tris-HCl, 15 mM NaCl, pH 7.4) with 0.1% Tween 20 and 3% BSA (TBST*), samples were incubated for 60 min with primary antibodies diluted in TBST*. After three washing steps with TBST*, secondary antibodies (see above) diluted in TBST* were applied for additional 60 min. Samples were then washed three times with TBST* and mounted in Mowiol.

Primary affinity-purified antibodies against CRC-I-1, CRC-III-4, CRC-V-4 and CRC-VI-3 were applied at a dilution of 1∶100; CRC-VI-2-specific antibodies were diluted 1∶50. The mouse monoclonal antibody against α-tubulin (clone DM1A; Sigma-Aldrich) was used at a dilution of 1∶400. GFP fluorescence was enhanced by using a polyclonal anti-GFP antibody [103] diluted 1∶200. ER structures were stained either with mouse polyclonal antibodies against protein disulfide-isomerase (PDI) as previously described [25] or with 3,3′-dihexaoxacarbocyanine iodide (DiOC_6_; Sigma-Aldrich) at a final concentration of 0.1 µg per ml PBS for 45 min. For controls, pre-immune sera (diluted 1∶100) were used or primary antibodies were omitted.

### Deciliation of *Paramecium* cells

To ensure labeling of basal bodies with antibodies against α-tubulin, cells were deciliated as described by Nelson, 1995 [104]. In brief, cells were washed twice in PIPES-buffer pH 7.0 and resuspended in 50 mM MnCl_2_ in 10 mM Tris-HCl, pH 7.2 at room temperature. Cells were removed by centrifugation and resuspended in the same solution. After 5 min of gentle shaking, 90–95% of cells were deciliated and removed by centrifugation, followed by two wash steps in PIPES-buffer pH 7.0 before further use.

### Fluorescence microscopy

Epifluorescence analysis was performed using an Axiovert 100TV (Zeiss) equipped with a plan-Neofluar ×40 oil immersion objective (NA 1.3) and a C-Apochromat ×63 water immersion objective (NA 1.2) combined with filter set 9, or, when GFP-fluorescence was monitored, with filter set 13. Images were acquired with a ProgRes C10 plus camera and ProgRes Capture Basic software (Jenoptik, Jena, Germany) and further processed with Adobe Photoshop (Adobe Systems, San Jose, CA). Confocal microscopy was performed with a CLSM 510 equipped with a Plan-Apochromat ×63 oil immersion objective (NA 1.4) (both from Zeiss). Images acquired with the LSM software were analyzed with LSM image browser (Zeiss) or with the public domain NIH ImageJ program (http://rsbweb.nih.gov/ij/) and further edited with Photoshop software (Adobe Systems).

### Immuno-EM analyses

Cells were injected into a fixative (0°C) of 8% formaldehyde with 0.1% glutaraldehyde dissolved in PIPES-buffer pH 7.2 with a quenched-flow apparatus [105]. After 60 min of fixation, cells were washed twice with PBS containing 50 mM glycine for 10 min. Then samples were dehydrated by increasing ethanol concentrations (30%, 50%, 70%, 90%, 96%, 2×15 min each, and 2×100%, 30 min each), followed by LR Gold methacrylate resin embedding (London Resin, London, UK) and UV polymerization at −35°C. Ultrathin sections were incubated with CRC-specific antibodies, followed by protein A-gold conjugated to colloidal gold particles of 5 nm (pA-Au_5_ from Dept. Pathobiol., University of Utrecht, NL) and analyzed in a Zeiss electron microscope, EM10. For controls, primary antibodies were omitted or irrelevant ones were applied.

## Supporting Information

Figure S1 **Re-annotations of** ***Paramecium*** **CRCs.** Schematic representation of the re-annotated CRC sequences (green arrows), which show deviations from their corresponding genes published in the *Paramecium*DB (http://paramecium.cgm.cnrs-gif.fr; blue arrows). The positions of introns (triangles) are indicated with regard to the transcriptional start site of the respective CRC gene and to their positions within the relevant genomic scaffold. Deviations from the sequences published in the *Paramecium*DB are highlighted in red. (A) Comparison of *CRC-I-1a* sequences with corresponding sections of the *Paramecium*DB. The corrected *CRC-I-1a* gene (Acc. No. FR877768) is located between positions 58330 and 49331 on scaffold 9. By error the automated annotation has assumed that the start ATG of *CRC-I-1a* is at position 56970 (Gene ID: GSPATG00003859001), which was corrected to position 58330 corresponding to the start ATG of a gene (Gene ID: GSPAT0000386001) located upstream of GSPATG00003859001. Sequencing of the cDNA and macronuclear DNA leads to the identification of five wrong insertions at positions 54258 (-T), 56814 (-A), 56815 (-T), 57199 (-A) and 57211(-C) and to the removal of three incorrect introns between positions 54243–54264, 56806–56825 and 56933–56949. The corrected *CRC-I-1a* gene possesses three introns and encodes a protein of 2972 amino acids. (B) cDNA analysis of *CRC-I-1c* reveals that, in comparison to the annotated gene of the *Paramecium*DB (Gene ID GSPAfTT00027332001), the position of the start ATG is shifted by 194 nucleotides towards its 3′-end. The corrected *CRC-I-1c* gene (Acc. No. FR877769) is located between positions 217928 (start ATG) and 208964 (stop TGA) of scaffold 98 and encodes a protein of 2980 amino acids. (C) Sequencing of sections of *CRC-I-2a* shows that one postulated intron of 33 bp between positions 542638 and 542715 of scaffold 20 of the annotated sequence (Gene ID GSPATT00008240001) is not spliced. Therefore, the re-annotated *CRC-I-2a* gene (Acc. No. FR877770) encodes a protein with 3010 amino acids instead of 2999 amino acids (*Paramecium*DB). (D) Re-annotation of *CRC-III-1a* results in the modification of one postulated intron of 37 bp between nucleotides 310202 and 310238 of scaffold 86. With the corrected version (Acc. No. FR877771) this intron is shortened by nine nucleotides, resulting in an ORF of 7797 bp instead of 7788 bp as published in the *Paramecium*DB. (E) Sequencing of CRC-III-1b reveals that two postulated introns of 19 bp and 27 bp on Sc 158 between nucleotides 37217 to 37235 and 36842 to 36868 are not spliced, whereas adjacent introns are. To obtain full-length transcripts, a deletion of one nucleotide at position 37131 (-A) would be necessary (which is the case with the ohnolog CRC-III-1a). However, none of our seven sequenced clones possesses such a deletion which is also absent in 15 single reads covering this region in the *Paramecium*DB. Therefore the CRC-III-1b transcripts result in a premature stop codon at position 37017, indicating that this gene is pseudogenized. (F) Comparison of the re-annotated *CRC-III-2* gene (Acc. No. FR877772) with the annotated gene in the *Paramecium*DB (Gene ID GSPATT00012849001). The position of the start ATG is shifted by 2134 nucleotides, thereby overlapping with the start codon of an upstream gene GSPATT00012848001. The corrected *CRC-III-2* gene possesses an extra intron between positions 449313 and 449337 of scaffold 35 and encodes a protein of 2738 aa. (G) Analysis of *CRC-III-4c* cDNA sequences results in the modification of one postulated intron of 32 bp between positions 159692 and 159723 of scaffold 37. With the corrected version (Acc. No. FRFR877773) this intron is shortened by six nucleotides, resulting in an ORF of 8505 bp instead of 8511 bp as published in the *Paramecium*DB. (H) Sequencing of 3′-parts of *CRC-IV-3b* shows that one postulated intron of 24 bp between positions 277075 and 277098 of scaffold 24 of the annotated sequence (Gene ID GSPATT00009387001) is not spliced. Therefore, the re-annotated *CRC-IV-3b* gene (Acc. No. FR877774) encodes a protein with 3127 amino acids instead of 3121 amino acids (*Paramecium*DB). (I) Sequencing of sections of *CRC-V-4b* reveals an additional intron of 27 bp between positions 259205 and 259231 of scaffold 106. Therefore, the re-annotated *CRC-V-4b* gene (Acc. No. FR877775) encodes a protein with 2589 amino acids instead of 2598 amino acids (*Paramecium*DB, Gene ID GSPATT00028778001). (J) Comparison of the re-annotated *CRC-VI-2b* sequences (Acc. No. FR877776) with their *Paramecium*DB counterparts (Gene ID: GSPATT00015337001). Sequencing of the 3′-end shows, that the position of one intron is shifted downstream by 88 nucleotides (from position 373700/373725 to 373814/373839 of scaffold 44). Additionally we identified two wrong insertions at position 374228 (-C) and 374253 (-T) of scaffold 44 resulting in the loss of one postulated intron between positions 374218 and 374234. The corrected *CRC-VI-2b* gene possesses five introns and encodes a protein of 2774 amino acids. (K) Re-annotation of *CRC-VI-3*. The corrected *CRC-VI-3* gene (Acc. No. FR877777) is located between positions 230703 and 236920 on scaffold 134. In the *Paramecium*DB, the start ATG of this gene was wrongly assumed to occur at position 234422 of scaffold 134 (Gene ID: GSPATG00033125001). The correct start ATG (position 236920) corresponds to the start ATG of a gene (Gene ID: GSPAT000331260001) located upstream of GSPATG0003312500. Furthermore, cDNA analysis reveals an extra intron between nucleotides 234868 and 234889 and an intragenic region (x; purple box) between positions 234586 and 234611, which was found to be spliced in several RNA molecules. Re-annotation of *CRC-VI-3* results in a gene of 6218 bp with six introns encoding a protein of 2021 amino acids.
(PDF)

Figure S2 **Phylogeny of the C-terminal channel domains of** ***Paramecium*** **CRCs according to maximum parsimony analysis.** Phylogenetic relationships calculated with the maximum parsimony algorithm encompassing the transmembrane domains of *Parameciu*m CRC proteins and metazoan InsP_3_ and ryanodine receptor sequences from *Mus musculus* (Acc No: NP_034715.2, NP_033135), *Drosophila melanogaster* (Acc No: BAA14399.1, NP_033135) and *Caenorhapditis elegans* (Acc No. NP_001023173, BAA08309). The flanking residues of the peptides used are in parenthesis. Bootstrap support values for the nodes were calculated with 1000 replicates and are given at the branches.
(TIF)

Figure S3 **Evolutionary relationship of the re-annotated** ***Paramecium*** **CRC proteins.** Neighbor-joining tree (with 1000 bootstrap replicates) representing phylogenetic relationships between the re-annotated CRC proteins and three different metazoan InsP_3_ receptors, which are from *Mus musculus* (*Mm*InsP_3_R type 1, Acc No: NP_034715.2), *Drosophila melanogaster* (*Dm*InsP_3_R, Acc No: BAA14399.1) or *Caenorhabditis elegans* (*Ce*ITR1, Acc No: NP_001023173). The tree supports previously published data showing that the 34 CRC proteins cluster in six different groups [25]. However, the arrangement of group IV and V channels changes (red double arrow): CRC-V channels now cluster with the clade of group I, II and III channels. This is in agreement with CRC-V architecture as these channel types possess, similar to group I, II and III channels, conserved N-terminal parts, which are mostly absent in group IV and VI channels (see Figure 2A). Accession numbers of the re-annotated sequences are summarized in table 1. Bootstrap support values for the nodes are shown, and evolutionary distances are given by the scale bar below. ‘Sc’ denotes scaffold numbers according to macronuclear DNA sections as designated in the *Paramecium*DB (http://paramecium.cgm.cnrs-gif.fr).
(TIF)

Figure S4 **Antibodies against** ***Paramecium*** **CRCs.** (A) Schematic view of CRC proteins selected for antibody production with the positions of immunogenic peptides indicated. Note that each group of channels is represented by at least one CRC protein. In this paper, we put the emphasis on results obtained with antibodies against CRC-I-1, CRC-III-4, CRC-V-4, CRC-VI-2 and CRC-VI-3, while antibodies against CRC-II-1a and CRC-IV-1a have been described previously [24], [25]. Additionally, we outline regions homologous to IP_3_ receptors (grey bars) and ryanodine receptors (green bars), which were determined by BLAST analyses [96]. Positions of flanking residues of putative channel domains are highlighted in red and IP_3_ binding domains in blue. RIH: RyR and IP_3_R homology domain according to Ponting [14]; Sc: scaffold number. (B–F) Characterization of CRC-specific antibodies in Western blots. Affinity-purified antibodies against CRC-I-1 (B), CRC-III-4 (C), CRC-V-4 (D), CRC-VI-2 (E) and CRC-VI-3 (F) recognize their respective immunogenic peptides (AG) with high affinity in immuno-blots (lanes 3, 4 and 5, respectively), whereas the relevant pre-immunsera (PIS) yield no signals (lanes 2). The purified polypeptides used for immunization are visualized by Coomassie staining (lanes 1; COOM).
(TIF)

Figure S5 **Specification of antibodies prepared for the different CRC types in immuno-fluorescence studies.** Epifluorescence microscopy of cells stained either with CRC-specific antibodies or with their respective rabbit pre-immunsera (PIS). Pre-immunsera did not result in any specific labeling (A, D, I, N and Q) and fluorescence signals were comparable with those observed in controls when primary antibodies were omitted (data not shown). (A, B and C) Rabbit pre-immunserum R1034 (A) compared with purified antibodies raised against CRC-I-1 (B). The staining pattern of CRC-I-1 specific antibodies is remarkably similar to staining obtained with antibodies against the ER-resident protein disulfide isomerase (PDI, C). (D–H) In contrast to the pre-immunserum R1065 (D), CRC-III-4 specific antibodies recognize dotted areas (E, G) in posterior parts of the cell. Co-staining of these cells with antibodies against α-tubulin (F, H) show that CRC-III-4 labeling occurs in regions between the oral apparatus (oa) and the cytoproct (cp). (I–M) Comparison of CRC-V-4 specific antibodies with the rabbit pre-immunserum R1018 (I) reveals specific CRC-V-4 labeling. (J) Micrograph showing a cell at an early stage of division, as the contractile vacuole complexes have already doubled. CRC-V-4 labeling occurs at the old contractile vacuole complexes and at the newly formed ones. Additionally, strong fluorescence signals could be observed at the oral apparatus (J) and at the cell cortex (L). The cortical punctate pattern in (L) is similar to basal body (bb) staining obtained with α-tubulin antibodies (M). (N, O and P) Labeling with CRC-VI-2-specific antibodies results in a surface associated network along the longitudinal and perpendicular ridges of the cell surface (O), whereas the pre-immunserum R1044 (N) does not reveal such a pattern. (Q–U) A dotted cortical pattern is visible when cells are stained with CRC-VI-3 specific antibodies (R, T). A dorsal view of such cells shows staining of the pores of the contractile vacuole complex (T). In comparison, no fluorescence could be observed with the pre-immunserum R1064 (Q). Abbreviations: contractile vacuole complex (cvc), contractile vacuole (cv), radial canals (rc), oral apparatus (oa), pof post oral fibers (pof), basal body (bb), pore of the contractile vacuole complex (po), cp (cytoproct), anterior (a), posterior (p). Bars = 10 µm.
(TIF)

Figure S6 **Immunogold EM localization of CRC-I-1.** EM micrographs of ER-rich domains in peripheral (A) and central (B) regions of the cell. Immunogold labeling with CRC-I-1-specific antibodies is visible close to ER membranes (arrows), while mitochondria (mi) or acidosomes (ac) are not labeled. Bars = 0.1 µm.
(TIF)

Table S1 **Predictions of transmembrane regions in** ***Paramecium*** **CRC proteins.** Results of transmembrane predictions using the TOPCONS algorithm (http://topcons.net/, [38]) obtained with full-length proteins (fl) or C-terminal regions as input queries. Putative transmembrane helices printed in red show slight deviations between the different queries, those printed in green represent results of one of the five topology predictions integrated in TOPCONS. The results retrieved for the CRC-III-1bΨ pseudogene are outlined in grey or bright red. Furthermore, full-length proteins were analyzed with TMHMM v.2.0 (http://www.cbs.dtu.dk/services/TMHMM/, [39]) and Kyte Doolitte hydrophobicity scales [40]. Additionally to the *Paramecium* CRC sequences, we analyzed three different metazoan IP_3_ and ryanodine receptors, which are from *Mus musculus* (*Mm*IP_3_R type 1, Acc No: NP_034715.2; *Mm*RyR type 1, Acc No: NP_033135), *Drosophila melanogaster* (*Dm*IP_3_R, Acc No: BAA14399.1; *Dm*RyR, Acc No: NP_033135) or *Caenorhabditis elegans* (*Ce*ITR1, Acc No: NP_001023173; *Ce*RyR, Acc No: BAA08309).
(DOC)

Table S2 **Oligonucleotide primers used for amplification and cloning of CRC genes.**
(DOC)
