# Supplementary figures and images for: Calcium-Release Channels in Paramecium. Genomic Expansion, Differential Positioning and Partial Transcriptional Elimination
Source: PLoS One. 2011 Nov 10;6(11):e27111. doi: 10.1371/journal.pone.0027111 (PMC3213138; doi:10.1371/journal.pone.0027111)

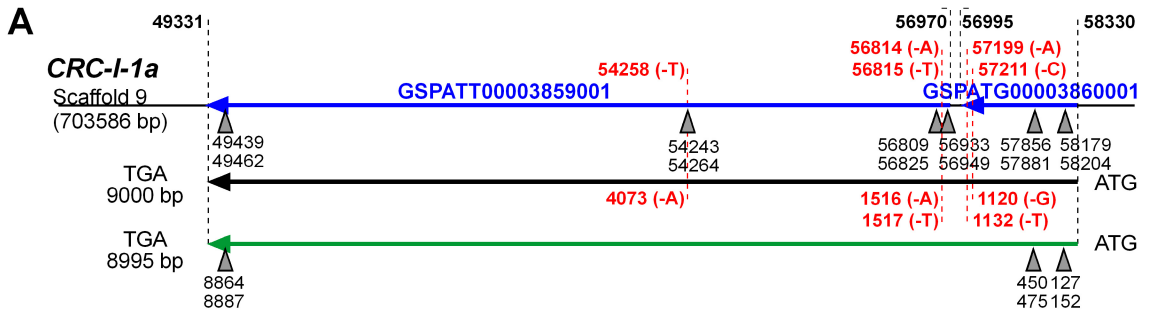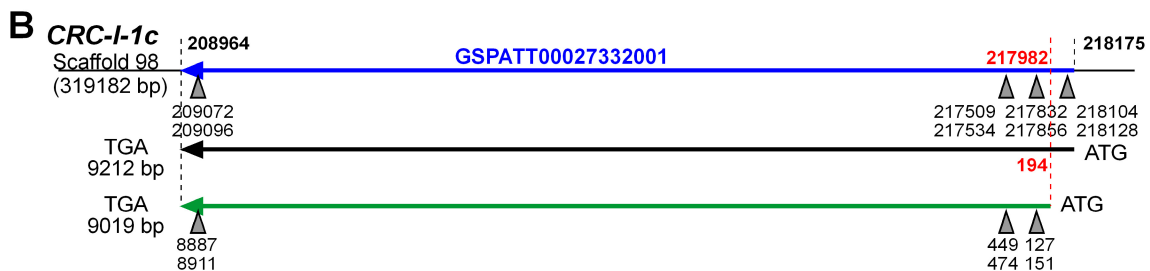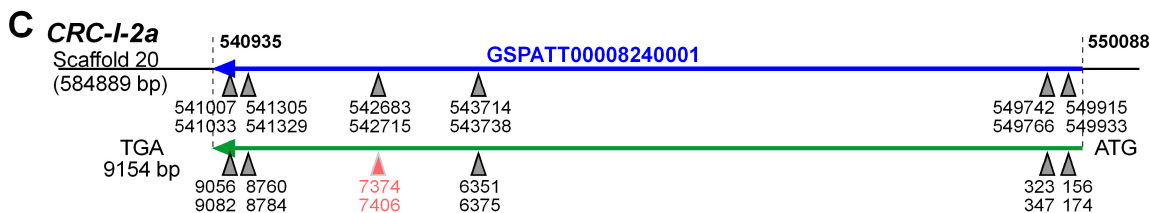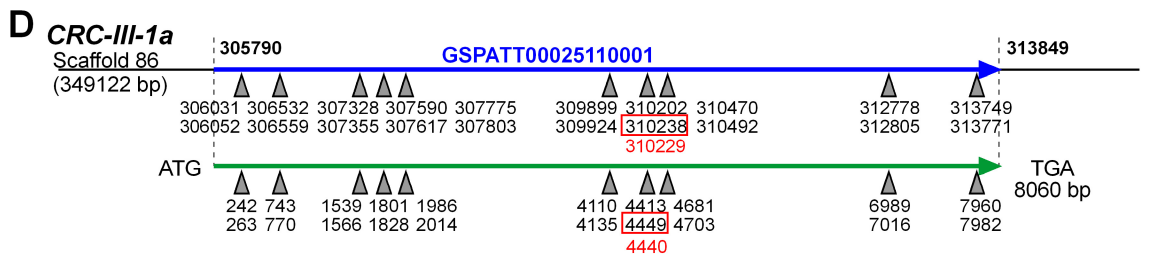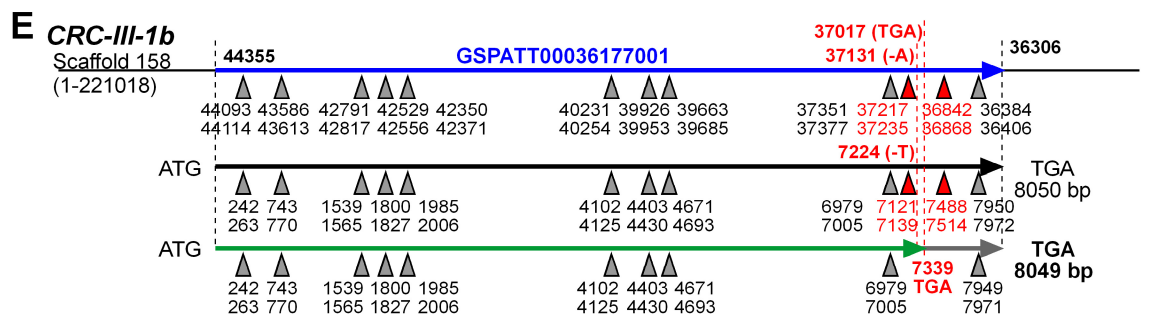

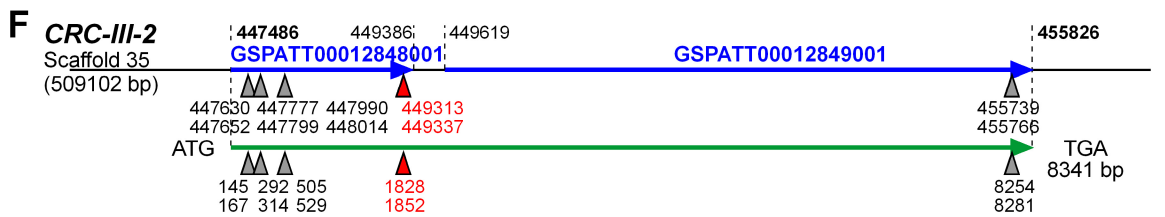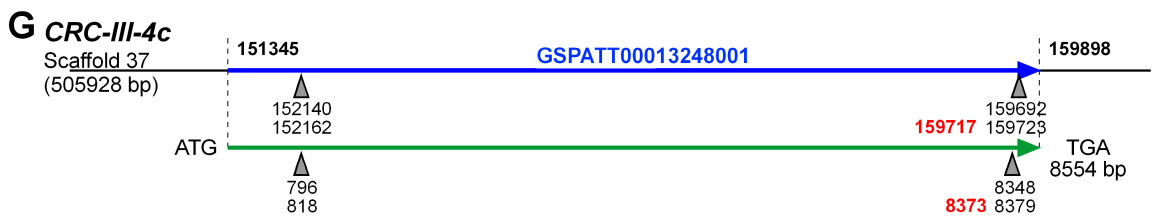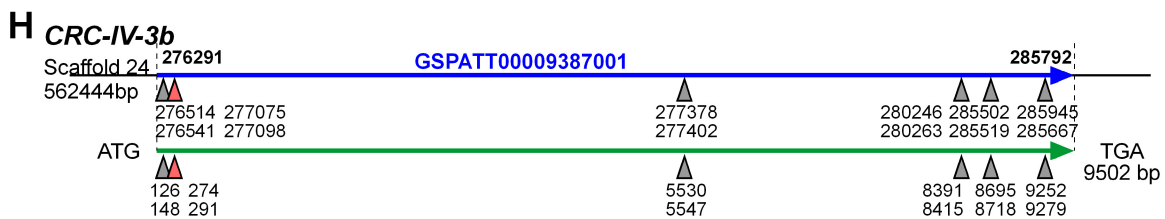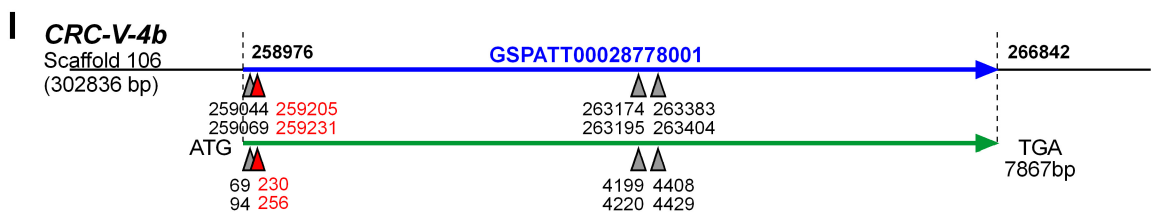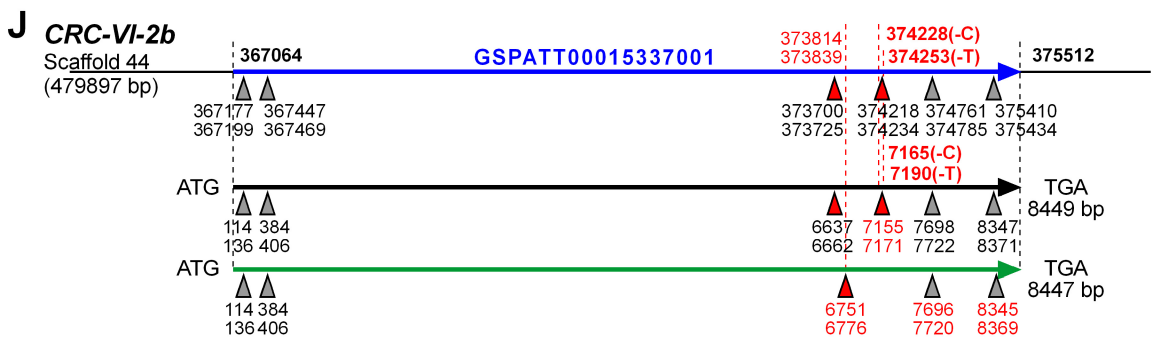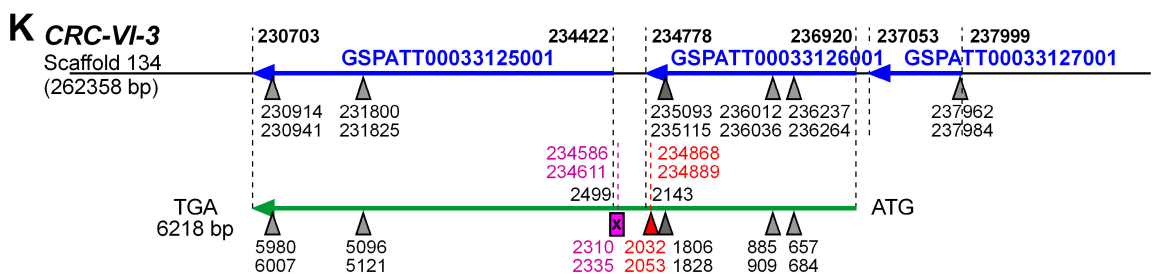

Supplement: Figure S1 — Re-annotations of Paramecium CRCs. Schematic representation of the re-annotated CRC sequences (green arrows), which show deviations from their corresponding genes published in the ParameciumDB (http://paramecium.cgm.cnrs-gif.fr; blue arrows). The positions of introns (triangles) are indicated with regard to the transcriptional start site of the respective CRC gene and to their positions within the relevant genomic scaffold. Deviations from the sequences published in the ParameciumDB are highlighted in red. (A) Comparison of CRC-I-1a sequences with corresponding sections of the ParameciumDB. The corrected CRC-I-1a gene (Acc. No. FR877768) is located between positions 58330 and 49331 on scaffold 9. By error the automated annotation has assumed that the start ATG of CRC-I-1a is at position 56970 (Gene ID: GSPATG00003859001), which was corrected to position 58330 corresponding to the start ATG of a gene (Gene ID: GSPAT0000386001) located upstream of GSPATG00003859001. Sequencing of the cDNA and macronuclear DNA leads to the identification of five wrong insertions at positions 54258 (-T), 56814 (-A), 56815 (-T), 57199 (-A) and 57211(-C) and to the removal of three incorrect introns between positions 54243–54264, 56806–56825 and 56933–56949. The corrected CRC-I-1a gene possesses three introns and encodes a protein of 2972 amino acids. (B) cDNA analysis of CRC-I-1c reveals that, in comparison to the annotated gene of the ParameciumDB (Gene ID GSPAfTT00027332001), the position of the start ATG is shifted by 194 nucleotides towards its 3′-end. The corrected CRC-I-1c gene (Acc. No. FR877769) is located between positions 217928 (start ATG) and 208964 (stop TGA) of scaffold 98 and encodes a protein of 2980 amino acids. (C) Sequencing of sections of CRC-I-2a shows that one postulated intron of 33 bp between positions 542638 and 542715 of scaffold 20 of the annotated sequence (Gene ID GSPATT00008240001) is not spliced. Therefore, the re-annotated CRC-I-2a gene (Acc. No. FR877770) [file pone.0027111.s001.pdf]

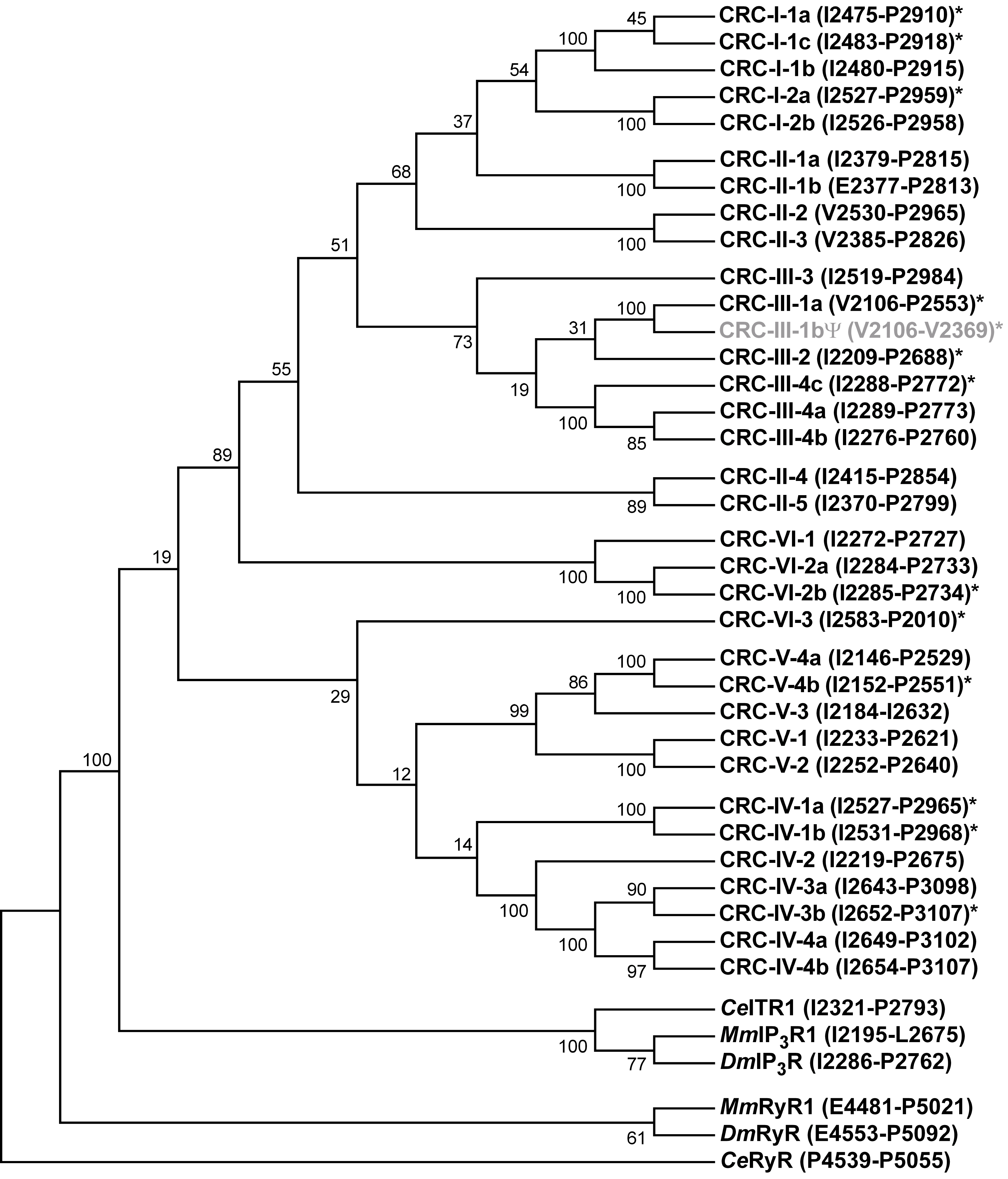

Supplement: Figure S2 — Phylogeny of the C-terminal channel domains of Paramecium CRCs according to maximum parsimony analysis. Phylogenetic relationships calculated with the maximum parsimony algorithm encompassing the transmembrane domains of Paramecium CRC proteins and metazoan InsP3 and ryanodine receptor sequences from Mus musculus (Acc No: NP_034715.2, NP_033135), Drosophila melanogaster (Acc No: BAA14399.1, NP_033135) and Caenorhapditis elegans (Acc No. NP_001023173, BAA08309). The flanking residues of the peptides used are in parenthesis. Bootstrap support values for the nodes were calculated with 1000 replicates and are given at the branches. (TIF) [file pone.0027111.s002.tif]

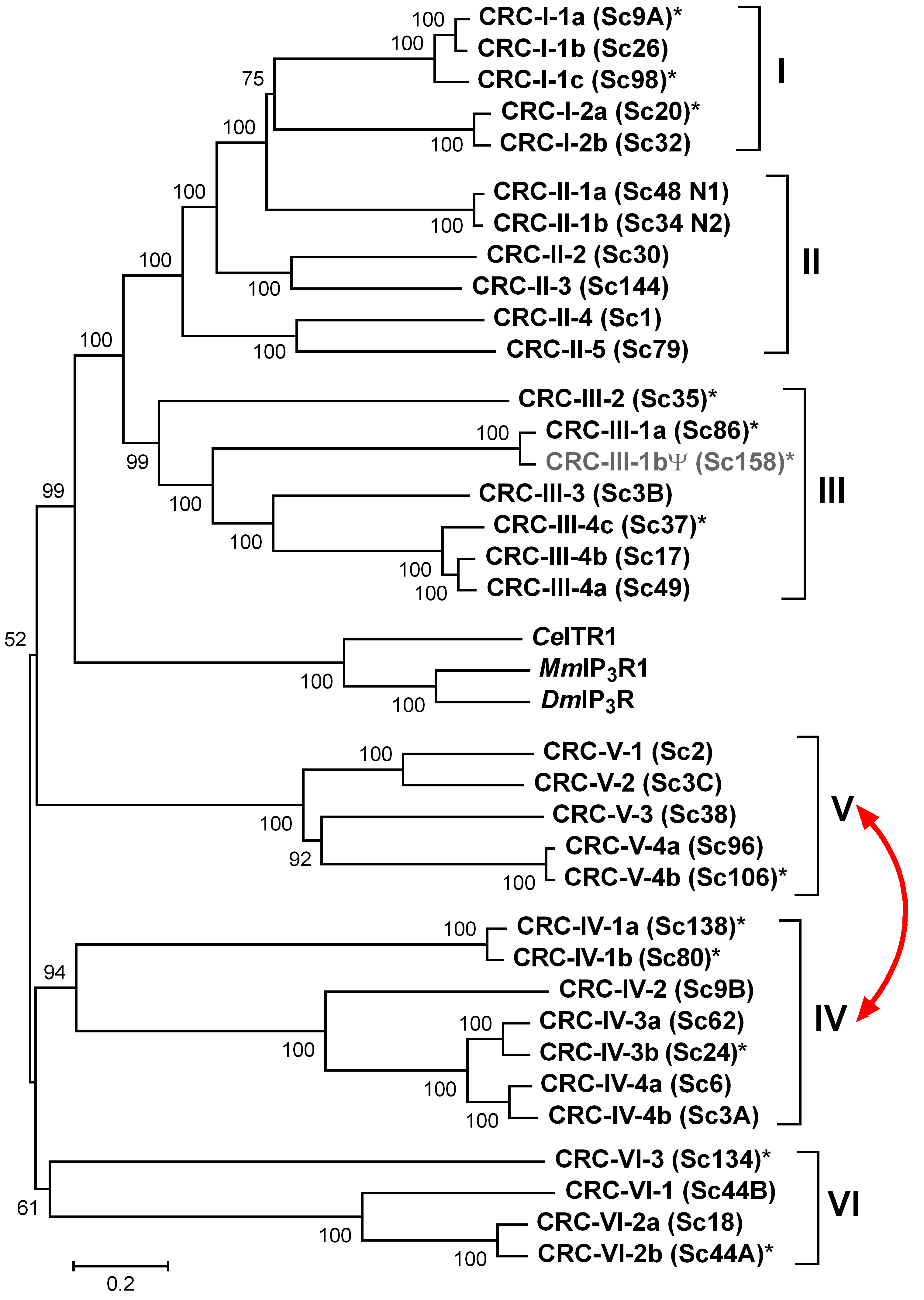

Supplement: Figure S3 — Evolutionary relationship of the re-annotated Paramecium CRC proteins. Neighbor-joining tree (with 1000 bootstrap replicates) representing phylogenetic relationships between the re-annotated CRC proteins and three different metazoan InsP3 receptors, which are from Mus musculus (MmInsP3R type 1, Acc No: NP_034715.2), Drosophila melanogaster (DmInsP3R, Acc No: BAA14399.1) or Caenorhabditis elegans (CeITR1, Acc No: NP_001023173). The tree supports previously published data showing that the 34 CRC proteins cluster in six different groups [25]. However, the arrangement of group IV and V channels changes (red double arrow): CRC-V channels now cluster with the clade of group I, II and III channels. This is in agreement with CRC-V architecture as these channel types possess, similar to group I, II and III channels, conserved N-terminal parts, which are mostly absent in group IV and VI channels (see Figure 2A). Accession numbers of the re-annotated sequences are summarized in table 1. Bootstrap support values for the nodes are shown, and evolutionary distances are given by the scale bar below. ‘Sc’ denotes scaffold numbers according to macronuclear DNA sections as designated in the ParameciumDB (http://paramecium.cgm.cnrs-gif.fr). (TIF) [file pone.0027111.s003.tif]

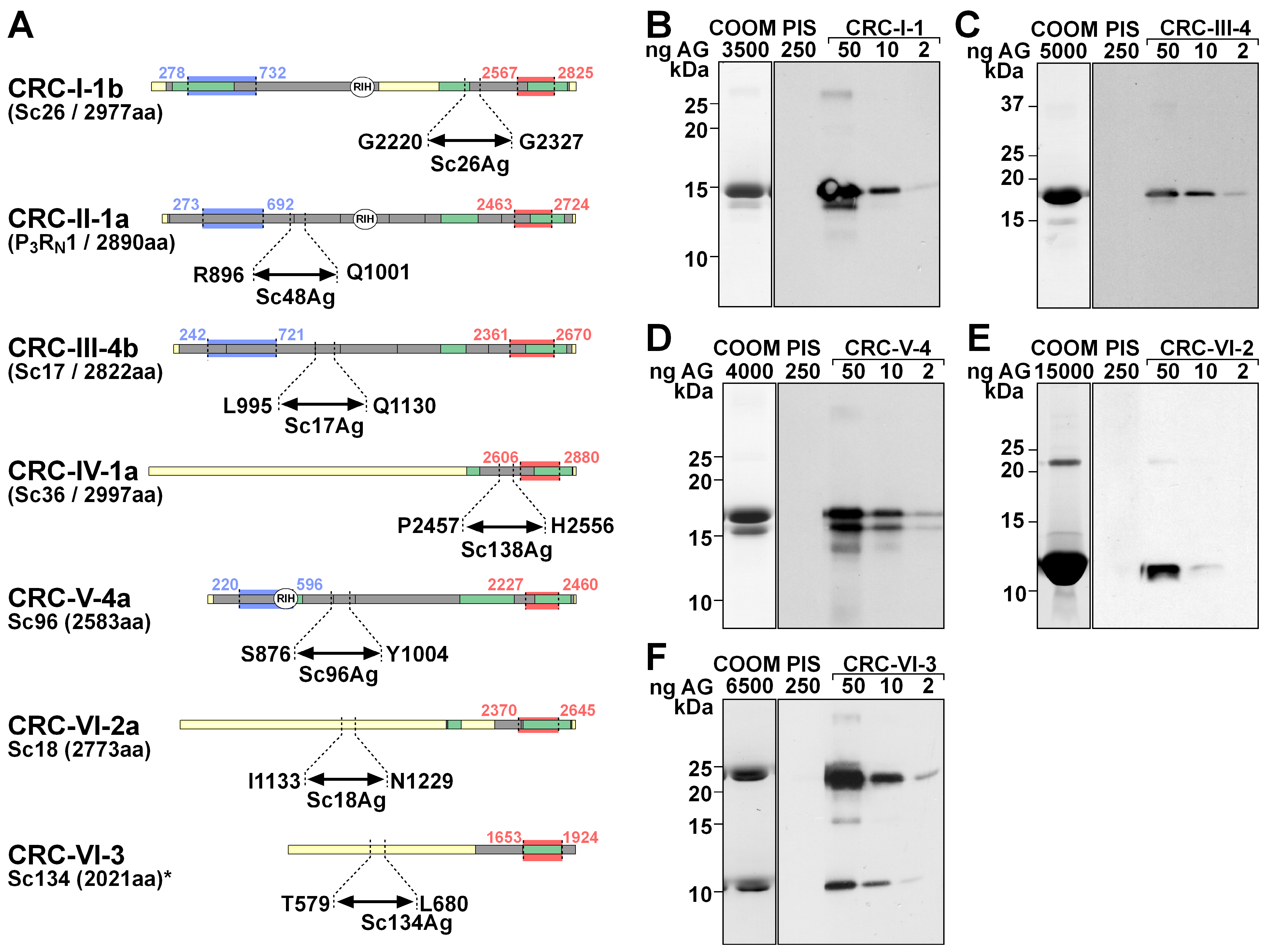

Supplement: Figure S4 — Antibodies against Paramecium CRCs. (A) Schematic view of CRC proteins selected for antibody production with the positions of immunogenic peptides indicated. Note that each group of channels is represented by at least one CRC protein. In this paper, we put the emphasis on results obtained with antibodies against CRC-I-1, CRC-III-4, CRC-V-4, CRC-VI-2 and CRC-VI-3, while antibodies against CRC-II-1a and CRC-IV-1a have been described previously [24], [25]. Additionally, we outline regions homologous to IP3 receptors (grey bars) and ryanodine receptors (green bars), which were determined by BLAST analyses [96]. Positions of flanking residues of putative channel domains are highlighted in red and IP3 binding domains in blue. RIH: RyR and IP3R homology domain according to Ponting [14]; Sc: scaffold number. (B–F) Characterization of CRC-specific antibodies in Western blots. Affinity-purified antibodies against CRC-I-1 (B), CRC-III-4 (C), CRC-V-4 (D), CRC-VI-2 (E) and CRC-VI-3 (F) recognize their respective immunogenic peptides (AG) with high affinity in immuno-blots (lanes 3, 4 and 5, respectively), whereas the relevant pre-immunsera (PIS) yield no signals (lanes 2). The purified polypeptides used for immunization are visualized by Coomassie staining (lanes 1; COOM). (TIF) [file pone.0027111.s004.tif]

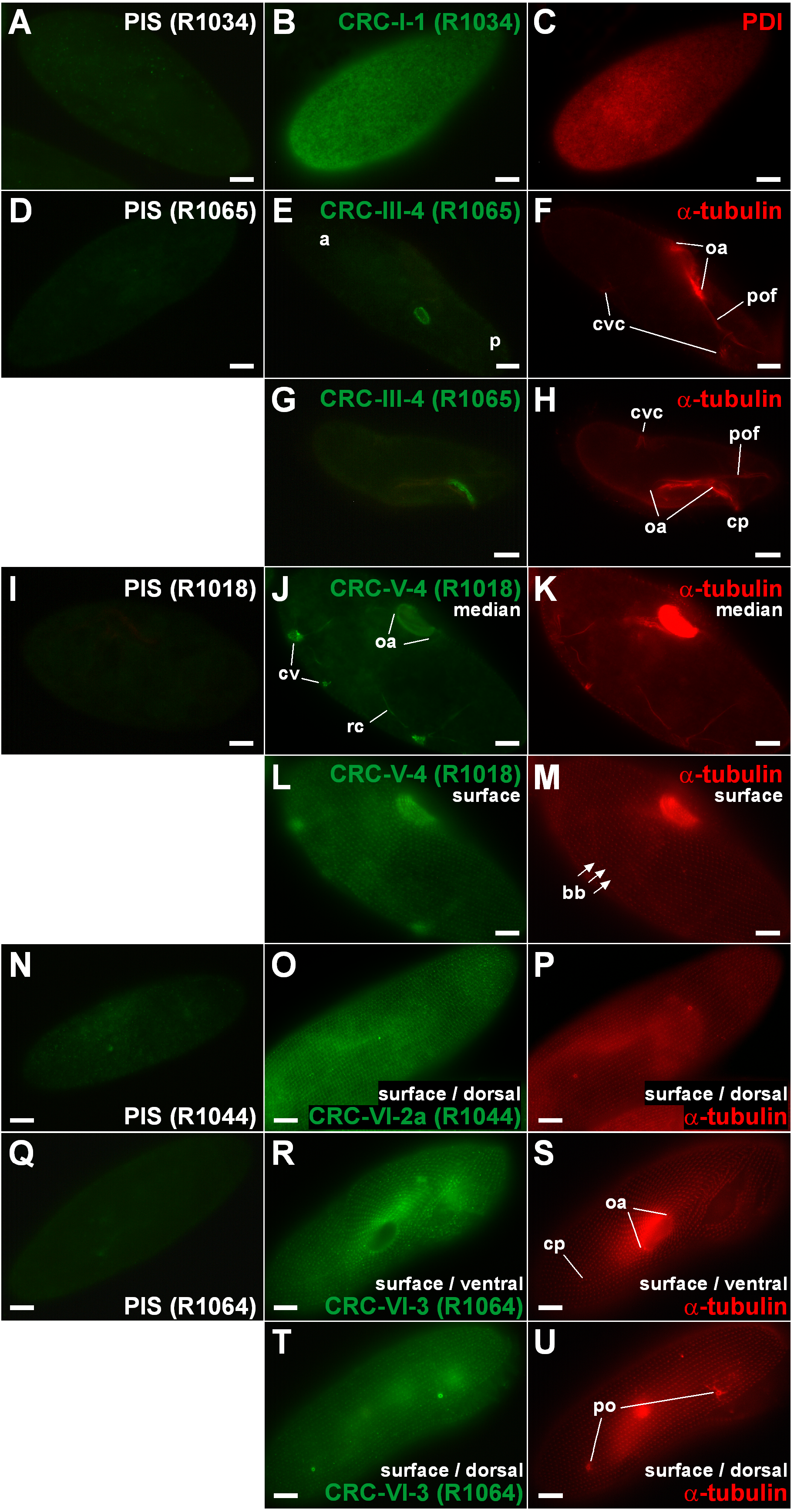

Supplement: Figure S5 — Specification of antibodies prepared for the different CRC types in immuno-fluorescence studies. Epifluorescence microscopy of cells stained either with CRC-specific antibodies or with their respective rabbit pre-immunsera (PIS). Pre-immunsera did not result in any specific labeling (A, D, I, N and Q) and fluorescence signals were comparable with those observed in controls when primary antibodies were omitted (data not shown). (A, B and C) Rabbit pre-immunserum R1034 (A) compared with purified antibodies raised against CRC-I-1 (B). The staining pattern of CRC-I-1 specific antibodies is remarkably similar to staining obtained with antibodies against the ER-resident protein disulfide isomerase (PDI, C). (D–H) In contrast to the pre-immunserum R1065 (D), CRC-III-4 specific antibodies recognize dotted areas (E, G) in posterior parts of the cell. Co-staining of these cells with antibodies against α-tubulin (F, H) show that CRC-III-4 labeling occurs in regions between the oral apparatus (oa) and the cytoproct (cp). (I–M) Comparison of CRC-V-4 specific antibodies with the rabbit pre-immunserum R1018 (I) reveals specific CRC-V-4 labeling. (J) Micrograph showing a cell at an early stage of division, as the contractile vacuole complexes have already doubled. CRC-V-4 labeling occurs at the old contractile vacuole complexes and at the newly formed ones. Additionally, strong fluorescence signals could be observed at the oral apparatus (J) and at the cell cortex (L). The cortical punctate pattern in (L) is similar to basal body (bb) staining obtained with α-tubulin antibodies (M). (N, O and P) Labeling with CRC-VI-2-specific antibodies results in a surface associated network along the longitudinal and perpendicular ridges of the cell surface (O), whereas the pre-immunserum R1044 (N) does not reveal such a pattern. (Q–U) A dotted cortical pattern is visible when cells are stained with CRC-VI-3 specific antibodies (R, T). A dorsal view of such cells shows staining of the pores of t [file pone.0027111.s005.tif]

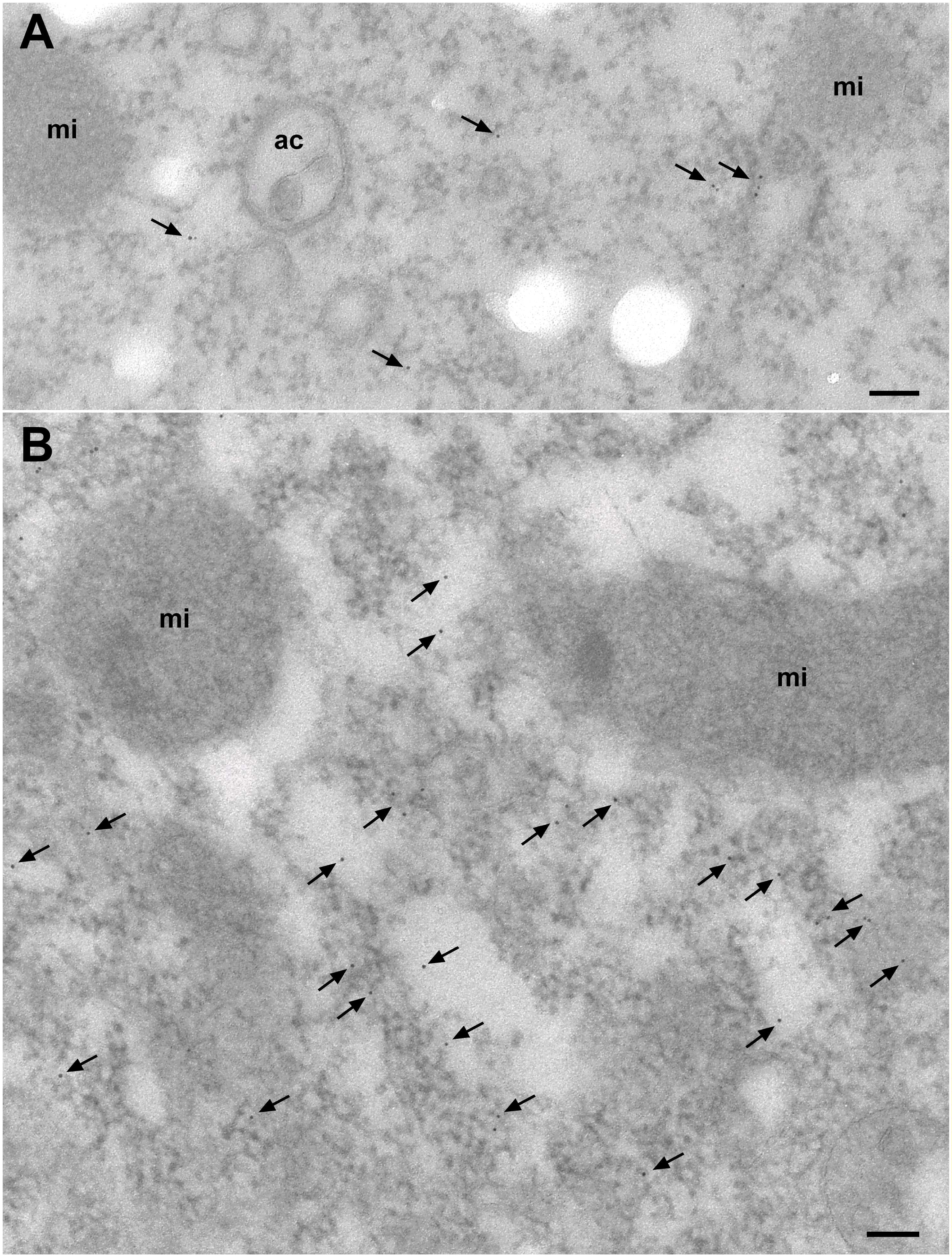

Supplement: Figure S6 — Immunogold EM localization of CRC-I-1. EM micrographs of ER-rich domains in peripheral (A) and central (B) regions of the cell. Immunogold labeling with CRC-I-1-specific antibodies is visible close to ER membranes (arrows), while mitochondria (mi) or acidosomes (ac) are not labeled. Bars = 0.1 µm. (TIF) [file pone.0027111.s006.tif]
