# Supplementary material for: Calcium-Release Channels in Paramecium. Genomic Expansion, Differential Positioning and Partial Transcriptional Elimination
Source: PLoS One. 2011 Nov 10;6(11):e27111. doi: 10.1371/journal.pone.0027111 (PMC3213138; doi:10.1371/journal.pone.0027111)
Supplement: Table S1 — Predictions of transmembrane regions in Paramecium CRC proteins. Results of transmembrane predictions using the TOPCONS algorithm (http://topcons.net/, [38]) obtained with full-length proteins (fl) or C-terminal regions as input queries. Putative transmembrane helices printed in red show slight deviations between the different queries, those printed in green represent results of one of the five topology predictions integrated in TOPCONS. The results retrieved for the CRC-III-1bΨ pseudogene are outlined in grey or bright red. Furthermore, full-length proteins were analyzed with TMHMM v.2.0 (http://www.cbs.dtu.dk/services/TMHMM/, [39]) and Kyte Doolitte hydrophobicity scales [40]. Additionally to the Paramecium CRC sequences, we analyzed three different metazoan IP3 and ryanodine receptors, which are from Mus musculus (MmIP3R type 1, Acc No: NP_034715.2; MmRyR type 1, Acc No: NP_033135), Drosophila melanogaster (DmIP3R, Acc No: BAA14399.1; DmRyR, Acc No: NP_033135) or Caenorhabditis elegans (CeITR1, Acc No: NP_001023173; CeRyR, Acc No: BAA08309). (DOC) [file pone.0027111.s007.doc]

**Table S1.** Predictions of transmembrane regions in *Paramecium* CRC proteins.

| **I** |  | **TMD1** | **TMD2** | **TMD3** | **TMD4** | **TMD5** | **pore** | **TMD6** |
| --- | --- | --- | --- | --- | --- | --- | --- | --- |
| CRC-I-1a (Sc9A*)_fl |  | L2557-F2577 | F2606-F2626 | Y2649-L2669 | Y2677-F2697 | Q2722-Y2742 |  | S2801-F2821 |
| R2453-L2972 |  | L2557-F2577 | F2606-F2626 | Y2649-L2669 | Y2677-F2697 | Q2722-Y2742 |  | S2801-F2821 |
| Seq |  | LWKDLAFLFTLLLNLFIIGSF | FLICGTIMACCSIFVVSFFLF | YWPIQLIYKIFKLLAVLFYIL | YLAYGALAVIGTVLHPFFFSF | QLGLTLVLFVILVYVYGLIAY | TLFCFLFTFDW  2: GGYL | SNIFLVIIMVNIVAGIIIDTF |
| TMHMM |  | 2561-2583 | 2604-2626 | 2646-2668 | 2675-2697 | 2724-2746 |  | 2801-2823 |
| Kyte Doolittle |  | 2560-2578 | 2606-2634 | 2653-2708 | 2653-2708 | 2726-2746 | 2756-2766  2775-3778 | 2803-2823 |
|  |  |  |  |  |  |  |  |  |
| CRC-I-1b (Sc26)_fl |  | L2562-F2582 | F2611-F2631 | Y2654-L2674 | Y2682-F2702 | Q2727-Y2747 |  | S2806-F2826 |
| R2458-L2977 |  | L2562-F2582 | F2611-F2631 | Y2654-L2674 | Y2682-F2702 | Q2727-Y2747 |  | S2806-F2826 |
| Seq |  | LWKDLAFLFTLLLNLFIIGSF | FLICGTIMACCSIFVVSFFLF | YWPIQLIYKIFKLLAVLFYIL | YLAYGALAVIGTVLHPFFFSF | QLALTLVLFIILVYVYGLIAY | TLFCFLFTFDW  2:GGYL | SNIFLVIIMVNIVAGIIIDTF |
| TMHMM |  | 2566-2588 | 2609-2631 | 2651-2673 | 2680-2702 | 2729-2751 |  | 2806-2828 |
| Kyte Doolittle |  | 2564-2583 | 2611-2640 | 2657-2712 | 2657-2712 | 2730-2750 | 2761-2771  2780-2783 | 2807-2827 |
|  |  |  |  |  |  |  |  |  |
| CRC-I-1c (Sc98*)_fl |  | L2565-F2585 | L2615-K2635 | Y2657-L2677 | Y2685-F2705 | Q2730-Y2750 |  | S2809-F2829 |
| E2460-L2980 |  | L2565-F2585 | L2615-K2635 | Y2657-L2677 | Y2685-F2705 | Q2730-Y2750 |  | S2809-F2829 |
| Seq |  | LWKDLAFLFTLLLNLFIIGSF | LICGTIMACCSIFVVSFFLFK | YWPIQLIYKIFKLLAVLFYIL | YLAYGALAVIGTVLHPFFFSF | QLGLTLVLFIILVYVYGLIAY | TLFCFLFTFDW  2: VGGYL | SNIFLVIIMVNIVAGIIIDTF |
| TMHMM |  | 2569-2586 | 2613-2635 | 2655-2677 | 2682-2704 | 2732-2754 |  | 2803-2825 |
| Kyte Doolittle |  | 2569-2585 | 2614-2641 | 2661-2716 | 2661-2716 | 2733-2753 | 2765-2774  2782-2786 | 2810-2830 |
|  |  |  |  |  |  |  |  |  |
| CRC-I-2a (Sc20*)_fl | S2456-L2476 | L2609-F2629 | I2657-L2677 |  | M2727-F2747 | Q2774-Y2794 |  | S2852-F2872 |
| I2504-N3010 |  | L2613-V2633 | T2654-S2674 |  | M2727-F2747 | L2775-W2795 | C2805-G2825 | S2852-F2872 |
| Seq |  | LAFFLTLFLNLFILFSFDGTV | TKTIIYIIGIVMTCLSLFVVS |  | MYYIAYGLFAILGTFYHPLFF | LILTFFLLFLLVYVFTIFAYW | CDTLLYCFMMNVEWTFRGSIG | SNITLGVIMLNIVAGIIIDTF |
| TMHMM | 2457-2479 | 2612-2634 | 2656-2678 | 2702-2721 | 2726-2748 | 2775-2797 | CDTLLYCFMMNV | 2854-2876 |
| Kyte-Doolittle | 2455-2476 | 2611-2631 | 2655-2688 | 2703-2726 | 2727-2756 | 2776-2796 | 2805-2816 | 2854-2873 |
|  |  |  |  |  |  |  |  |  |
| CRC-I-2b (Sc32)_fl |  | R2610-G2630 | T2653-S2673 | G2692-F2712 | I2729-H2749 | Q2773-Y2793 |  | V2850-T2870 |
| I2503-N3009 |  | L2608-F2628 | T2655-Y2675 |  | M2726-F2746 | L2774-W2794 | C2804-G2842 | V2850-T2870 |
| Seq |  | LWRELAFFLTLFLNLFILFSF | TIIYIIGIVMTGLSLFVVSFY |  | MYYIAYGLFAILGTFYHPLFF | LILTFFLLFLLVYVFTIFAYW | CDTLLYCFMMNVEWTFRGSIG | VSNIILGVIMLNIVAGIIIDT |
| TMHMM | 2407-2429 | 2611-2633 | 2655-2677 |  | 2725-2747 | 2774-2796 | DTLLYCFMMNV | 2850-2872 |
| Kyte-Doolittle | 2455-2476 | 2611-2631 | 2653-2683 | 2703-2725 | 2726-2756 | 2775-2795 | 2805-2815 | 2851-2873 |

| **II** |  | **TMD1** | **TMD2** | **TMD3** | **TMD4** | **TMD5** | **pore** | **TMD6** |
| --- | --- | --- | --- | --- | --- | --- | --- | --- |
| CRC-II-1a (Sc34_N1)_fl |  | L2460-F2480 | I2518-A2538 | M2560-Q2580 | G2591-V2611 | Q2632-F2652 |  | I2707-T2727 |
| E2360-L2890 |  | L2460-F2480 | I2518-A2538 | M2560-Q2580 | A2592-I2612 | L2633-Y2653 |  | I2707-T2727 |
| Seq |  | LWKDLAFYLTLIINTMIIASF | ISALGYAMMVCSLFVVLFVLA | MIKLLLNWLAKFFFVLFYCLQ | ALSVIGTLVHPFFFCFHLTVI | LVLTLLLIIIITYIFTLIAFY | DCKEVSICFLQIF | INNLLLVIIMVSIASGIIIDT |
| TMHMM |  |  | 2521-2543 | 2563-2585 | 2590-2612 | 2633-2655 |  | 2707-2729 |
| Kyte Doolittle |  | 2462-2481 | 2519-2548 | 2558-2628 | 2558-2628 | 2631-2658 | 2662-2674 | 2709-2729 |
|  |  |  |  |  |  |  |  |  |
| CRC-II-1b (Sc48_N2)_fl |  | L2458-F2478 | L2515-L2535 | M2558-Q2578 | G2589-V2609 | Q2630-F2650 |  | I2705-T2725 |
| E2358-L2888 |  | L2458-F2478 | L2515-L2535 | M2558-Q2578 | A2590-I2610 | Q2630-F2650 |  | I2705-T2725 |
| Seq |  | LWKDLAFYLTLIINTMIIASF | LISALGYAMMVCSLFVVLFVL | MIKLLINWLAKFFFVLFYCLQ | ALSVIGTLVHPFFFCFHLTVI | QLVLTLLLIIIITYIFTLIAF | DCKEVSICFLQIF | INNLLLVIIMVSIASGIIIDT |
| TMHMM |  |  | 2519-2541 | 2561-2583 | 2588-2610 | 2631-2653 |  | 2705-2727 |
| Kyte Doolittle |  | 2461-2479 | 2517-2546 | 2557-2625 | 2557-2625 | 2629-2656 | 2660-2672 | 2707-2727 |
|  |  |  |  |  |  |  |  |  |
| CRC-II-2 (Sc30)_fl |  | I2611-S2631 | R2667-K2687 | W2710-L2730 | L2738-H2758 | V2781-A2801 |  | N2861-F2881 |
| A2506-Q3017 |  | V2610-L2630 | R2667-K2687 | W2710-L2730 | L2738-H2758 | V2781-A2801 |  | N2862-G2882 |
| Seq |  | VILWKDLCFLLTLVLNVFIVL | RYCGIGMIVCSCFVVSFFILK | WIIRFFLKLYMVGFCLVKVLL | LAYGVLAFIATLVHPFFFAFH | VALGLTFILVLLMNYYFTLMA | DSLLVCFLSTFD | NILIQMISIQIFSGIIIDTFG |
| TMHMM |  | 2611-2633 | 2664-2686 | 2709-2731 | 2736-2758 | 2784-2806 |  |  |
| Kyte Doolittle |  | 2603-2633 | 2667-2690 | 2712-2767 | 2712-2767 | 2781-2808 | 2815-2826 | 2861-2882 |
|  |  |  |  |  |  |  |  |  |
| CRC-II-3 (Sc144)_fl |  | I2466-S2486 | F2531-L2551 | F2581-S2601 | G2603-E2623 | L2643-A2663 |  | L2721-T2741 |
| K2362-K2886 |  | I2466-S2486 | F2531-L2551 | F2581-S2608 | G2603-E2623 | L2643-A2663 |  | L2721-T2741 |
| Seq |  | ILWKDLAFILTLLLNLFIILS | FFICGIIMIVCSTFVVLFFLL | FNFSYSIARVLLNIEILYYLS | GTLAFLATFYHPFFFAFHLTE | LSLLLTFILIILFNYFFTLFA | CESLLYCFLETF | LYNIVIVIIMIQIFSGIIIDT |
| TMHMM | 2314-2333 | 2464-2486 | 2529-2551 | 2590-2612 |  | 2646-2668 |  | 2720-2742 |
| Kyte Doolittle |  | 2450-2488 | 2530-2556 | 2571-2629 | 2571-2629 | 2644-2669 | (2675)2678-2686 | 2722-2743 |
|  |  |  |  |  |  |  |  |  |
| CRC-II-4 (Sc1)_fl |  | L2498-F2518 | I2558-A2578 | I2600-L2620 | S2624-L2644 | I2668-Y2688 |  | D2745-I2765 |
| S2396-Q2910 |  | L2598-F2518 | I2558-A2578 | I2600-L2620 | S2624-L2644 | I2668-Y2688 |  | N2746-D2766 |
| Seq |  | LYRDITFILALTINVMILFVF | IQTLGIIVIVLSMLIVLFFLA | IMYIRRLLMTVVYLVTDFYVL | SYGFTAFLGTLYHPFFFAFHL | ILFTLFLFIVLMYVFSLVAYY | CYSTFQCLLTAV  2: LGGFLTPS | NIYFILLMIIMINIVSGIIID |
| TMHMM |  | 2496-2518 | 2563-2585 | 2600-2622 | 2627-2649 | 2669-2691 |  | 2747-2769 |
| Kyte Doolittle |  | 2501-2519 | 2556-2589 | 2600-2661 | 2600-2661 | 2670-2690 | 2699-2710  2720-2727 | 2740-2769 |

| CRC-II-5 (Sc79)_fl |  | L2452-Y2472 | I2503-S2523 | R2550-Y2570 | T2572-V2592 | K2611-S2631 |  | D2690-I2710 |
| --- | --- | --- | --- | --- | --- | --- | --- | --- |
| Q2353-D2847 |  | L2452-Y2472 | I2503-S2523 | R2550-Y2570 | T2572-V2592 | K2611-S2631 |  | D2690-I2710 |
| Seq |  | LLRDIEFIIALLINIFIFLFY | IDALGILSIVLSILIVAFFLS | RLLKTVYYLLSDFFALYYIMY | TAAVLGRFVHNFFFSFHLFEV | KMILYTGLLLLIFMFVFTVFS | DSMWICFLSTLD | DNFFNILVIIVMLNIVAGIII |
| TMHMM |  | 2455-2472 | 2505-2522 | 2555-2577 | 2584-2603 | 2613-2635 |  | 2692-2714 |
| Kyte Doolittle |  | 2454-2472 | 2504-2527 | 2546-2605 | 2546-2605 | 2615-2633 | 2645-2657 | 2683-2713 |
|  |  |  |  |  |  |  |  |  |
| **III** |  | **TMD1** | **TMD2** | **TMD3** | **TMD4** | **TMD5** | **pore** | **TMD6** |
| CRC-III-1a (Sc86*)_fl |  | L2188-Y2208 | L2248-I2268 | I2292-I2312 | I2322-L2342 | F2359-V2379 |  | I2447-S2467 |
| I2085-F2598 |  | L2188-Y2208 | L2248-I2268 | I2292-I2312 | F2320-L2340 | F2359-V2379 |  | I2448-Y2468 |
| Seq |  | LWMQLAFYNGFFINLIIILSY | LMMLAYLNLAINFMVLFFYLI | ISGLIKVIISIIVLVIDFDII | FNIIGITIHPFFLAFHLIHIL | FIGLWLTIILFEYWVALISYV | LWQCVIVTFDW | INIIIKLCIINVLLAVIIISY |
| TMHMM |  | 2188-2210 | 2246-2268 | 2283-2305 | 2318-2340 | 2360-2382 |  | 2446-2468 |
| Kyte Doolittle |  | 2191-2211 | 2249-2272 | 2286-2355 | 2286-2355 | 2356-2386 | 2397-2407 | 2447-2470 |
|  |  |  |  |  |  |  |  |  |
| CRC-III-1bΨ (Sc158*) |  | L2188-Y2208 | L2248-I2268 | M2291-I2311 | I2322-L2342 | F2359-V2379 |  | I2447-S2467 |
| E1841-F2598 (V2369) |  | L2188-Y2208 | L2248-I2268 | I2292-I2312 | F2320-L2340 | K2358-Y2378 |  | I2448-Y2468 |
| Seq |  | LWMQLAFYNGFLINLIIILSY | LMILAYLNLAINFMVLFFYLI | ISGLIKVVISIIVLVIDFDII | FNIIGITIHPFFLAFHLIHIL | KFIGLWLIIVLFEYWVALISY | WQCVYVTFDW  2: VGGMFVE | INVIVKLCVINVLLAVIIISY |
| TMHMM |  | 2188-2210 | 2246-2268 | 2283-2305 | 2318-2340 | 2360-2382 |  | 2446-2468 |
| Kyte Doolittle | 2149-2160 | 2191-2211 | 2248-2275 | 2286-2386 | 2286-2386 | 2286-2386 | 2397-2407  2415-2421 | 2447-2470 |
|  |  |  |  |  |  |  |  |  |
| CRC-III-2 (Sc35*)_fl |  | L2291-Y2311 | L2377-A2397 | G2418-L2438 | F2443-A2463 | I2484-Y2504 |  | D2581-I2601 |
| E2190-I2738 |  | L2291-Y2311 | L2377-A2397 | G2418-L2438 | F2443-A2463 | I2484-Y2504 |  | D2581-I2601 |
| Seq |  | LWEQLAFVTNVIINLVILCSY | LIVILGYLNLGFSLLVITFFA | GCIERTIMSFVMIVRSFINCL | FAYFCGYMACIVVGLVIHPFA | IWISKIQMGLSFLVFLLVEYY | MWRCFLQTF  2: IGDY | DVLFKFILVFLIINMVAGIII |
| TMHMM |  |  | 2376-2398 | 2445-2467 |  | 2492-2514 |  | 2582-2604 |
| Kyte Doolittle | 2279-2290 | 2294-2319 | 2375-2418 | 2422-2513 | 2422-2513 | 2422-2513 | 2525-2533  2543-2546 | 2578-2606 |
|  |  |  |  |  |  |  |  |  |
| CRC-III-3 (Sc3B)_fl |  | L2601-Y2621 | I2660-L2680 | F2700-Q2720 | Y2730-M2750 | E2771-W2791 |  | F2878-F2898 |
| E2497-E3036 |  | W2602-S2622 | I2660-L2680 | F2700-Q2720 | A2729-L2749 | E2771-W2791 |  | F2878-F2898 |
| Seq |  | WELCLFLTTIAINLIILFSYS | ILILGILDLAFSSLIVFFFAL | FFKIIIRLIKNSLVSIFVLLQ | AYILAGIVGLAAHPFFFCVHL | ELGLALMLLIIIEYYFNILAW | WRCLITTFD  2: FTGSIG | FNIMLVFILLNMIQGIIIDTF |
| TMHMM | 2400-2417 | 2604-2626 | 2658-2680 | 2687-2709 | 2724-2746 | 2772-2794 |  | 2880-2902 |
| Kyte Doolittle |  | 2603-2624 | 2657-2684 | 2688-2754 | 2688-2754 | 2772-2795 | 2813-2821  2826-2831 | 2876-2899 |

| CRC-III-4a (Sc49)_fl |  | L2370-Y2390 | L2428-L2448 | V2469-Q2489 | Y2499-M2519 | I2541-V2561 |  | N2664-D2684 |
| --- | --- | --- | --- | --- | --- | --- | --- | --- |
| Q2266-G2835 |  | L2370-Y2390 | L2428-L2448 | V2469-Q2489 | Y2499-M2519 | I2541-V2561 |  | N2664-D2684 |
| Seq |  | LWENCAFVTTLVINLIVLLSY | LIQGLGITMLVFSSLIVFFFL | VMKMIWEVSVRGIKSLFILLQ | YIIAGIVGLTVHPFFFAFHLM | IGLALLLLAVVEYYVGILGFV | MWQCIFISFDLTFKFTGALGSGI | NAVNIVLVMIMLNMIQGIIVD |
| TMHMM |  |  | 2427-2449 |  | 2493-2515 | 2541-2563 |  | 2660-2682 |
| Kyte Doolittle |  | 2374-2392 | 2426-2453 | 2472-2478  2481-2492 | 2496-2523 | 2543-2567 | 2593-2615 | 2662-2687 |
|  |  |  |  |  |  |  |  |  |
| CRC-III-4b (Sc17)_fl |  | L2357-Y2377 | L2415-L2435 | V2456-Q2476 | Y2486-M2506 | G2529-I2549 |  | N2651-D2671 |
| Q2253-A2822 |  | L2357-Y2377 | L2415-L2435 | V2456-Q2476 | Y2486-M2506 | G2529-I2549 |  | N2651-D2671 |
| Seq |  | LWENCAFVTTLVINLIVLLSY | LIQGLGITMLVFSSLIVFFFL | VMKMIWDVSIRGIKSLIILLQ | YIIAGIVGLTVHPFFFAFHLM | GLALLLLAVIEYYVGILGFVI | LWQCIFMSFDLTFKFTGALGQG | NAVNIVLVMIMLNMIQGIIVD |
| TMHMM |  |  | 2414-2436 |  | 2480-2502 | 2528-2550 |  | 2652-2674 |
| Kyte Doolittle |  | 2361-2378 | 2411-2440 | 2459-2479 | 2482-2510 | 2530-2555 | 2580-2601 | 2650-2675 |
|  |  |  |  |  |  |  |  |  |
| CRC-III-4c (Sc37*)_fl | Y2170-G2190 | W2370-S2390 | M2427-L2447 |  | S2496-H2516 | I2540-V2560 |  | V2665-F2685 |
| A2121-E2834 | Y2170-G2190 | W2370-S2390 | M2427-L2447 |  | S2496-H2516 | I2540-V2560 |  | V2665-F2685 |
|  | YYEFIIENGFFILLLMQQFLG | WENLAFVTTLVINFIVLLSYS | MIQGLGITMLVFSSLIVFFFL | L2468-Q2488  PRODIV (fl) | SIYIIAGIVGLTVHPFFFAFH | IGLALLLLAVIEYYVGILGFV | WQCIFMSFDLTFKFTGALGSGI | VNIVLVMIMLNMIQGIIVDTF |
| Kyte Doolittle |  | 2373-2390 | 2426-2452 | 2472-2491 | 2495-2522 | 2542-2567 | 2593-2614 | 2660-2686 |
| TMHMM |  |  | 2426-2448 |  | 2492-2514 | 2540-2562 |  | 2664-2686 |
|  |  |  |  |  |  |  |  |  |
| **IV** |  | **TMD1** | **TMD2** | **TMD3** | **TMD4** | **TMD5** | **pore** | **TMD6** |
| CRC-IV-1a (Sc138_C1) |  | I2603-I2623 |  |  | F2718-Y2738 | K2764-A2784 |  | F2862-F2882 |
| N2509-H2997 |  | I2603-I2623 | K2635-I2655 | V2693-F2713 | F2718-Y2738 | Q2765-Y2785 |  | V2860-D2880 |
| Seq |  | IVYLRNVCIILALLLNILITI | KAIAIVNCILYGLIMLTWVFI | VIYPFQITLLQAFLIIKRMLF | FYWFLLLVINLLGLLYNKLFY | QLLMTALLGVLIMYLYSLVAY | VCYSAFQCLVYVIHQG / IGDAL | VTFFILINIIWLNIIFGIIID |
| TMHMM |  | 2602-2621 | 2633-2655 | 2694-2713 | 2720-2742 | 2766-2785 |  | 2860-2882 |
| Kyte Doolittle |  | 2606-2624 | 2634-2657 | 2695-2713 | 2716-2750 | 2768-2786 | 2814-2829  2836-2840 | 2859-2883 |
|  |  |  |  |  |  |  |  |  |
| CRC-IV-1b (Sc80_C2) |  | I2607-I2627 | A2640-R2660 | P2700-Y2720 | F2722-Y2742 | Q2769-Y2789 |  | F2865-F2885 |
| S2513-H3000 |  | I2607-I2627 | A2640-R2660 | V2697-F2717 | Y2720-L2740 | Q2769-Y2789 |  | F2865-F2885 |
| Seq |  | IVYLRNVCIILALLLNILITI | AIAILDCILYGLIMLIWIFIR | VIYPFQITLLQTFHIIKRMLF | YTFYWFLLLVVNLLGLLYNKL | QLLMTALLGVLIMYLYSLVAY | VCYSAFQCLVFVIHQG / GGGIGDVL | FFILINIIWLNIIFGIIIDTF |
| TMHMM |  | 2606-2625 | 2637-2659 |  | 2724-2746 | 2767-2789 |  | 2860-2882 |
| Kyte Doolittle |  | 2610-2628 | 2638-2662 | 2700-2711  2713-2716 | 2719-2753 | 2772-2788 | 2817-2832  2836-2843 | 2863-2886 |

| CRC-IV-2 (Sc9B)_fl |  | T2298-P2318 |  |  | L2445-L2465 | N2488-K2508 |  | D2568-V2588 |
| --- | --- | --- | --- | --- | --- | --- | --- | --- |
| SCAMPI-msa |  | T2298-P2318 | N2350-I2370 | L2428-F2448 | I2450-K2470 | N2488-K2508 |  | D2568-V2588 |
| A2197-Q2691 |  | T2298-P2318 | L2349-A2369 | L2428-F2448 | I2450-K2470 | N2488-K2508 |  | D2568-V2588 |
| Seq |  | TLKDVSYILCVVIVLFFILMP | LNNIITVIQLILNLIIIIFCA | LRKIIVLLLIDFDNLYNLIIF | ITAIAFFNNYVYAILLLDIVK | NLLIFGLLGLIGLVLYGFLIK | (LGYSI)AHVINFG | DLTFFIIFNILFLQMIFGIIV |
| TMHMM |  | 2302-2324 | 2345-2367 |  | 2446-2468 | 2489-2511 |  | 2566-2588 |
| Kyte Doolittle |  | 2304-2321 | 2344-2376 | 2432-2472 | 2432-2472 | 2491-2513 | (2526) 2529-2537 | 2570-2591 |
|  |  |  |  |  |  |  |  |  |
| CRC-IV-3a (Sc62)_fl |  | L2722-H2742 | N2773-I2793 | I2814-I2834 | I2868-L2888 | N2911-F2931 |  | D2991-L3011 |
| L2609-Q3118 |  | L2722-H2742 |  |  | I2868-L2888 | N2911-F2931 |  | F2992-D3012 |
| Seq |  | LLKDISFLLCLIFVILLIFMH |  |  | ICIFGLTVYAFFNPYIYAVLL | NLAIFSFLGLIGLLVYAIIAF |  | FTFFIIFNILFIQIIFGIILD |
| L2281-Q3118 |  | R2721-M2741 | N2773-I2793 | E2843-D2863 | I2868-L2888 | N2911-F2931 |  | F2994-F3014 |
|  |  | RLLKDISFLLCLIFVILLIFM | NNIITIVQLVLNLIIVFFCAI | EDKVNHNPIKQLILVIFFDFD | ICIFGLTVYAFFNPYIYAVLL | NLAIFSFLGLIGLLVYAIIAF | GQTFILAVTSTIN | FFIIFNILFIQIIFGIILDTF |
| TMHMM |  | 2718-2740 | 2771-2793 |  | 2870-2892 | 2912-2934 |  | 2989-3011 |
| Kyte Doolittle |  | 2715-2745 | 2767-2798 | 2854-2893 | 2854-2893 | 2913-2935 | 2946-2958 | 2992-3015 |
|  |  |  |  |  |  |  |  |  |
| CRC-IV-3b (Sc24)_fl | V2252-I2272 | L2732-D2752 | I2784-R2804 |  | I2877-L2897 | L2921-S2941 |  | F3003-F3023 |
| SCAMPI-msa |  | L2732-D2752 | I2784-R2804 | 2864-2879 | 2887-2907 | L2921-S2941 |  | F3003-F3023 |
| S2617-Q3127 |  | L2731-H2751 |  |  | I2877-L2897 | L2921-S2941 |  | F3003-F3023 |
| Seq |  | LLKDVSFLLCLIIVVLLIFMH |  |  | ICIFGLTVYAFFNPYIYAILL | LAIFSFLGLIGLLIYAIIAFS | GQTFILAVTSTIN | FFIIFNILFIQIIFGIILDTF |
| L2287-Q3127 |  | L2731-H2751 | N2782-I2802 | I2823-I2843 | I2877-L2897 | N2920-F2940 |  | F3003-F3023 |
| Seq |  | LLKDVSFLLCLIIVVLLIFMH | NNIITIVQLVLNLIIVFFCAI | ILKKEAGFQISWLTMKYYSLI | ICIFGLTVYAFFNPYIYAILL | NLAIFSFLGLIGLLIYAIIAF |  | FFIIFNILFIQIIFGIILDTF |
| TMHMM |  | 2727-2749 | 2780-2802 |  | 2879-2901 | 2921-2941 |  | 2998-3020 |
| Kyte Doolittle |  | 2724-2753 | 2776-2806 | 2862-2902 | 2862-2902 | 2922-2944 | 2955-2967 | 3001-3024 |
|  |  |  |  |  |  |  |  |  |
| CRC-IV-4a (Sc6)_fl |  | L2728-H2748 | N2780-E2800 | I2820-I2840 | I2874-L2894 | L2918-S2938 |  | F2998-F3018 |
| N2615-S3125 |  | L2728-H2748 |  |  | I2874-L2894 | L2918-S2938 |  | F2998-F3018 |
| Seq |  | LLKDLSFILCLVIVILLIFMH |  |  | ICIFGLTVYAFFNPYIYAVLL | LAIFSFLGFIGLLIYAIIAFS | GQTFILAVTSTINFGLRSG | FFIIFNILFIQIIFGIILDTF |
| N2281-S3125 |  | L2728-H2748 | N2779-I2799 | A2825-S2845 | I2874-L2894 | L2918-S2938 |  | F2998-F3018 |
| Seq |  | LLKDLSFILCLVIVILLIFMH | NNIITIIQLVLNLIIVFFCAI | AGFSIAWLTMKYYTIIGYFES | ICIFGLTVYAFFNPYIYAVLL | LAIFSFLGFIGLLIYAIIAFS |  | FFIIFNILFIQIIFGIILDTF |
| TMHMM |  | 2724-2746 | 2777-2799 |  | 2876-2898 | 2918-2940 | 2950-2972 | 2993-3015 |
| Kyte Doolittle |  | 2718-2755 | 2778-2803 | 2828-2843 | 2859-2898 | 2920-2941 | 2952-2969 | 2996-3019 |

| CRC-IV-4b (Sc3A)_fl |  | L2734-D2754 | N2785-E2805 |  | I2879-L2899 | L2923-S2943 |  | 3000-L3020 |
| --- | --- | --- | --- | --- | --- | --- | --- | --- |
| SCAMPI-msa |  | 2733-2753 | 2784-2804 | 2862-2882 | 2884-2904 | 2923-2943 |  | 3003-3023 |
| N2620-S3130 |  | L2733-H2753 |  |  | I2879-L2899 | L2923-S2943 |  | F3003-F3023 |
| Seq |  | LLKDVSFILCLVIVILLIFMH |  |  | IWMFGLTVYAFFNPYIYAVLL | LAIFSFLGFIGLLIYAIIAFS |  | FFIIFNILFIQIIFGIILDTF |
| D2321-S3130 |  | L2734-D2754 | N2784-I2804 | I2866-V2886 | A2888-D2908 | L2923-S2943 |  | F3003-F3023 |
| Seq |  | LKDVSFILCLVIVILLIFMHD | NNIITIIQLVLNLIIVFFCAI | ILVIFFDFDNFYNIWMFGLTV | AFFNPYIYAVLLLDIIKRSED | LAIFSFLGFIGLLIYAIIAFS | GQTFILAVTSTINFGLRS | FFIIFNILFIQIIFGIILDTF |
| TMHMM |  | 2729-2751 | 2782-2804 |  | 2880-2902 | 2923-2945 | 2955-2977 | 2998-3020 |
| Kyte Doolittle |  | 2722-2760 | 2784-2809 | 2865-2874 | 2880-2904 | 2924-2946 | 2957-2974 | 3001-3024 |
|  |  |  |  |  |  |  |  |  |
| **V** |  | **TMD1** | **TMD2** | **TMD3** | **TMD4** | **TMD5** | **pore** | **TMD6** |
| CRC-V-1 (Sc2)_fl |  | V2310-Y2330 | I2351-F2371 | T2399-T2419 |  | Q2448-F2468 |  | D2515-I2535 |
| N2216-G2654 |  | V2310-Y2330 | I2351-F2371 | L2388-L2408 | S2410-S2430 | K2447-S2467 |  | F2518-F2538 |
| Seq |  | VMQKLSSVLALLINLSMIFFY | IKMLSVSQLFSQIILFIMVSF | LSLLLVIIQEDTCLLLFLLVL | SFFGAFINSTVFVIHLVEIFS | KQLLVVAFLGVLFVFAFSVIS | DSLITCMITLITSGVIGNS | FTVFFGLLFTNIVQGIMIDTF |
| TMHMM |  | 2319-2341 | 2354-2376 |  | 2407-2429 | 2450-2472 | 2487-2506 | 2513-2535 |
| Kyte Doolittle |  | 2314-2338 | 2342-2376 | 2390-2443 | 2390-2443 | 2448-2473 | 2485-2503 | 2513-2539 |
|  |  |  |  |  |  |  |  |  |
| CRC-V-2 (Sc3C)_fl |  | L2329-L2349 | L2368-V2388 | I2411-Y2431 | V2433-L2453 | L2468-N2488 |  | D2534-I2554 |
| F2235-I2674 |  | L2329-Y2349 | I2370-L2390 | I2411-Y2431 | L2434-L2454 | L2468-N2488 |  | D2534-I2554 |
| Seq |  | LLQNLASTFAVLINLAMIFFY | INLLSLGQLFSQIALFIMVSL | IVFFKEETCLILLFLVIISFY | FFNSNFYVIHLVEIFSRNSLL | LFVVGLLGILFVFAFSVISFN | NSLITCMITLITSGVIGNS | DMLFTVFFGLLFTNIIQGIMI |
| TMHMM |  | 2334-2353 |  |  | 2420-2442 | 2469-2491 | 2504-2526 | 2536-2558 |
| Kyte Doolittle |  | 2331-2353 | 2368-2395 | 2409-2446/51 (2457-2462) |  | 2467-2491 | 2504-2522 | 2532-2557 |
|  |  |  |  |  |  |  |  |  |
| CRC-V-3 (Sc38)_fl |  | V2262-Y2282 | I2302-L2322 | F2410-F2430 |  | A2456-T2476 | L2498-W2518 | D2526-I2546 |
| F2180-N2707 |  | V2262-Y2282 | I2302-L2322 | I2411-F2431 |  | L2459-D2479 | C2495-M2515 | D2526-I2546 |
| Seq |  | VLRHLSAFFSISVNILMILFY | IINVLSFCQLFTTLVYYICYL | IQITFYLVFSILGTFYKSYFF |  | LIVVSLLGVLFIYVFSFTSFD | CETLISCMITLVTSGVIGTSM | DTLYFVFFALLFTNIVSGIMI |
| TMHMM |  |  | 2302-2324 |  | 2414-2436 | 2456-2478 | 2498-2515 | 2530-2552 |
| Kyte-Doolittle |  | 2264-2282 | 2300-2322 | 2413-2453 | 2413-2453 | 2459-2477 | T2497-S2514 | 2525-2550 |
|  |  |  |  |  |  |  | TLISCMITLVTSGVIGTS |  |

| CRC-V-4a (Sc96)_fl |  | L2223-Y2243, | L2264-I2284 |  | H2327-L2347, | Q2372-F2392 |  | D2439-T2459 |
| --- | --- | --- | --- | --- | --- | --- | --- | --- |
| N2129-E2583 |  | L2223-Y2243 | L2264-I2284 | H2305-M2325 | T2331-Y2351 | Q2372-F2392 |  | D2439-T2459 |
| Seq |  | LLINISQLLSLIINIFMIFAY | LFILSILQFGFSLCACTFYVI | HTYVISKISNLIIVFKSEDFM | TAIAFIGLISNTYYFSLHLFY | QLSLVALLGVLFQFVFSIVGF | SLISCMITLMTSGVIGSS | DTVYFVFFALLFTNIISGIMT |
| TMHMM |  | 2223-2245 | 2262-2284 |  | 2330-2352 | 2373-2392 | 2407-2429 | 2441-2463 |
| Kyte Doolittle |  | 2221-2246 | 2262-2246 | 2304-2322 | 2327-2343  2346-2367 | 2373-2394 | 2411-2427 | 2437-2462 |
|  |  |  |  |  |  |  |  |  |
| CRC-V-4b (Sc106*)_fl |  | Q2228-A2248 | L2270-I2290 |  | F2336-F2356 | Q2378-F2398 |  | D2445-T2465 |
| N2135-E2589 |  | L2229-Y2249 | L2270-I2290 | H2311-L2331 | A2338-L2358 | Q2378-F2398 |  | D2445-T2465 |
| Seq |  | LLINISQLLSLFINIFMIYAY | LFILSLLQFSFSLCACTFYII | HSYIIQKISNFIIVFKGEDFL | AIAFLGLISNTYYFSLHLFYL | QLSLVALLGVLFQFVFSIVGF | SLISCMITLMTSGVIGSS | DTVYFVFFALLFTNIISGIMT |
| TMHMM |  | 2229-2251 | 2268-2290 |  | 2336-2358 | 2379-2398 |  | 2442-2464 |
| Kyte Doolittle |  | 2227-2253 | 2269-2294 | 2311-2328 | 2333-2349  2352-2372 | 2379-2400 | 2416-2433 | 2444-2467 |
|  |  |  |  |  |  |  |  |  |
| **VI** |  | **TMD1** | **TMD2** | **TMD3** | **TMD4** | **TMD5** | **pore** | **TMD6** |
| CRC-VI-1 (Sc44B)_fl |  | Q2353-L2373 | L2387-I2407 | F2480-I2500 | G2502-V2522 | I2534-L2554 |  | E2618-L2638 |
| T2238-S2774 |  | Q2353-L2373 | L2387-I2407 | R2476-T2496 | I2498-W2518 | I2534-L2554 |  | F2619-D2539 |
| Seq |  | QEIWKQIIFYVIVMQNIMILL | LLALNIIQIILQFVCFFIFII | RLVLFKYIFFDYEMIYFLIFT | IAISGLFTKTLLALLLLDVFW | IIQILLTLALFFILQYYYSLL | (CQ)SLLQCFSFILDV(T)  2: AGYVAASNG | FCYYFFVISLTYSIFTGLILD |
| TMHMM |  |  | 2385-2407 |  | 2491-2513 | 2533-2555 |  | 2613-2635 |
| Kyte Doolittle |  | 2358-2374 | 2384-2411 | 2476-2558  2485/6 charged | 2476-2558  2532/3 charged | 2476-2558 | (2569)2571-2582(2583)  2591-2599 | 2611-2642 |
|  |  |  |  |  |  |  |  |  |
| CRC-VI-2a (Sc18)_fl |  | Q2365-F2385 | L2399-V2419 | S2488-T2508 | L2510-W2530 | S2546-I2566 |  | N2621-G2641 |
| E2261-G2773 |  | K2366-S2386 | L2399-V2419 | S2488-T2508 | L2510-W2530 | S2564-I2566 |  | E2624-I2644 |
| Seq |  | KLWKQIIFLVIVLENLMILFS | LFGLTITQIILQFLCFFIFIV | SLTLYRIILLDYEMIYFLIFT | LAFIGLYYNVVLALLLLDVFW | SGSILLVLSLYIIMQYYYSLI | FLQCFSFILDAT  2: SVGYIA | EFVYVFVVISLLYSIITGIII |
| TMHMM |  |  | 2397-2419 |  | 2502-2524 | 2549-2571 |  | 2622-2644 |
| Kyte Doolittle |  | 2371-2389 | 2395-2423 | 2486-2543 | 2486-2543 | 2547-2570 | 2582-2593  2600-2605 | 2622-2649 |
|  |  |  |  |  |  |  |  |  |
| CRC-VI-2b (Sc44A*)_fl |  | Q2366-F2386 |  |  | L2506-L2526 | S2547-I2567 |  | E2625-I2645 |
| SCAMPI-msa |  | 2366-2386 | 2400-2420 | 2489-2509 | 2511-2531 | 2550-2570 |  | 2628-2648 |
| E2262-G2774 |  | Q2366-F2386 | L2400-V2420 | Y2489-T2509 | L2511-W2531 | S2547-I2567 |  | E2625-I2645 |
| Seq |  | QKLWKQIIFLVIVLENLMILF | LFGLTITQIILQFLCFFIFIV | YLTLYKCILLDYEMIYFLIFT | LAFIGLYVNIVLALLLLDVFW | SGSILLVLSLYIIMQYYYSLI | LLQCFSFILDVT  2: SVGFVSSS | EFVYVFVVISLLYSIITGIII |
| TMHMM |  |  | 2398-2420 |  | 2503-2525 | 2550-2572 |  | 2623-2645 |
| Kyte Doolittle |  | 2372-2389 | 2396-2424 | 2488-2544 | 2488-2544 | 2547-2571 | 2583-2594  2601-2608 | 2624-2649 |
|  |  |  |  |  |  |  |  |  |
| CRC-VI-3 (Sc134*)_fl |  |  | L1714-F1734 | Q1764-E1784 |  | V1805-I1825 |  | S1895-M1915 |
| OCTOPUS |  | 1672-1692 | 1715-1735 |  | 1771-1791 | 1812-1832 |  | 1905-1925 |
| Q1441-L2011 |  | L1672-F1692 | L1714-F1734 | F1759-V1779 | G1781-T1801 | Q1815-F1835 |  | F1905-D1925 |
| Seq |  | LATSITALNIIVLLLISNTAF | LTLCQILLIFMSLLALICQLF | MFKILQRRDIFQFFLHFVLSV | GTFEPFCFYLQSFCFIYISQT | QFLGIFSVLLIILNAYSYIGF |  | FSFFIIVTLIMINIINGIIID |
| E1161-L2011 |  | Q1671-A1691 | L1712-Q1732 | Y1753-L1773 | V1776-I1796 | F1816-R1836 |  | F1905-D1925 |
| Seq |  | QLATSITALNIIVLLLISNTA | LALTLCQILLIFMSLLALICQ | YNTFVIMFKILQRRDIFQFFL | VLSVLGTFEPFCFYLQSFCFI | FLGIFSVLLIILNAYSYIGFR | TPAHCFISLVYFGL | FSFFIIVTLIMINIINGIIID |
| TMHMM |  | 1670-1692 | 1712-1734 |  | 1768-1790 | 1816-1835 |  | 1902-1924 |
| Kyte-Doolittle |  | 1675-1692 | 1714-1742 | 1755-1764 | 1771-1810 | 1817-1832 | 1863-1876 | 1903-1928 |
|  |  |  |  |  |  |  |  |  |
|  |  |  |  |  |  |  |  |  |
| **Metazoan IP_3_Rs** |  | **TMD1** | **TMD2** | **TMD3** | **TMD4** | **TMD5** | **pore** | **TMD6** |
| *Mm*IP_3_R1_fl |  | 2274-2294 | 2307-2327 | 2339-2359 | 2393-2413 | 2439-2459 |  | 2570-2590 |
| Seq |  | FWSSISFNLAVLMNLLVAFFY | HWSGLLWTAMLISLAIVIALP | TILRLIFSVGLQPTLFLLGAF | YHLLYLLICAMGLFVHEFFYS | SIILTAVLALILVYLFSIVGY | LLMCIVTVLSHGL | LLFFFMVIIIVLNLIFGVIID |
| E2001-A2749 |  | 2274-2294 | 2307-2327 | 2339-2359 | 2393-2413 | 2439-2459 |  | 2569-2589 |
| Seq |  | FWSSISFNLAVLMNLLVAFFY | HWSGLLWTAMLISLAIVIALP | TILRLIFSVGLQPTLFLLGAF | YHLLYLLICAMGLFVHEFFYS | SIILTAVLALILVYLFSIVGY |  | DLLFFFMVIIIVLNLIFGVII |
| Kyte Doolittle |  | 2276-2297 | 2312-2129 | 2334-2377 | 2386-2420 | 2441-2467 | 2530-2542 | 2564-2593 |
| TMHMM |  | 2274 2296 | 2309 2326 | 2352 2374 | 2395 2417 | 2440 2462 |  | 2570 2592 |
|  |  |  |  |  |  |  |  |  |
| *Dm*IP_3_R_fl |  | 2365-2385 | 2397-2417 | 2438-2458 | 2484-2504 | 2527-2547 |  | 2657-2677 |
| Seq |  | LWSNILFNCVVVINMIVAFFY | HISLLFWIITIFSLVIVLALP | GPESTLCLLGVVTVTLKSVHI | ALYSVLLLRLIFHPFFYSLLL | SIVLTAVLALILVYLFSIIGY | LVMCIVTTLNQ  2: GDILR | LLFFFIVIIIVLNLIFGVIID |
| G2001-Q2833 |  | 2365-2385 | 2397-2417 | 2438-2458 | 2484-2504 | 2529-2549 |  | 2657-2677 |
| Seq |  | LWSNILFNCVVVINMIVAFFY | HISLLFWIITIFSLVIVLALP | GPESTLCLLGVVTVTLKSVHI | ALYSVLLLRLIFHPFFYSLLL | VLTAVLALILVYLFSIIGYMF | LVMCIVTTLNQ  2: GDILR | LLFFFIVIIIVLNLIFGVIID |
| Kyte Doolittle |  | 2355-2388 | 2396-2419 | 2425-2464 | 2473-2508 | 2429-2550 | 2617-2627  2636-2640 | 2649-2681 |
| TMHMM |  | 2365 2387 | 2394 2416 | 2429 2451 | 2472 2494 | 2528 2550 |  | 2657 2679 |
|  |  |  |  |  |  |  |  |  |
| *Ce*ITR1_fl |  | 2400-2420 | 2431-2451 | 2469-2489 | 2524-2544 | 2570-2590 |  | 2688-2708 |
| L2001-R2846 |  | 2400-2420 | 2431-2451 | 2469-2489 | 2524-2544 | 2570-2590 |  | 2688-2708 |
| Seq |  | LWTRLSFHFAFIVNALVARYY | SLGNLYSWFAVFSSFLLAHYL | LCFLLLSSIGVTLTLYIFGIL | LLVYLFICILGLLVHPMIYCI | IVWTGLLALILLYFFSILGFL | TLWMCILQTGY | MTFFVVLIVIVLNLIFGVIID |
| Kyte Doolittle |  | 2394-2420 | 2432-2450 | 2461-2504 | 2514-2554 | 2572-2592 | 2647-2657 | 2688-2711 |
| TMHMM |  |  | 2429 2451 | 2464 2486 | 2525 2547 | 2570 2592 |  | 2685 2707 |

|  |  |  |  |  |  |  |  |  |  |
| --- | --- | --- | --- | --- | --- | --- | --- | --- | --- |
| **Metazoan RyRs** | **TMD X-4** | **TMD X-4'** | **TMD X-3** | **TMD X-2** | **TMD X-1'** | **TMD X-1** | **TMD X** | **pore** | **TMD X+1** |
| *Mm*RyR_fl | 4324-4344 | 4347-4367 | 4557-4577 | 4642-4662 |  | 4787-4807 | 4837-4857 | 4873-4893 | 4915-4935 |
| Seq | EAATAVAALLWALVTRAGGAG | AAAGALRLLWGSLFGGGLVDS | FYTLRFLALFLAFAINFILLF | RCLSLLHTLVAFLCIIGYNCL |  | FLYLGWYMVMSLLGHYNNFFF | MTVGLLAVVVYLYTVVAFNFF | KCDDMMTCYLFHMYVGVRAGG | DITFFFFVIVILLAIIQGLII |
| A4601-S5035 |  |  |  | 4642-4662 |  | 4787-4807 | 4837-4857 | 4873-4893 | 4914-4934 |
| Seq |  |  |  | RCLSLLHTLVAFLCIIGYNCL |  | FLYLGWYMVMSLLGHYNNFFF | MTVGLLAVVVYLYTVVAFNFF | KCDDMMTCYLFHMYVGVRAGG | FDITFFFFVIVILLAIIQGLI |
| T4301-S5035 | 4321-4341 | 4344-4364 | 4556-4576 | 4640-4660 | 4772-4792 | 4794-4814 | 4834-4854 |  | 4913-4933 |
| Seq | TAREAATAVAALLWALVTRAG | GAGAAAGALRLLWGSLFGGGL | NFYTLRFLALFLAFAINFILL | ALRCLSLLHTLVAFLCIIGYN | KYQIWKFGVIFTDNSFLYLGW | MVMSLLGHYNNFFFAAHLLDI | QLVMTVGLLAVVVYLYTVVAF |  | VFDITFFFFVIVILLAIIQGL |
| Kyte Doolittle | 4324-4341 | 4344-4365 | 4560-4580 | 4640-4668 | 4777-4800 | 4803-4828 | 4835-4857 | 4879-4894 | 4912-4939 |
|  |  |  |  |  |  |  |  |  |  |
| *Dm*RyR_fl | 4437-4457 |  | 4632-4652 | 4720-4740 |  | 4863-4883 | 4906-4926 |  | 4986-5006 |
| Seq | LIVGFFKIIFYIFYYTGYAHF |  | NLKYVALVLAFSINFMLLFYK | RIAACLHSLVSLAMLIAYYHL |  | LWYFSFSVMGNFNNFFFAAHL | QLVLTVMLLTIIVYIYTVIAF | LTCFVFHLYKG | DITFFFFVIIILLAIIQGLII |
| S4601-G5112 |  |  | 4630-4650 | 4720-4740 |  | 4859-4879 | 4909-4929 |  | 4983-5003 |
| Seq |  |  | FYNLKYVALVLAFSINFMLLF | RIAACLHSLVSLAMLIAYYHL |  | FLYSLWYFSFSVMGNFNNFFF | LTVMLLTIIVYIYTVIAFNFF |  | IFFDITFFFFVIIILLAIIQG |
| S4301-G5112 | 4444-4464 |  | 4631-4651 | 4720-4740 |  | 4863-4883 | 4906-4926 |  | 4986-5006 |
| Seq | IIFYIFYYTGYAHFCVVRYIF |  | YNLKYVALVLAFSINFMLLF | RIAACLHSLVSLAMLIAYYHL |  | LWYFSFSVMGNFNNFFFAAHL | QLVLTVMLLTIIVYIYTVIAF |  | DITFFFFVIIILLAIIQGLII |
| Kyte Doolittle | 4435-4452 | 4455-4471 | 4634-4654 | 4715-4746 |  | 4854-4894 | 4908-4930 | 4949-4959 | 4983-5009 |
|  |  |  |  |  |  |  |  |  |  |
| *Ce*RyR_fl | 4440-4460 |  | 4621-4641 | 4685-4705 |  | 4824-4844 | 4867-4887 |  | 4952-4972 |
| Seq | VIILFIRAGLAIGWAGYLLLM |  | KITLYLAFFINVILLFHRVDI | ILYWISVLHLSTSFALLVSFYQLK |  | GYLLCSACGVFLSPFFYAFHL | QLILTIMMTLVVVYLYTVIAF | LTCFIYHFYAGVRAGG | FFFFVIIILLAIMQGLIIDAF |
| S4601-S5071 |  |  | 4616-4636 | 4686-4706 | 4806-4826 | 4828-4848 | 4868-4888 |  | 4949-4969 |
| Seq |  |  | FKTIEKITLYLAFFINVILLF | LYWISVLHLSTSFALLVSFYQ | CWLWIGVILTNGQFLYRVGYL | CSACGVFLSPFFYAFHLIDVV | LILTIMMTLVVVYLYTVIAFN |  | DISFFFFVIIILLAIMQGLII |
| S4301-S5071 | 4431-4451 | 4453-4473 | 4616-4636 | 4687-4707 | 4804-4824 | 4828-4848 | 4868-4888 |  | 4950-4970 |
| Seq | MSWTQLLYAVIILFIRAGLAI | WAGYLLLMTIFRFGYFLTTSS | FKTIEKITLYLAFFINVILLF | YWISVLHLSTSFALLVSFYQL | YSCWLWIGVILTNGQFLYRVG | CSACGVFLSPFFYAFHLIDVV | LILTIMMTLVVVYLYTVIAFN |  | ISFFFFVIIILLAIMQGLIID |
| Kyte Doolittle | 4436-4469 |  | 4621-4639 | 4679-4713 | 4807-4817 | 4822-4861 | 4867-4891 | 4912-4927 | 4950-4973 |

Results of transmembrane predictions using the TOPCONS algorithm (http://topcons.net/, [5]) obtained with full-length proteins (fl) or C-terminal regions as input queries. Putative transmembrane helices printed in red show slight deviations between the different queries, those printed in green represent results of one of the five topology predictions integrated in TOPCONS. The results retrieved for the CRC-III-1bΨ pseudogene are outlined in grey or bright red.

Furthermore, full-length proteins were analyzed with TMHMM v.2.0 (http://www.cbs.dtu.dk/services/TMHMM/, [6]) and Kyte Doolitte hydrophobicity scales [7]. Additionally to the *Paramecium* CRC sequences, we analyzed three different metazoan IP_3_ and ryanodine receptors, which are from *Mus musculus* (*Mm*IP_3_R type 1, Acc No: NP_034715.2; *Mm*RyR type 1, Acc No: NP_033135), *Drosophila melanogaster* (*Dm*IP_3_R, Acc No: BAA14399.1; *Dm*RyR, Acc No: NP_033135) or *Caenorhabditis elegans* (*Ce*ITR1, Acc No: NP_001023173; *Ce*RyR, Acc No: BAA08309).
