# Supplementary material for: Calcium-Release Channels in Paramecium. Genomic Expansion, Differential Positioning and Partial Transcriptional Elimination
Source: PLoS One. 2011 Nov 10;6(11):e27111. doi: 10.1371/journal.pone.0027111 (PMC3213138; doi:10.1371/journal.pone.0027111)
Supplement: Table S2 — Oligonucleotide primers used for amplification and cloning of CRC genes. (DOC) [file pone.0027111.s008.doc]

**Table S2.** Oligonucleotide primers used for amplification and cloning of CRC genes.

| **Designation** | **Sequence (5’→ 3’)** | **Application** |
| --- | --- | --- |
| **Oligonucleotide primers used for amplification and cloning of CRC-I genes** | | |
| **cDNA-analyses** | | |
| Dei-1 | aactggaagaattcgcggccgcggaattttttttttttttt | Reverse transcription |
| Sc26-141f | TTATGGAATAAATACAGAAGACAG | Determination of the start codon of CRC-I-1b |
| Sc26_70f | CATTCTTTCATAACAGCAGATGG |
| Sc26_529rev | TGCCAAATGCAATAATTAAATATG |
| Sc26-120f | CAGAATCCAAGATGACCAATGG |
| Sc98-7f | AGTCCAAAGGAAGTAGTTAATTTG | Determination of the start codon of CRC-I-1c |
| Sc98-212f | AATTATTTGACTTACGGATCTGTG |
| Sc98-705rev | TGAATGTGAGATTTATACGAAACC |
| Dei-2 | aactggaagaattcgcggccgcgg | Determination of the stop codons of |
| Sc9A_8695f | TCTCAAATACAAAGATCCCACAG | CRC-I-1a, |
| Sc26_8709f | ATCTCAAATACAAAGATCCAACAG | CRC-I-1b, |
| Sc98_8826f | AGGATAGTTCATCTATGGGTGG | CRC-I-1c |
| Sc9A_383f | GCAGGTCTAAATGTTTTCCCAG | verifying deviations of CRC-I-1a from  ParameciumDB sequences |
| Sc9A_1544rev | CAAAAGAAATTTCCCAAACGTC |
| Sc9A_3910f | TCACGAGGAAGAATTAGAAAAGAG |  |
| Sc9A_ 4427rev | TCTAATTTTCATGGTTATTCCTGC |  |
| Sc9A-8276f-NotI | gcggcggccgcTTGGTACTTTTCGTAATTCTTGTG' | cDNA analyses of CRC-I-1 genes |
| Sc9A-8985rev-NcoI | gcgccatggTTGAATGGAAACCATGTTAGATC |
| Sc26-8399f-NcoI | gcgccatggGTTATTAGATCTGTTTGGGAACC |
| Sc26-8976rev-PstI | gcgctgcagTTCTTCTATATTTTATCCGAAACG |
| Sc98-8294f-PstI | gcgctgcagTATTACTTAGCATATGGAGCCTTG |  |
| Sc98-8985rev-Acc65I | gcgggtaccATGATTCACTTTGATGTGATGATC |
| Sc20_6316f | TGAGTGAAAATAACCAAGACAAGA | cDNA analyses of CRC-I-2a |
| Sc20_7433rev | TCTTTGAATATTTGCCAAACTTC |
| **Production of CRC-I-1 specific antibodies** | | |
| Sc26_AGf | gcgccatgGGGcAAAAAAAGGAGAAAGAC | Cloning of CRC-I-1 specific antigenic region |
| Sc26_AGrev | gcgctcgagGCCTTCTTTACATTgCTTTTCATAC |
| Sc26AG_Q1f | CAAGTAAAACTcAAATTATGACATC | Mutagenesis of CRC-I-1 specific antigenic region |
| Sc26AG_Q1rev | GATGTCATAATTTgAGTTTTACTTG |
| Sc26AG_Q2f | CTTCGAAAACTcAAAATAACAAAATG |
| Sc26AG_Q2rev | CATTTTGTTATTTTgAGTTTTCGAAG |
| Sc26AG_Q3f | GATATCAAAAAcAACAGAAAGG |
| Sc26AG_Q3rev | CCTTTCTGTTgTTTTTGATATC- |
| Sc26AG_Q4f | GATTTAAAAAATTGGcAAATTGAAAG |
| Sc26AG_Q4rev | CTTTCAATTTgCCAATTTTTTAAATC |
| Sc26AG_Q4f | GATTTAAAAAATTGGcAAATTGAAAG |
| Sc26AG_Q4rev | CTTTCAATTTgCCAATTTTTTAAATC |
| Sc26AG_Q4rev | CTTTCAATTTgCCAATTTTTTAAATC |  |
|  |  |  |
| **Oligonucleotide primers used for amplification and cloning of CRC-III genes** | | |
| **cDNA-analyses** | | |
| Sc158_148f | gcgccatgggaTCACAATTGACTAAGAATGGTG | Analyses of cDNA sequences of CRC-III-1b |
| Sc158_5263rev | gcgctcgagAGCTAGTTGATCAGTTTCAGTG |  |
| Sc158_148f | ATTTTTATGATCATCCCTGTTCTC |  |
| Sc158_882rev | CGTAGATGATTTTGGTGAGTGTC |  |
| Sc158_6941f | TATTTGATGATCTTGGCTTATTTG |  |
| Sc158_8042rev | ATTTATGAATCCAAACCAGTCTTC |  |
| Sc86_4056f | AGCTTGTCTTCGATTAATAGGGT | cDNA analyses of CRC-III-1a |
| Sc86_4777rev | TTCTCGTGAATTAACAACAACTTC |  |
| Sc35_1743f | TTGGATGAATTTGAACTTTATCAG | cDNA analyses of CRC-III-2 |
| Sc35_2344rev | CAAAAACTCTGTACTCAACCAGAA |  |
| Sc17_7870f_NotI | gcggcggccgcTATGATTGTAAATGGTTGGGATAG | cDNA analyses of CRC-III-4b |
| Sc17_8415rev_NheI | gcggctagcTTTCATCTTCATCTCAATTACCTC |  |
| Sc49_7913f_NheI | gcggctagcGTTGTAAATGGTTGGGATAGAGTC | cDNA analyses of CRC-III-4a |
| Sc49_8433rev_PstI | gcgctgcagCCTCCTGAATTTCATTACCTTTC |  |
| Sc37_7907f_PstI | gcgctgcagTATGATAAACAATCCATGGGATAG | cDNA analyses of CRC-III-4c |
| Sc37_8452rev_Acc65I | gcgggtaccTTTCATTTTCATTTCTATTGCTTC |  |
| **Production of CRC-III-4b specific antibodies** | | |
| Sc17-AGf | gcgccatgggaTTGCATGAAcAGTCTGTAAAACTTG | Cloning of CRC-III-4b specific antigenic region |
| Sc17-AGrev | gcgctcgagTTgTTgTTCATCTTTTTCCTTGCTC |  |
| Sc17-Q1f | AGGTCCCATAcAGAAAAAGAATTC | Mutagenesis of CRC-III-4b specific antigenic region |
| Sc17-Q1rev | GAATTCTTTTTCTgTATGGGACCT |  |
| Sc17-Q2f | AGAGGAATTGGcAAACAGATAATG |  |
| Sc17-Q2rev | CATTATCTGTTTgCCAATTCCTCT |  |
|  |  |  |
| **Oligonucleotide primers used for amplification and cloning of CRC-IV-3 genes** | | |
| Sc24_85f | ATACCTCTACCGTAAATTGTGGAG | cDNA analyses of CRC-V-4a |
| Sc24_543rev | TTTCATTTATTTCTTCTTCGCAC |  |
|  |  |  |
| **Oligonucleotide primers used for amplification and cloning of CRC-V-4 genes** | | |
| **cDNA analyses** | | |
| Sc96_17f | ATGGGTAAATTGTAAGAATTCAGG | cDNA analyses of CRC-V-4a |
| Sc96_457rev | TACTGCTCAGACTGACTTGAAATG |  |
| Sc96_4112f | AGATAGAGGAGACAAAGCTAATCC |  |
| Sc96_4618rev | TCCAATTCCGATAAGAAATTATC |  |
| Sc96_4077f_NotI | gcggcggccgcTGAATTAATTGACGATTACAGTGC |  |
| Sc96_4705rev_PstI | gcgctgcagATCTTCTAAATCTGAACCAAATGG |  |
| Sc106_3940f_PstI | gcgctgcagTGCTTCAGCAGAAGTAGAATAGG | cDNA analyses of CRC-V-4b |
| Sc106_4646rev_XhoI | gcgctcgagTTCTGAGACTCTCGACATCTTTG |  |
| Sc106_4094f | TGCTAATTATGAGAAGATGGAGG |  |
| Sc106_4568rev | TCGAATACAATATCTCCTGCATAC |  |
| **Production of CRC-V-4a specific antibodies** | | |
| Sc96Ag_f | cgcccatggCCAGCTTAGAGGATcAAAAGGGAGAG | Cloning of CRC-V-4a specific antigenic region |
| Sc96Ag_rev | cgcctcgagATAGGAATCTTgCTCAGTTTTGTC |  |
| Sc96Ag_Q1f | CATTAGGATCAATcAAATGGTAGCA | Mutagenesis of CRC-V-4a specific antigenic region |
| Sc96Ag_Q1rev | TGCTACCATTTgATTGATCCTAATG |  |
| Sc96Ag_Q2Q3f | CTGAAGAACAAcAAcAATTATTAGAC |  |
| Sc96Ag_Q2Q3rev | GTCTAATAATTgTTgTTGTTCTTCAG |  |
|  |  |  |
| **Oligonucleotide primers used for amplification and cloning of CRC-VI-2 genes** | | |
| **cDNA analyses** | | |
| Sc18_6517f_NotI | gcg**gcggccgc**ATAGGAAGTGACTACCGAGTTAGC | cDNA analyses of CRC-VI-2a |
| Sc18_7234rev_PstI | gcg**ctgcag**TCACTTGAGAACAGGATCATTAGA |  |
| Sc44A_6557f_PstI | gcg**ctgcag**TGGGACTCCTTTATAATAGAGACG | cDNA analyses of CRC-VI-2b |
| Sc44A_7283rev_XhoI | gcg**ctcgag**GTTAATCCAAACAAAATATCTCCG |  |
| **Production of CRC-VI-2a specific antibodies** | | |
| Sc18-AGf | gcgccatgggaATTATTCTAAAAcAGCAATGCATTG | Cloning of CRC-VI-2a specific antigenic region |
| Sc18-AGrev | gcgctcgagATTCTgAATTTGTTgAATAATTTCAAAC |  |
| Sc18-Q1f | GCAAATTTAATcAATTTGATGACT | Mutagenesis of CRC-VI-2a specific antigenic region |
| Sc18-Q1rev | AGTCATCAAATTgATTAAATTTGC |  |
| Sc18-Q2f | CTTATTAGATAATcAAAATTTAGG |  |
| Sc18-Q2rev | CCTAAATTTTgATTATCTAATAAG |  |
|  |  |  |
| **Oligonucleotide primers used for amplification and cloning of CRC-VI-3** | | |
| **cDNA analyses** | | |
| Dei-1 | aactggaagaattcgcggccgcggaattttttttttttttt | Reverse transcription |
| Sc134del_+3f | TTGGATACTCAATATGATGATTGC | Determination of the start codon of CRC-VI-3 |
| Sc134del_-243f | ATACTAAACCGGATGATCAATTTC |
| Sc134del_-352f | GGGCTTAGATTAAATCATCATACG |
| Sc134del_221rev | AAGATGTCAGTCGAATGATTAAGC |
| Sc134del_-743f | AAGATCTGTTGCTATGGAAATACG |
| Sc134I_-69f | TAATTAGATAGGAGTGATTTCGGC |  |
| Sc134I_36f | AACCAAACATTCAAGTCTTAGGAC |  |
| Sc134I_756rev | GGGAATCTCTTCTCTGTGAAAAG |  |
| Dei-2 | aactggaagaattcgcggccgcgg | Determination of the stop codons of CRC-VI-3 |
| Sc134I_5892f -3' | TAATAGACACATTTGCGGAACTG |  |
| Sc134del_2439f | ATTCTTGATAGAAACCATTGACAG | cDNA analyses of CRC-VI-3 |
| Sc134del_2679rev | GGAGTACTATGATATTCAAGGCAG |  |
| Sc134I_5490rev | TAAAAACAGAAGGGTTCAAAGG |  |
| Sc134I_700f | GGATGAATAAAATTTGAATGAAAA |  |
| Sc134I_1860rev | TCAGCCTTAAAGTTTTCAACTTG |  |
| Sc134I_2629f | TGAATCACGATTATCTACAATTCC |  |
| Sc134I_3454rev | GCATTCTACTCCAATATATCGTTG |  |
| Sc134I_3397f | TATTGAAATTTAAACTGAAGCGG |  |
| Sc134I_4209rev | TTCCATACTAATCCCCTTGAGTAC |  |
| Sc134I_4128f | AAGAACAGTCAGAAGCAAACAAG |  |
| Sc134I_4997rev | GTCAAATATGGATGCCAACTTAG |  |
| Sc134I_5431f | CGCgcggccgcCTAATTTTTCTTGCATTTTGTCC |  |
| Sc134I_6044rev | CGCggtaccGACTATGAACTTCATCCACATGC |  |
| **Production of CRC-VI-3 specific antibodies** | | |
| Sc134I_AGf | gcgccatgGggACCATATTAAATcAGGATTTTATA | Cloning of CRC-VI-3 specific antigenic region |
| Sc134I_AGrev | gcgctcgagAAGTACTTGTTGAGTCTgTTTTATG |  |
| **Cloning of N-terminal GFP-fusion construct** | | |
| Sc134I_MluI-f | GCGacgcgtATGTTGAATAGTAGAAGATCACAA |  |
| Sc134I_XhoI-rev | GCGctcgagTCATAACTTATCGAGTAACTATTG |  |
